# Quantifying Through‐Space Substituent Effects

**DOI:** 10.1002/anie.202006943

**Published:** 2020-07-17

**Authors:** Rebecca J. Burns, Ioulia K. Mati, Kamila B. Muchowska, Catherine Adam, Scott L. Cockroft

**Affiliations:** ^1^ EaStCHEM School of Chemistry University of Edinburgh Joseph Black Building David Brewster Road Edinburgh EH9 3FJ UK

**Keywords:** electrostatic interactions, noncovalent interactions, substituent effects

## Abstract

The description of substituents as electron donating or withdrawing leads to a perceived dominance of through‐bond influences. The situation is compounded by the challenge of separating through‐bond and through‐space contributions. Here, we probe the experimental significance of through‐space substituent effects in molecular interactions and reaction kinetics. Conformational equilibrium constants were transposed onto the Hammett substituent constant scale revealing dominant through‐space substituent effects that cannot be described in classic terms. For example, NO_2_ groups positioned over a biaryl bond exhibited similar influences as resonant electron donors. Meanwhile, the electro‐enhancing influence of OMe/OH groups could be switched off or inverted by conformational twisting. 267 conformational equilibrium constants measured across eleven solvents were found to be better predictors of reaction kinetics than calculated electrostatic potentials, suggesting utility in other contexts and for benchmarking theoretical solvation models.

## Graphical abstract

**The description of substituents** as electron donating or withdrawing leads to a perceived dominance of through‐bond influences. The situation is compounded by the challenge of separating through‐bond and through‐space contributions. Measurements of through‐space substituent effects question classic descriptions of substituent effects.

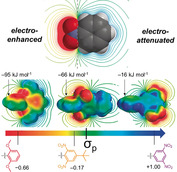

## Introduction

Systematic variation of substituents is often exploited to tune or rationalize chemical behavior and reaction mechanisms.^1^ Substituent effects are usually ranked using relative electronegativities and empirically derived substituent constants.^2^ Although the quantification of substituent effects has a long history,^3^ it was Hammett who defined and established the transferability, and thus great utility of quantitative *σ*_m_ and *σ*_p_ substituent constants determined from the p*K*_a_ values of benzoic acid derivatives.^2^, ^4^ However, it was soon realized that transferrable *σ*_o_ constants could not be easily defined due to interactions occurring between *ortho* substituents.^2^, ^5^ Similarly, the lexicon of classifying substituents as either “electron withdrawing” or “electron donating” emphasizes bond connectivity and the through‐bond contributions of induction and resonance. As a result, there is an unconscious tendency to overlook the significance of electrostatic (field) contributions that occur through space. Indeed, the dissection of through‐bond and through‐space contributions to substituent effects has been a long‐term challenge.^6^

Through‐bond resonant contributions often dominate the behavior of delocalized systems and are therefore relatively easy to dissect from the combined inductive and field effect (e.g. R from *σ*_m/p_ or R^−^ and R^+^ from *σ* ^+^ and *σ* ^−^).^2^, 4b, 7 In contrast, inductive and field effects have historically been treated together due to the difficulty in separating them in experimental systems.7a, 8 Nonetheless, inductive and field effects, specifically those occurring along the direction of a bond, have been described by substituent constants such as *F*, *σ*_I_ and *σ*_F_.^2^, 5b, 9 Such was the proliferation of studies aiming to complement Hammett's seminal constants, that at least twenty different dissected scales had been defined by the 1970s.^8^, 9b Swain, Leo and Taft criticized many of these dissections for making incorrect assumptions about the transferability of field effects, pointing out that field effects are intrinsically spatially dependent and likely to dominate over through‐bond effects for distant substituents.^2^, 9b The Kirkwood–Westheimer model can be credited as being one of the earliest methods for estimating the geometric influence of substituent‐induced dipoles.3a, 10 The model can estimate substituent effects on acid dissociation constants, but with imperfect applicability.7a, 9c, 11 More recent examinations of substituent effects on dissociation constants have seemingly side‐stepped the Kirkwood‐Westheimer model in favor of contemporary computational approaches.^12^ Early work by Topsom showed that through‐bond and through‐space substituent effects could be dissected by deleting bonds separating a substituent and a site of interest.^13^ More recently, Wheeler, Houk and Suresh have found that additive substituent field effects can largely account for the calculated electrostatic potentials of aromatic rings.6a, 14 Such through‐space models are beginning to supersede earlier empirically derived models of aromatic interactions,^15^ and numerous investigations have highlighted the importance of field effects in enzyme‐catalyzed reactions^16^ and synthetic organic chemistry.^17^ However, contrasting with the success of empirically derived Hammett substituent constants in accounting for reactivity in a wide range of contexts, high‐quality experimental data quantifying through‐space substituent effects are surprisingly limited.

**Figure 1:**
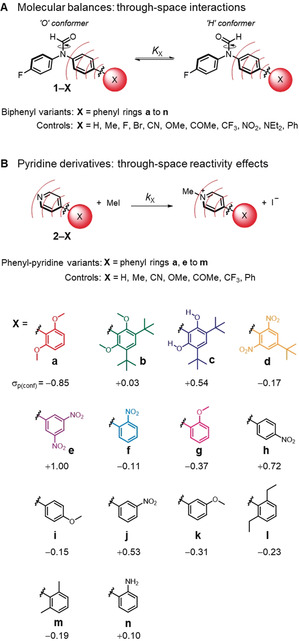
A) Molecular balances (**1‐X**) and B) pyridine derivatives (**2‐X**) used in the present investigation to quantify through‐space substituent effects on molecular interactions and reaction kinetics, respectively. The values listed under the structures of substituents **a** to **n** are the Hammett constants, *σ*_p(conf)_ determined from conformational equilibrium constants measured in [D_6_]benzene at 298 K (Table 1) using the correlation shown in Figure 2 B. Errors in *σ*_p(conf)_<±0.08 (see section S4 in SI). Color coding matches the use in subsequent figures.

**Figure 2:**
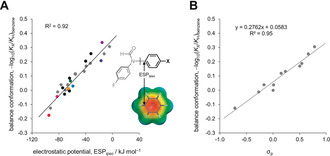
A) Correlation between the calculated electrostatic potential in the position indicated (ESP_ipso_) and the conformational equilibrium constants determined in [D_6_]benzene at 298 K for the **1**‐**X** series of 25 molecular balances shown in Figure 1 A. ESPs were calculated using B3LYP/6‐31G* on the 0.002 electron/Bohr^3^ isosurface. Electrostatic potentials determined using isolated (proton‐capped) X‐substituents (i.e. without through‐bond contributions) also correlated highly with the experimental data (R_2_=0.89, Figure S1C). B) Correlation between known *σ*_p_ Hammett substituent constants and conformational equilibrium constants of balances **1‐X** determined in [D_6_]benzene at 298 K. Errors in −log_10_(*K*_X_/*K*_H_) are <±0.08 (section S4 in SI).

**Figure 3:**
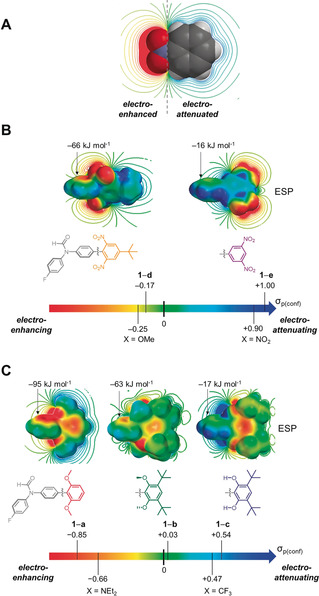
A) Calculated electrostatic potential slice showing electro‐enhanced (δ−ve) and electro‐attenuated (δ+ve) regions in space surrounding nitrobenzene. B) Experimentally determined Hammett substituent constants *σ*_p(conf)_ quantified using the conformational preferences of series **1**‐**X** demonstrate switching from electro‐enhancing to electro‐attenuating behavior upon changing the orientation of a nitro group. C) The strongly electro‐enhancing behavior of methoxy groups (left) can be switched off via a conformational twist induced by adjacent *tert*‐Bu groups (center). In contrast, hydroxyl groups in the same position exert a strong electro‐attenuating influence (right). Electrostatic potentials are scaled from −100 kJ mol^−1^ (red) to +100 kJ mol^−1^ (blue). Indicated electrostatic potential values correspond to ESP_ipso_ as defined in Figure 2 A at the positions indicated with arrows.

**Figure 4:**
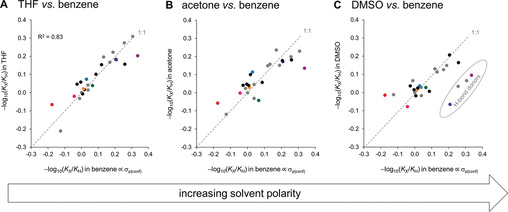
Effect of increasing solvent polarity on the conformational equilibrium constants −log_10_(*K*_X_/*K*_H_) determined at 298 K for the **1‐X** series of molecular balances shown in Figure 1. Data for eleven solvents are reported in Table 1.

**Figure 5:**
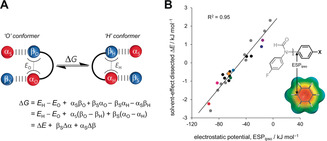
A) Energetic contributions to the difference in free energy between two conformations of a molecular balance, Δ*G* where solvophobic effects are negligible. *E*_O_ and *E*_H_ correspond to the intramolecular interactions in the O‐ and H‐conformers, respectively; α_Ο_, α_H_, α_S,_ β_Ο_, β_H_ and β_S_ are the hydrogen‐bond donor (α) and acceptor constants (β) of the O‐/ H‐conformers and the solvent, respectively.21a, 27 B) Correlation of calculated electrostatic potentials over the *ipso*‐carbon ESP_ipso_ vs. the solvent‐independent intramolecular interaction energy difference Δ*E*=*E*_H_−*E*_O_ dissected using the same solvation model.

**Figure 6:**
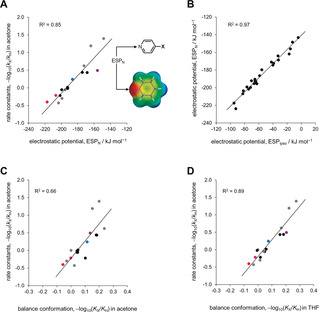
A) Relationship between calculated electrostatic potentials taken over the nitrogen atom (ESP_N_) and the *N*‐methylation of the 17 pyridine derivatives shown in Figure 1 B in [D_6_]acetone at 298 K. ESPs were calculated using B3LYP/6‐31G* on the 0.002 electron/Bohr^3^ isosurface. B) Correlation of electrostatic potentials in X‐substituted phenyl derivatives (ESP_ipso_) vs. corresponding X‐substituted pyridine derivatives (ESP_N_). C) Correlation of conformational equilibrium constants measured in the **1**‐**X** balance series vs. rate constants for the *N*‐methylation of correspondingly substituted **2**‐**X** pyridine derivatives, when both sets of measurements were performed in [D_6_]acetone. D) Improved correlations were found between rate constants measured in [D_6_]acetone and conformational equilibrium constants measured in five other solvents including tetrahydrofuran (R^2^=0.88 to 0.94, Figures S38–S39). All experiments were performed at 298 K.

Here we have used 25 molecular balances and 17 pyridine derivatives to quantify the importance of through‐space substituent effects on molecular interactions and reaction kinetics, respectively (Figure 1). The experimentally observed equilibrium and kinetic constants were correlated with calculated electrostatic potentials (ESPs) to examine the experimental significance of through‐space substituent effects (Figures 2, 3 and 6). Transposing the experimentally observed conformational equilibrium constants onto the Hammett substituent constant scale (Figure 2, Table 1) revealed the remarkable extent to which through‐space effects can dominate experimental behaviour compared to more classically considered through‐bond contributions (Figure 1 and Figure 3). The transferability of Hammett substituent constants derived from conformational equilibrium constants was examined across eleven different solvents (Table 1, Figure 4 and Figure 5), and their ability to account for through‐space influences on the kinetics of a simple model reaction was assessed (Figure 6).

**Table 1:** Relative conformational equilibrium constants −log_10_(*K*_X_/*K*_H_) determined using the molecular balances shown in Figure 1 in eleven solvents at 298 K. The −log_10_(*K*_X_/*K*_H_) values determined in [D_6_]benzene were transposed onto the standard Hammett *σ*_p_ scale using the calibration graph shown in Figure 2 B, and the resulting *σ*_p(conf)_ values listed under the structures shown in Figure 1 B.

	−log_10_(*K*_X_/*K*_H_)										
Compound	[D_6_]benzene	[D_6_]DMSO	[D_6_]acetone	EtOAc^[a]^	[D_8_]THF	[D_3_]MeCN	CDCl_3_	[D_2_]DCM	EtOH^[a]^	[D_4_]MeOH	Diethyl ether^[a]^
**1‐NEt_2_**	−0.12	−0.01	−0.12	−0.19	−0.21^[a]^	−0.04	−0.08	−0.05^[a]^	−0.12	−0.04	−0.23
**1‐OMe**	−0.01	+0.07	+0.02^[a]^	+0.03	−0.03^[a]^	+0.05	+0.06	+0.04^[a]^	+0.02	+0.05^[a]^	−0.03
**1‐H**	0.00	0.00	0.00	0.00	0.00^[a]^	0.00	0.00	0.00^[a]^	0.00	0.00	0.00
**1‐Me**	+0.04	−0.02	−0.03	+0.05	+0.01	−0.02	−0.05	+0.07	−0.02	−0.01	−0.03
**1‐Ph**	+0.04	+0.03	+0.05	+0.07	+0.04^[a]^	+0.03	+0.08	+0.04	+0.05	+0.03	+0.08
**1‐F** ^[b]^	+0.12	+0.13	+0.15	+0.19	+0.15	+0.14	+0.20	+0.14	+0.14	+0.14	+0.19
**1‐Br**	+0.13	+0.11	+0.16	+0.22	+0.20^[a]^	+0.11	+0.21	+0.16^[a]^	+0.15	+0.13	+0.26
**1‐COCH_3_**	+0.17	+0.13	+0.24	+0.19	+0.17	+0.11	+0.21	+0.17	+0.17	n.r.^[d]^	+0.25
**1‐CF_3_**	+0.19	+0.15	+0.15	+0.27	+0.22	+0.19	+0.28	+0.22	+0.16	n.r.	+0.39
**1‐CN**	+0.26	+0.03	+0.20^[a]^	+0.30	+0.27^[a]^	+0.11	+0.38	+0.27^[a]^	+0.21	+0.17	+0.42
**1‐NO_2_**	+0.31	+0.07	+0.23^[a]^	+0.35	+0.31^[a]^	+0.14	+0.44	+0.32^[a]^	+0.25	+0.17	+0.54
											
**1‐a**	−0.18	−0.01	−0.06	−0.03	−0.07	−0.01	−0.19	+0.03	−0.06	−0.06	−0.27
**1‐b**	+0.07	+0.03	−0.04	+0.09	+0.04	+0.01	−0.02	−0.03	n.s.^[c]^	+0.06	+0.06
**1‐c**	+0.21	−0.07	+0.17	+0.19	+0.18	+0.17	+0.33	+0.15	+0.30	+0.20	+0.29
**1‐d**	+0.01	+0.02	+0.03	+0.09	+0.02	+0.01	+0.02	+0.09	n.s.	+0.03	+0.09
**1‐e**	+0.34	+0.10	+0.14	+0.25	+0.20	+0.13	+0.31	+0.21	n.s.	+0.19	n.s.
**1‐f**	+0.03	+0.04	+0.11	+0.09	+0.07	+0.06	+0.13	+0.07	+0.10	+0.02	+0.13
**1‐g**	−0.04	−0.08	0.00	+0.02	−0.02	+0.02	−0.09	−0.01	−0.01	+0.02	0.00
**1‐h**	+0.26	+0.17	+0.18	+0.23	+0.16	+0.20	+0.23	+0.16	+0.18	+0.20	+0.21
**1‐i**	+0.02	+0.03	+0.10	+0.07	+0.02	−0.03	+0.08	+0.03	n.r.	+0.03	+0.09
**1‐j**	+0.20	+0.21	+0.18	+0.20	+0.18	+0.20	+0.21	+0.19	+0.20	+0.22	n.s.
**1‐k**	−0.03	+0.04	+0.04	+0.08	+0.05	+0.03	+0.01	−0.02	+0.04	+0.01	+0.13
**1‐l**	0.00	+0.02	+0.05	+0.08	+0.06	−0.01	−0.02	−0.04	+0.04	0.00	+0.11
**1‐m**	+0.01	−0.02	+0.04	+0.10	−0.01	+0.03	+0.01	0.00	+0.07	−0.02	−0.02
**1‐n**	+0.09	+0.01	+0.06	+0.03	+0.10	+0.08	+0.12	+0.04	+0.05	+0.11	+0.03

[a] Values obtained in non‐deuterated solvent. [b] Hypothetical compound, *K*_X_=1 due to symmetry. [c] n.s.=insufficient solubility. [d] n.r=distinct conformer peaks not resolved by ^19^F or ^1^H NMR at 298 K.

## Results and Discussion

### Design of experimental systems for quantifying through‐space substituent effects

The separation of through‐bond and through‐space substituent effects is known to be challenging. While computational methods are extremely useful in facilitating the quantitative dissection of substituent effects, they often model situations that are difficult, or even impossible to probe experimentally (e.g. functional groups held in specific spatial orientations, or functional group dissections that do not exist). Similarly, the computational prediction of solvent effects remains notoriously difficult.^18^ Therefore, for our experimental investigations in solution, we instead sought model systems in which through‐space effects would dominate over those occurring through bonds. The compounds shown in Figure 1 contain biphenyl and 4‐phenylpyridine units that position substituents in similar geometries, allowing comparisons to be drawn between influences on interactions and reaction kinetics. We reasoned that through‐space effects would be most likely to dominate over through‐bond influences when polar substituents were positioned *ortho* to the biaryl bond. Such frameworks also allow systematic variation of the positioning of substituents and an assessment of their influence on non‐covalent interactions and chemical reactions occurring at a remote location. We selected uncharged substituents for this investigation to avoid counterion and solubility issues during solvent‐screening experiments.

### Through‐space substituent effects on conformational equilibria

Synthetic molecular balances of the type shown in Figure 1 A were adopted to examine the through‐space influence of substituents on non‐covalent interactions. Molecular balances provide useful tools for such an investigation since substituent effects perturb the position of a conformational equilibrium in a quantifiable manner.^19^ Indeed, variants of the balances shown in Figure 1 A have previously been used to probe substituent and solvent effects in carbonyl‐carbonyl interactions, and hydrogen and chalcogen bonds.^20^ Slow rotation of the formamide C−N bond on the NMR timescale allows the integration of discrete ^19^F NMR signals corresponding to each conformer. The integral ratio corresponds to the conformational equilibrium constant, *K*_X_, which constitutes a quantitative assessment of the substituent effects on interactions occurring within the balances. Negative electrostatic potential over the X‐substituted ring would repel the electron‐rich carbonyl oxygen and contribute towards a preference for the H‐conformer in Figure 1. In contrast, positive electrostatic potential over the X‐substituted ring would help to stabilize the O‐conformer. In addition, we reasoned that an apolar solvent such as benzene would provide the best opportunity for the manifestation of through‐space substituent effects. Thus, the conformational equilibrium constants, *K*_X_ for the 25 molecular balances shown in Figure 1 A were determined in [D_6_]benzene (Table 1).

The Hammett‐style relationship −log_10_(*K*_X_/*K*_H_), encodes the electronic effects of the X substituent on the position of the conformational equilibrium. These −log_10_(*K*_X_/*K*_H_) values are accordingly positive for classically “electron‐withdrawing” *para*‐substituents (X=Br, CN, NO_2_, CF_3_) and negative for “electron‐donating” *para*‐substituents (X=OMe, NEt_2_). Such substituent effects are reflected in the calculated electrostatic potentials taken over the carbon positioned *ipso* to the conformationally exchanging formamide (ESP_ispo_, Figure 2 A). These electrostatic potentials correlate strongly with −log_10_(*K*_X_/*K*_H_) for all 25 molecular balances in benzene (R^2^=0.92 in Figure 2 A).

The experimentally determined conformational equilibrium constants can be transposed onto the established σ_p_ Hammett substituent constant scale since these values are known for the X substituents in the eleven control balances (Figure 2 B and Table 1).^2^, ^21^ Hence, the correlation shown in Figure 2 B can be used to determine the Hammett constants for the more unusual phenyl substituents **a** to **n** (*σ*_p(conf)_). The 3,5‐dinitrophenyl substituent in compound **1‐e** (purple, Figure 3 B), which positions two nitro groups *meta* to the biphenyl bond, was found to have the most positive *σ*_p(conf)_=+1.00. Such behavior would classically be described as more electron‐withdrawing than the directly connected nitro substituent in compound **1‐NO_2_** (*σ*_p(conf)_=+0.90). Strikingly, moving the two nitro groups such that they are positioned over the biaryl bond in compound **1**‐**d**, results in a very large change in the determined Hammett substituent constant (*σ*_p(conf)_=−0.17, orange in Figure 3 B). Part of this difference in *σ*_p(conf)_ can be attributed to the addition of a *tert*‐butyl group, but the *σ*_p_=−0.20 for a *tert*‐butyl group,^2^ which is even further diminished by the intermediary phenyl ring, makes a minor contribution to the total change in *σ*_p(conf)_ of −1.17 between **1‐d** and **1‐e**.

Calculated electrostatic potentials (ESPs) provide insight into the large changes in substituent effects upon repositioning the nitro groups (Figures 3 A,B). There are large differences in the ESP values taken over the ring bridging between the X substituent and the formyl group due to geometric differences in the through‐space influences of the nitro‐groups (ESP_ipso_=−66 vs. −16 kJ mol^−1^, Figure 3 B). The major difference here arises from the electron‐rich oxygen atoms of nitro groups being positioned over the bridging ring in compound **1‐d**. Indeed, despite the large electrostatic change arising from differences in the proximity of the nitro oxygen atoms, these ESP_ipso_ values remain excellent predictors of the conformational preferences of the balances (orange and purple points in Figure 2).

Strong through‐space substituent effects are not limited to the nitro group. Given a *σ*_p(conf)_=−0.85, it might be tempting to describe the 2,6‐dimethoxyphenyl substituent in **1‐a** as being even more “electron donating” than the amino group in **1‐NEt_2_** (*σ*_p(conf)_=−0.66, red in Figure 3 C). Such striking through‐space electronic influences likely account for the prevalence of proximally positioned OR and NR groups in ligands widely exploited in catalysis (e.g. SPhos, SagePhos, BI‐DIME).^22^ Interestingly, the strong electro‐enhancing influence of the 2,6‐dimethoxy groups was found to be completely switched off in compound **1‐b** (*σ*_p(conf)_=+0.03, green in Figure 3 C). This effect arises from the installation of *ortho tert*‐butyl groups, which sterically twist the OMe groups such that the oxygen lone pairs no longer point over the adjacent phenyl ring. Accordingly, the ESP_ipso_ value over the bridging phenyl ring in **1‐b** was similar to the control compound **1‐H**, which does not contain any OMe substituents (63 vs. 70 kJ mol^−1^, Figure 3 C).

Remarkably, removing the capping methyl groups from compound **1‐b** to give the dihydroxyphenyl compound **1‐c** further shifted *σ*_p(conf)_ from +0.03 to +0.54 (Figure 3 C, right). The 2,6‐dihydroxyphenyl substituent in compound **1‐c**, thus displays a similar electronic influence as the strongly electron‐withdrawing trifluoromethyl group in control compound **1‐CF_3_** (*σ*_p(conf)_=+0.47). The electrostatic slices in Figure 3 C show that the effect arises due to the electron‐poor protons of the OH groups being positioned over the bridging phenyl ring in compound **1‐c**.

Across the series of **1‐a** to **1‐c**, ESP_ipso_ on the bridging ring changes by 78 kJ mol^−1^ and *σ*_p(conf)_ changes by ≈1.4 units (Figure 3 C and 2 A). Notably, this trend runs counter to expectations based on traditional through‐bond considerations; the sums of the Hammett constants for the substituents bonded to the terminal phenyl ring equal −0.54, −0.74, and −0.94 for balances **1‐a**, **1‐b**, and **1‐c**, respectively. Specifically, **1‐c**, which contains the strongest through‐bond electron donors behaves like a strong “electron withdrawing” group, whereas **1‐a**, which contains the weakest through‐bond electron donors is the only compound of this trio that actually behaves like an “electron donor”.^2^, ^23^

The observation of substituent effects running counter to through‐bond expectations, combined with the ability to switch such substituent effects on or off via conformational change, is consistent with through‐space field effects playing a dominant role in governing the conformational preferences of the balances shown in Figure 1 A. This hypothesis is supported by several additional observations:

- Hammett constants determined in the mono‐*ortho* nitro and methoxy compounds **1‐f** and **1‐g** were approximately half that of the corresponding di‐*ortho*‐substituted analogs **1‐d** and **1‐a** (*σ*_p(conf)_=−0.11 and −0.37 vs. −0.17 and −0.85). Again, the negative sign of *σ*_p(conf)_ for the *ortho*‐nitrophenyl substituent runs counter to the traditional expectation that a nitro group is strongly electron withdrawing.
- Positioning an amino group over the biaryl bond (**1‐n**) gave *σ*_p(conf)_=+0.11, with the through‐space δ+ charge of the NH protons overcoming through‐bond resonant donor ability of the nitrogen lone pair (Figure S25).
- The *meta*‐methoxyphenyl substituent in **1‐k** (*σ*_p(conf)_=−0.31) exhibited an electro‐enhancing influence even though *meta*‐methoxy groups normally have net electron‐withdrawing character (*σ*_p_=+0.12).^2^ Surprisingly, this effect is not observed for the *meta*‐nitrophenyl substituent (**1‐j**). However, ESP slices reveal that the bridging phenyl ring is located in the electro‐attenuated region when bonded to a *meta*‐nitrophenyl group, but in the electro‐enhanced region for the *meta*‐methoxyphenyl example (Figures S21,S22).
- The *para*‐substituted phenyl rings of balances **1‐h** and **1‐i** project their NO_2_ and OMe substituents along the same axis as the simple control balances **1‐NO_2_** and **1‐OMe**. Accordingly, the signs and magnitude of their respective *σ*_p(conf)_ values (+0.72 and −0.15) are consistent with their substituent effects being projected in the same direction, but over a greater distance than the aforementioned control balances (*σ*_p(conf)_=+0.72 vs. +0.90, and −0.15 vs. −0.25).
- Apolar methyl and ethyl groups positioned *ortho* to the biphenyl bond (**1‐l** and **1‐m**) gave similar substituent effects to the control compounds **1‐H**, **1‐Me**, and **1‐Ph**. Such findings also rule out electronic changes arising from “steric pressure”.^24^

Our observations are in accord with Wheeler and Houk's proposal that through‐space field effects, and not through‐bond polarization of the aryl π‐system, are the major determinants of non‐covalent interactions involving aromatic rings.14a, 14b, 14c Such a hypothesis is supported by NMR spectroscopic analysis of the molecular balances depicted in Figure 1. Substituent‐induced changes in the electron density of the central biphenyl ring would be anticipated to manifest as chemical shift changes.^25^ However, in stark contrast with the strong electrostatic correlations in Figures 2 A and S1C, no correlation was observed between the chemical shifts of the protons on the central biphenyl ring and the conformational preferences of the balances (Table S4). Moreover, application of Wheeler and Houk's deletion and proton‐capping approach^14^ reveals that the ESP_ipso_ values utilized in Figures 2 A and S1C correlate strongly with the electrostatic potential determined for the isolated (proton‐capped) X‐substituents at the point in space occupied by the formyl oxygen in the O conformer of each balance (R^2^=0.94, Figure S1D). Similarly, the same through‐space electrostatic potentials, in which through‐bond contributions are definitively absent, also correlated well with the experimental conformational equilibrium constants (R^2^=0.89, Figure S1C).

Having convinced ourselves of the dominance of through‐space substituent effects over through‐bond polarization of the aryl π‐system, we next sought to examine whether such dominance is manifested in other contexts; namely solvent and reactivity influences.

### Solvent effects on through‐space substituent effects

The success of Hammett substituent constants for rationalizing electronic effects can be attributed to their established transferability. Hammett constants are often applicable in contexts far beyond the original defining system (p*K*_a_ values of benzoic acids in aqueous solution), even though the electronic effects of substituents can be modulated by the solvent.20a, 21c The fluorophenyl group contained within the molecular balances employed in the present study (Figure 1) enable systematic screening of the solvent influences on substituent effects. Hence, the −log_10_(*K*_X_/*K*_H_) values for 267 substituent/solvent combinations were determined using ^19^F NMR spectroscopy spanning 25 molecular balances in eleven different solvents (Table 1). Many of the −log_10_(*K*_X_/*K*_H_) values of the simple control balances appear to be relatively insensitive to the solvent (the gray points in Figure 4 lie close to the 1:1 line). The −log_10_(*K*_X_/*K*_H_) values for the most polar substituent/solvent combinations deviate most strongly from the 1:1 line. For example, outliers in dimethylsulfoxide (DMSO) include compound **1‐a** (red point), and those containing good H‐bond donors (ringed in Figure 4 C).

Hunter's α/β hydrogen‐bond model has found use in accounting for the influence of solvents on the conformational preferences of molecular balances.20b, 26 We used the same model employed in this previous work to rationalize that the conformational free energies Δ*G* of the formyl balances would be governed by: i) differences in the intramolecular interactions between the O‐ and H‐conformers (Δ*E*), and ii) the change in Boltzmann averaged hydrogen‐bond donor and acceptor constants between each conformer (Δα and Δβ), as represented in Figure 5 A. The 267 experimental conformational free energies (determined from Δ*G*=−*RT*ln*K*_X_ as the α_s_ and β_s_ solvent H‐bond constants were varied) were fitted to the equation in Figure 5 A (Section S7 in SI, Figure S40, R^2^=0.85). Pleasingly, the dissected solvent‐independent Δ*E* values (Table S10) gave an improved correlation against the electrostatic potential over the *ipso*‐carbon (ESP_ipso_, Figure 5 B, R^2^=0.95) compared to the prior correlation against equilibrium constants determined in [D_6_]benzene (Figure 2 A, R^2^=0.92). The improved correlation reaffirms the dominance of electrostatics in determining the conformational preferences of these molecular balances, while also showing that a simple empirical solvation model can partially account for the attenuating influence of the surrounding solvent.

### Through‐space substituent effects on reaction kinetics

Having examined the transferability of the through‐space substituent effects in different solvents, we next sought to determine the significance of such effects on reaction kinetics. Inspired by previous work,^27^ we selected the *N*‐methylation of substituted pyridines with methyl iodide as a model reaction (Figure 1 B). Such an investigation presented several experimental challenges. Firstly, the synthesis of phenyl pyridyl derivatives proved to be more challenging than the equivalent biphenyl molecular balances, particularly for the sterically hindered and electron‐poor examples. Secondly, certain substituents and some polar solvents could not be used since they react with methyl iodide. Additionally, inert apolar solvents did not offer good solubility across the full range of pyridine derivatives and *N*‐methylated iodide products that we sought to examine. Eventually, we settled on [D_6_]acetone as our solvent of choice for this part of our investigation. The rate constants *k*_X_ of *N*‐methylation at 298 K were determined under pseudo‐first order conditions as the X‐substituent was varied. ^1^H NMR spectroscopy was used to monitor the relative integrals of the pyridine derivative and its *N*‐methylated product as the reaction progressed (Section S11 in the SI).

The through‐space substituent effects on the chemical reactivity of compound series **2‐X** mirrored several important trends observed in the molecular balance series **1‐X**:

- The experimentally determined rate constants correlate with electrostatic potentials as the X‐substituent was varied (Figure 6 A, R^2^=0.85, Tables S14, S15). The most reactive control compound was **2‐OMe** (50 % completion in ≈35 minutes), while **2‐CN** was the least reactive (40+hours to 50 % completion).
- The dimethoxyphenyl derivative **2‐a** (red, Figure 6 A), which positions two OMe groups over the pyridine ring reacted with a similar rate to the most reactive control compound **2‐OMe** (4‐methoxypyridine).
- The *ortho*‐nitrophenyl compound **2‐f** (blue, Figure 6 A), which positions a nitro group in an electro‐enhancing position over the pyridine ring attained 50 % completion in 3.5 hours vs. 5.5 hours for the electro‐attenuating *para*‐ and *meta*‐substituted nitrophenyl compounds **2‐h** and **2‐j**.
- The electronically neutral dimethyl‐ and diethylphenyl derivatives (**2‐m** and **2‐l**) exhibited similar reactivity to pyridine (**2‐H**, 50 % completion in 2 hours).
- The dinitrophenyl derivative **2‐e** drops below the line of best fit (purple in Figure 6 A *cf*. **1‐e** in Figure 4 C). This effect presumably arises in both systems due to solvation of the polar aromatic edge with an electro‐enhancing H‐bond acceptor solvent.

These common patterns are consistent with some transferability of through‐space substituent effects upon varying the X‐substituent. Supporting this assertion, the calculated electrostatic potentials taken over the *ipso*‐carbon in molecular balances (ESP_ipso_) was found to correlate strongly with electrostatic potential of the pyridine nitrogen atom (ESP_N_) (Figure 6 B, R^2^=0.97). Despite the numerous commonalities outlined above, the −log_10_(*K*_X_/*K*_H_) values determined using molecular balances correlated surprisingly poorly with the −log_10_(*k*_X_/*k*_H_) pyridine rate data when both sets of data were determined in [D_6_]acetone (R^2^=0.66, Figure 6 C). This suggests that energetic influence of the solvent on conformational preferences differs from those encountered in the transition state of the *N*‐methylation reaction. However, conformational −log_10_(*K*_X_/*K*_H_) values determined in six of the eleven solvents examined were found to correlate more strongly with −log_10_(*k*_X_/*k*_H_) (R^2^=0.88 to 0.94, Figures 6 D, S80 to S81) than the electrostatic potential of the pyridine nitrogen calculated in the gas phase (R^2^=0.85, Figure 6 A). Hence, given that the influence of the solvent on through‐space substituent effects is both complex and computationally challenging, the empirically determined conformational −log_10_(*K*_X_/*K*_H_) values compiled in Table 1 may prove to be useful in broader chemical contexts.

## Conclusion

In conclusion, we have used synthetic molecular balances and a simple model reaction to quantify the importance of through‐space substituent effects on non‐covalent interactions and reaction kinetics, respectively (Figure 1). Experimentally determined conformational equilibrium constants measured in the molecular balances were transposed onto Hammett's well‐known substituent constant scale (Table 1, Figure 2). The determined Hammett constants bring to our attention both the magnitude of the field effects, and the inadequacy of describing substituent effects in terms of “electron donation” and “electron withdrawal”. For example, a 2,6‐nitrophenyl group that positions two nitro groups over a biaryl bond were found to have a net electro‐enhancing influence comparable to that of a directly bonded OMe substituent, and contrasting with the classically accepted electron‐withdrawing nature of nitro groups (Figure 3 B). A more extreme manifestation of through‐space effects was observed with the 2,6‐dimethoxylphenyl substituent, which was found to be ≈28 % more electro‐enhancing than a directly bonded NEt_2_ group (Figure 3 C, left). Remarkably, it was possible to completely switch off the electro‐enhancing behavior via a sterically induced change of the oxygen lone pair orientations (Figure 3 C, middle). Meanwhile, OH protons pointed over the biaryl bond was found to exert a strong electro‐attenuating influence comparable to a CF_3_ group (Figure 3 C, right). The switchable nature of these substituent effects indicates these remarkable influences are manifested through space (i.e. via electric fields) and not by through‐bond electron donation or withdrawal. A total of 267 substituent/solvent combinations were determined for 25 molecular balances in eleven different solvents to examine the context dependency of through‐space substituent effects (Table 1). As anticipated, polar solvent were found to attenuate the magnitude of substituent effects projected through space (Figures 4 and 5). Nonetheless, the same substituent effects that governed the conformations of the molecular balances were also found to govern the *N*‐methylation kinetics of correspondingly substituted pyridine derivatives (Figure 6). Even though the substituents examined were all neutral and uncharged, the through‐space kinetic influences were still large enough to be manifested in the polar solvent acetone. Conformational equilibrium constants measured in several solvents correlated better with the experimental rate data than electrostatic potentials calculated in the gas phase. This suggests that the data compiled in Table 1 may prove useful for rationalizing through‐space substituent effects in other contexts and chemical reactions. Similarly, the simplicity of the model system combined with the high energetic precision of the conformational equilibrium constants measured across a range of solvents (equivalent of ±0.2 kJ mol^−1^) may also prove useful for benchmarking emerging theoretical solvation models.

## Conflict of interest

The authors declare no conflict of interest.

## Supporting information

As a service to our authors and readers, this journal provides supporting information supplied by the authors. Such materials are peer reviewed and may be re‐organized for online delivery, but are not copy‐edited or typeset. Technical support issues arising from supporting information (other than missing files) should be addressed to the authors.

Supplementary
