# Supplementary material for: Quantifying Through‐Space Substituent Effects
Source: Angew Chem Int Ed Engl. 2020 Jul 17;59(38):16717–24. doi: 10.1002/anie.202006943 (PMC7540488; doi:10.1002/anie.202006943)
Supplement: Supplementary file 1 — Supplementary [file ANIE-59-16717-s001.pdf]

Angewandte Chemie
Eine Zeitschrift der Gesellschaft Deutscher Chemiker

Supporting Information

Quantifying Through-Space Substituent Effects

*Rebecca J. Burns, Ioulia K. Mati, Kamila B. Muchowska, Catherine Adam, and Scott L. Cockroft**

anie_202006943_sm_miscellaneous_information.pdf

Contents:

Section A: Molecular balance data

S1. Computational Methods and Data……………………………………………………………...………1

S2. Conformer Assignment by NMR…………………………………………………………………...13

S3. Determination of K_X and Error Analysis……………………………………………………….……..18

S4. Determination of $-\log_{10}(K_X/K_H)$ and $\sigma_{p(conf)}$…………………….…………………………………..21

S5. NMR Chemical Shift Data for Central Ring Protons…………………………………………………24

S6. Determination of ΔG_{exp} and Error Analysis……………………………………………………...…26

S7. Linear Regression to Obtain Solvent-Independent Conformational Free Energies (ΔE)……………..33

S8. Synthetic Procedures and Standard Characterization Data……………………………………………40

S9. References for Section A…………………………………………………………………………...62

Section B: Pyridine-derivative data

S10. Computational Methods and Data……………………………………………………...………63

S11. Determination of Rate Constants (k_X) and Error Analysis ……………..77

S12. Synthetic Procedures and Standard Characterization Data ……………………………….…………85

S13. References for Section B…………………….……………………………………………………….99

Section A: Molecular balance data

S1. Computational methods and data

Minimized geometries and electrostatic potentials of the proton-capped X-substituted ring of the molecular balances (Figure S1A) were calculated at DFT/B3LYP/6–31G* using Spartan '14. (Figures S2 to S25). ESP_{ipso} values were measured on the ESP surface over both *ipso* positions relative to the formamide group on each face of the molecule (Figure S1, Table S1).

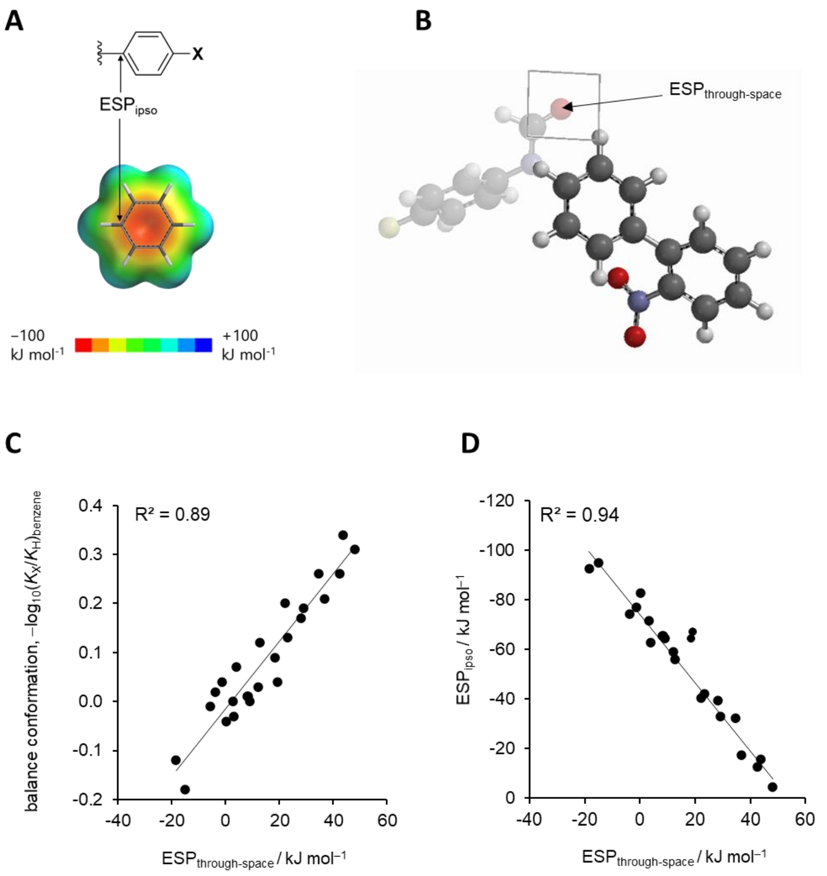

Figure S1. A) DFT/B3LYP/6–31G* Electrostatic Potential (ESP) surface used to model the electrostatic properties of **1–H**. The ESP values over the *ipso* position were taken at the 0.002 electron/Bohr^3 isosurface as indicated. All surfaces were scaled from –100 (red) to +100 kJ mol^{-1} (blue). B) Position at which ES-$P_{through-space}$ was determined by overlaying the biphenyl fragment on the full minimized balance structure. C) and D) Correlations of $ESP_{through-space}$ with experimental conformational equilibrium constants and ESP_{ipso}, respectively.

Through-space ESPs ($ESP_{through\text{-}space}$) were performed using electrostatic potential slices that were calculated for each X substituent fragment and overlaid on the previously minimized geometries of the corresponding full balance (Figure S1B). The $ESP_{through\text{-}space}$ was then taken on the slice of the X substituent fragment at the atom-centre position occupied by the carbonyl oxygen in the minimized 'O' conformer of the balance in full balance (Table S1). This ensured that only the influence of the X substituents electrostatic potential propagating through space was taken into account. Excellent correlations were seen for both the experimental balance conformation measured in benzene (Figure S1C) and ESP_{ipso} (Figure S1D) vs $ESP_{through\text{-}space}$ as the X-substituent was varied.

Table S1. Mean ESP_{ipso} values of all phenyl fragments in the **1–X** series of balances with the two ESP_{ipso} values recorded on each face of the aromatic ring, as well as $ESP_{through\text{-}space}$ values recorded at the position occupied by the formyl oxygen atom in the O conformer. All structures and surfaces (from which ESP_{ipso} values were determined) were minimized and calculated using DFT/B3LYP/6–31G* in Spartan '14. All values in kJ mol^{-1}.

Compound	ESP_{ipso} (1)	ESP_{ipso} (2)	Mean ESP_{ipso}	$ESP_{through\text{-}space}$
**1–H**	–69.7	–71.1	–70.4	2.8
**1–OMe**	–82.0	–81.6	–81.8	–5.8
**1–NEt_2**	–89.6	–95.3	–92.5	–18.5
**1–Me**	–76.7	–77.0	–76.9	–1.4
**1–Ph**	–66.7	–66.9	–66.8	19.2
**1–F**	–55.8	–55.8	–55.8	12.6
**1–CN**	–12.8	–12.2	–12.5	42.5
**1–NO_2**	–4.8	–4.0	–4.4	48.0
**1–CF_3**	–32.7	–33.1	–32.9	29.0
**1–$COCH_3$**	–38.8	–39.7	–39.3	28.1
**1–Br**	–42.9	–41.5	–42.2	23.2
**1–a**	–95.7	–94.3	–95.0	–15.0
**1–b**	–63.3	–62.3	–62.8	3.9
**1–c**	–17.9	–16.4	–17.2	36.7
**1–d**	–66.0	–65.2	–65.6	8.4
**1–e**	–15.6	–15.5	–15.6	43.6
**1–f**	–49.8	–68.1	–59.0	12.1

**1–g**	–82.6	–82.7	–82.7	0.1
**1–h**	–33.4	–31.2	–32.3	34.7
**1–i**	–73.6	–75.2	–74.4	–3.8
**1–j**	–40.9	–39.5	–40.2	22.0
**1–k**	–74.8	–68.6	–71.7	3.1
**1–l**	–66.1	–62.5	–64.3	9.0
**1–m**	–65.1	–65.8	–65.5	8.1
**1–n**	–69.9	–58.3	–64.1	18.5

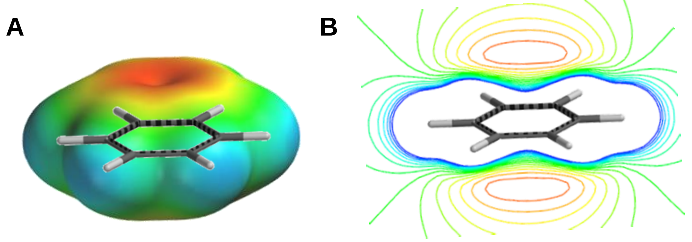

Figure S2. ESP surface (**A**) and slice (**B**) of **1–H**. Calculated using DFT/B3LYP/6–31G*. Scaled from –100 kJ mol^{-1} (red) to +100 kJ mol^{-1} (blue) on the 0.002 electron/Bohr^3 isosurface.

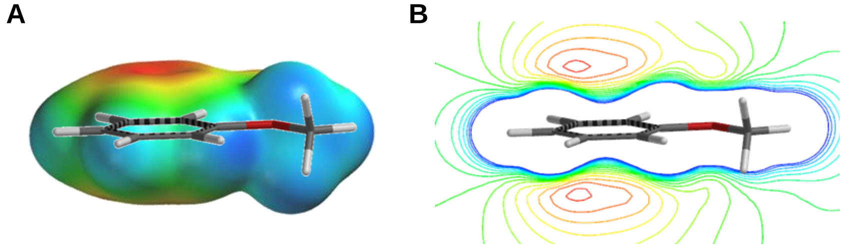

Figure S3. ESP surface (**A**) and slice (**B**) of **1–OMe**. Calculated using DFT/B3LYP/6–31G*. Scaled from –100 kJ mol^{-1} (red) to +100 kJ mol^{-1} (blue) on the 0.002 electron/Bohr^3 isosurface.

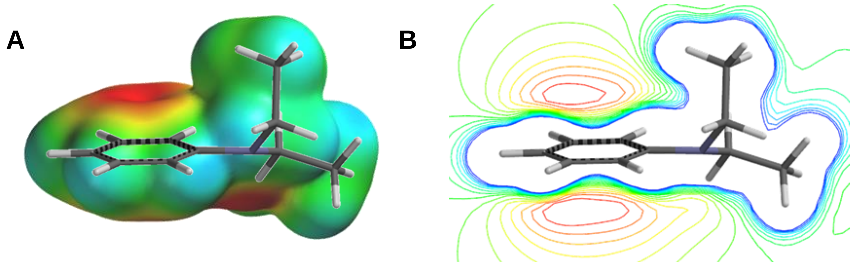

Figure S4. ESP surface (**A**) and slice (**B**) of **1–NEt$_2$.** Calculated using DFT/B3LYP/6–31G*. Scaled from –100 kJ mol^{-1} (red) to +100 kJ mol^{-1} (blue) on the 0.002 electron/Bohr^3 isosurface.

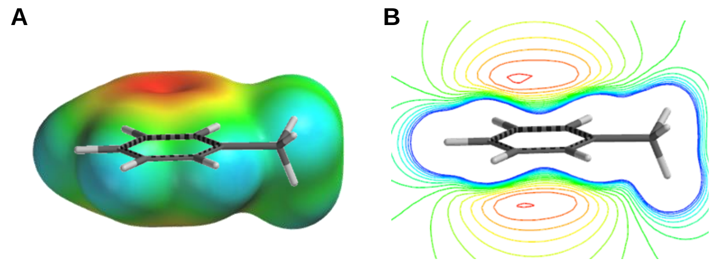

Figure S5. ESP surface (**A**) and slice (**B**) of **1–Me**. Calculated using DFT/B3LYP/6–31G*. Scaled from –100 kJ mol^{-1} (red) to +100 kJ mol^{-1} (blue) on the 0.002 electron/Bohr^3 isosurface.

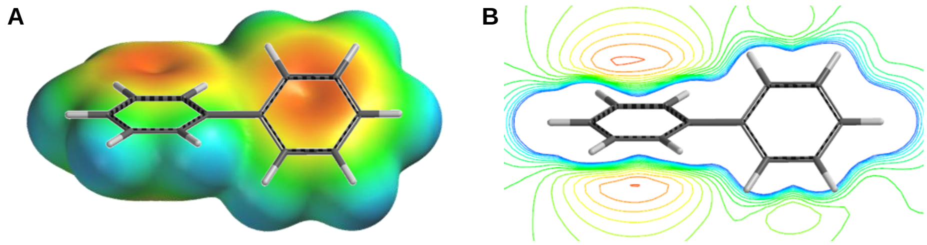

Figure S6. ESP surface (**A**) and slice (**B**) of **1–Ph**. Calculated using DFT/B3LYP/6–31G*. Scaled from –100 kJ mol^{-1} (red) to +100 kJ mol^{-1} (blue) on the 0.002 electron/Bohr^3 isosurface.

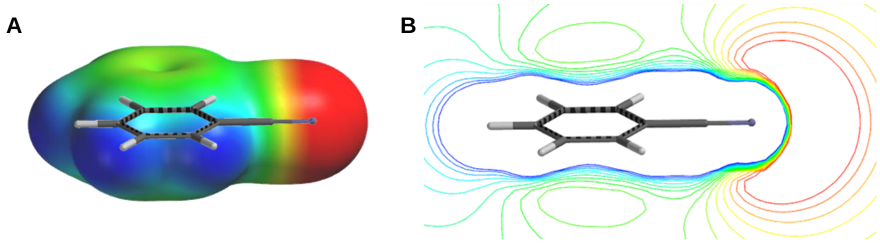

Figure S7. ESP surface (**A**) and slice (**B**) of **1–CN**. Calculated using DFT/B3LYP/6–31G*. Scaled from –100 kJ mol^{-1} (red) to +100 kJ mol^{-1} (blue) on the 0.002 electron/Bohr^3 isosurface.

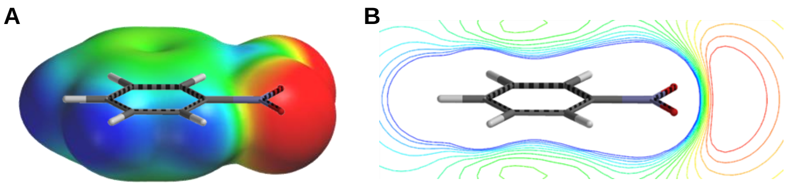

Figure S8. ESP surface (**A**) and slice (**B**) of **1–NO$_2$**. Calculated using DFT/B3LYP/6–31G*. Scaled from –100 kJ mol^{-1} (red) to +100 kJ mol^{-1} (blue) on the 0.002 electron/Bohr^3 isosurface.

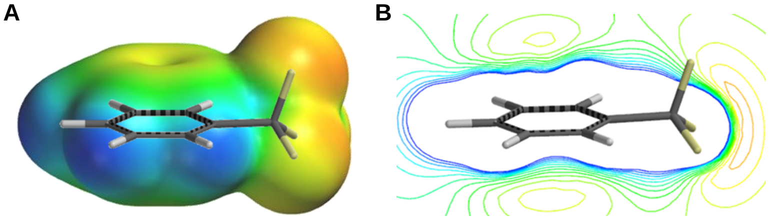

Figure S9. ESP surface (**A**) and slice (**B**) of **1–CF$_3$**. Calculated using DFT/B3LYP/6–31G*. Scaled from –100 kJ mol^{-1} (red) to +100 kJ mol^{-1} (blue) on the 0.002 electron/Bohr^3 isosurface.

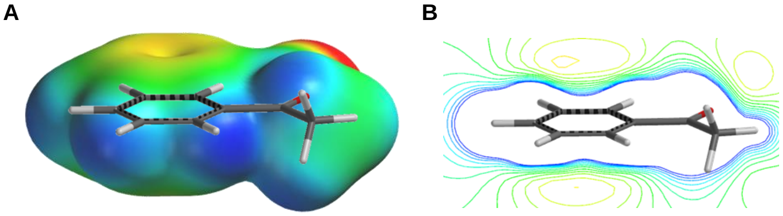

Figure S10. ESP surface (**A**) and slice (**B**) of **1–COCH$_3$**. Calculated using DFT/B3LYP/6–31G*. Scaled from –100 kJ mol^{-1} (red) to +100 kJ mol^{-1} (blue) on the 0.002 electron/Bohr^3 isosurface.

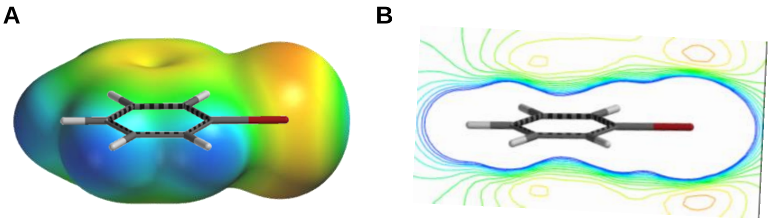

Figure S11. ESP surface (**A**) and slice (**B**) of **1–Br**. Calculated using DFT/B3LYP/6–31G*. Scaled from –100 kJ mol^{-1} (red) to +100 kJ mol^{-1} (blue) on the 0.002 electron/Bohr^3 isosurface.

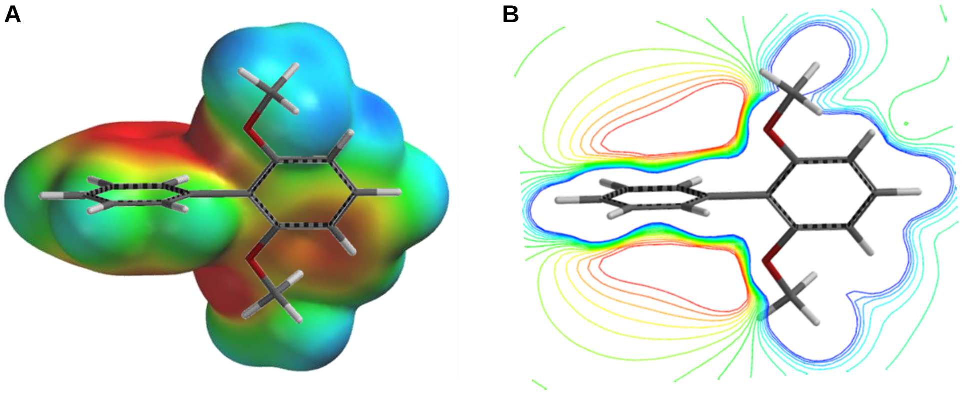

Figure S12. ESP surface (**A**) and slice (**B**) of **1**–**a**. Calculated using DFT/B3LYP/6–31G*. Scaled from –100 kJ mol^{-1} (red) to +100 kJ mol^{-1} (blue) on the 0.002 electron/Bohr^3 isosurface.

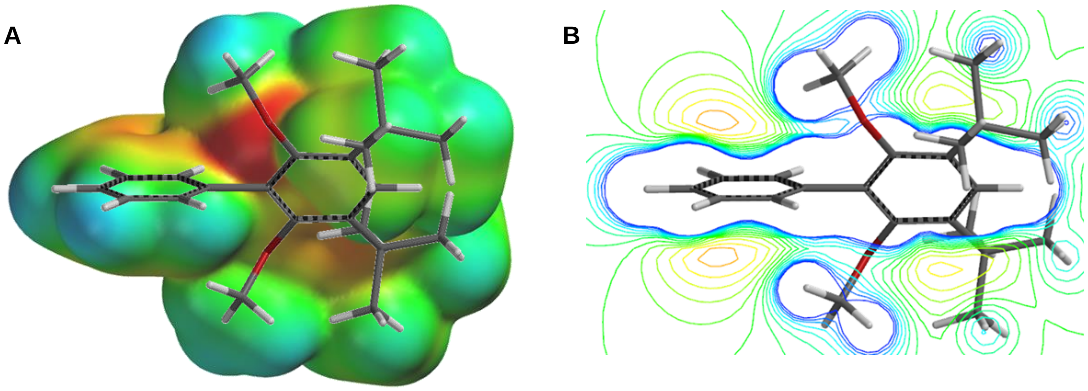

Figure S13. ESP surface (**A**) and slice (**B**) of **1**–**b**. Calculated using DFT/B3LYP/6–31G*. Scaled from –100 kJ mol^{-1} (red) to +100 kJ mol^{-1} (blue) on the 0.002 electron/Bohr^3 isosurface.

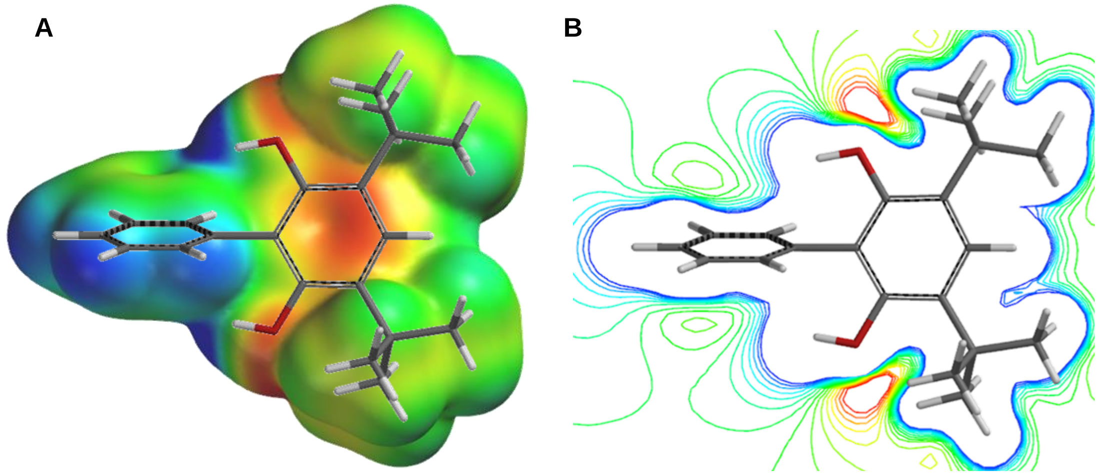

Figure S14. ESP surface (**A**) and slice (**B**) of **1**–**c**. Calculated using DFT/B3LYP/6–31G*. Scaled from –100 kJ mol^{-1} (red) to +100 kJ mol^{-1} (blue) on the 0.002 electron/Bohr^3 isosurface.

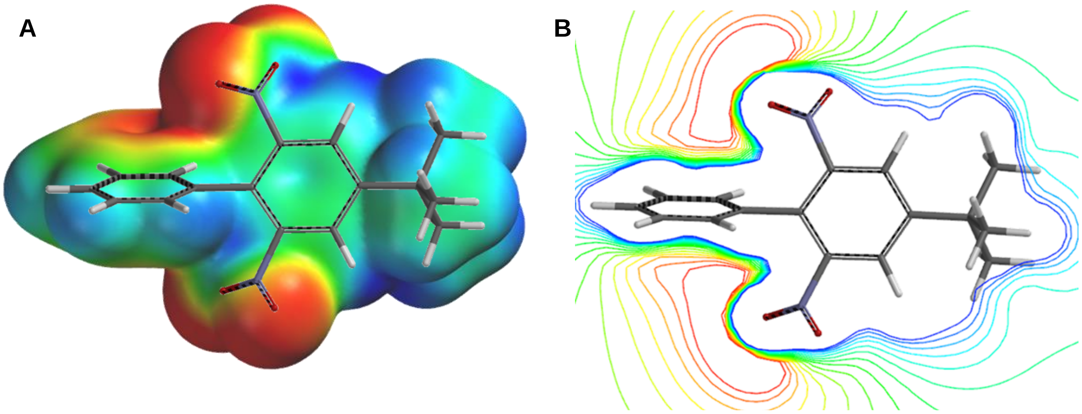

Figure S15. ESP surface (**A**) and slice (**B**) of **1**–**d**. Calculated using DFT/B3LYP/6–31G*. Scaled from –100 kJ mol^{-1} (red) to +100 kJ mol^{-1} (blue) on the 0.002 electron/Bohr^3 isosurface.

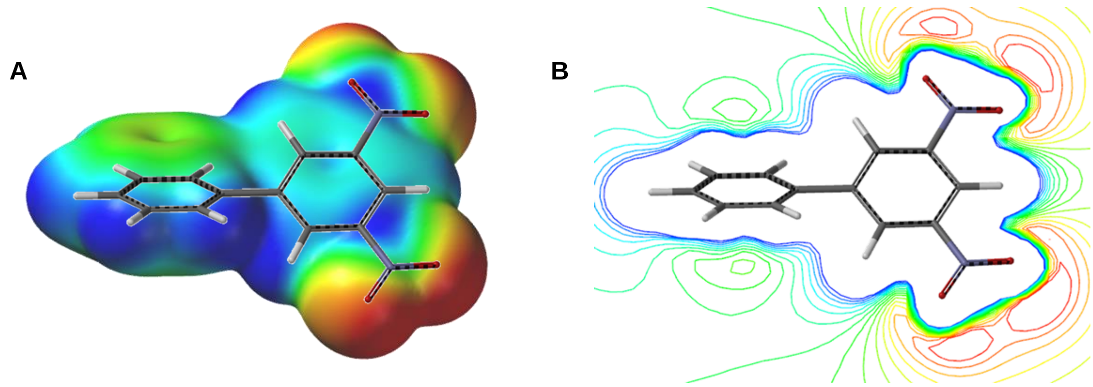

Figure S16. ESP surface (**A**) and slice (**B**) of **1–e**. Calculated using DFT/B3LYP/6–31G*. Scaled from –100 kJ mol^{-1} (red) to +100 kJ mol^{-1} (blue) on the 0.002 electron/$Bohr^3$ isosurface.

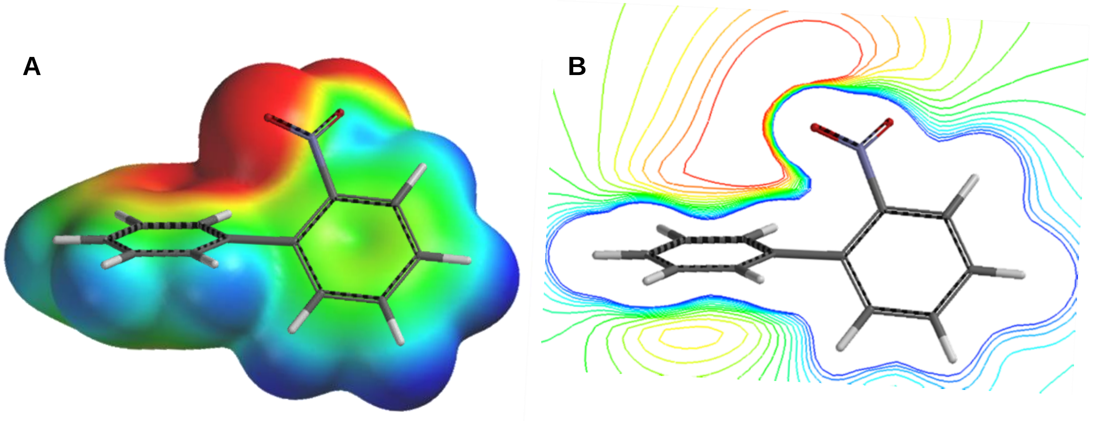

Figure S17. ESP surface (**A**) and slice (**B**) of **1–f**. Calculated using DFT/B3LYP/6–31G*. Scaled from –100 kJ mol^{-1} (red) to +100 kJ mol^{-1} (blue) on the 0.002 electron/$Bohr^3$ isosurface.

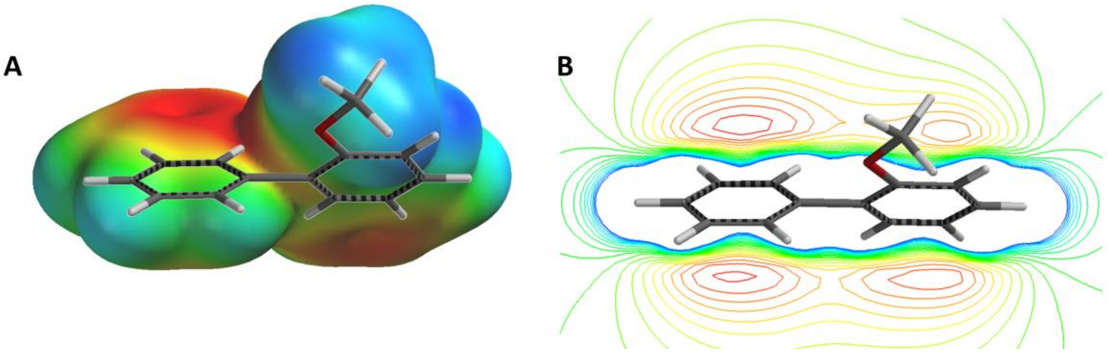

Figure S18. ESP surface (**A**) and slice (**B**) of **1–g**. Calculated using DFT/B3LYP/6–31G*. Scaled from –100 kJ mol^{-1} (red) to +100 kJ mol^{-1} (blue) on the 0.002 electron/$Bohr^3$ isosurface.

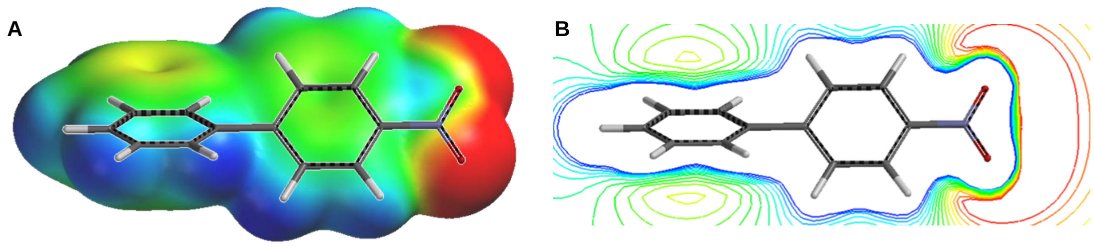

Figure S19. ESP surface (**A**) and slice (**B**) of **1–h**. Calculated using DFT/B3LYP/6–31G*. Scaled from –100 kJ mol^{-1} (red) to +100 kJ mol^{-1} (blue) on the 0.002 electron/Bohr^3 isosurface.

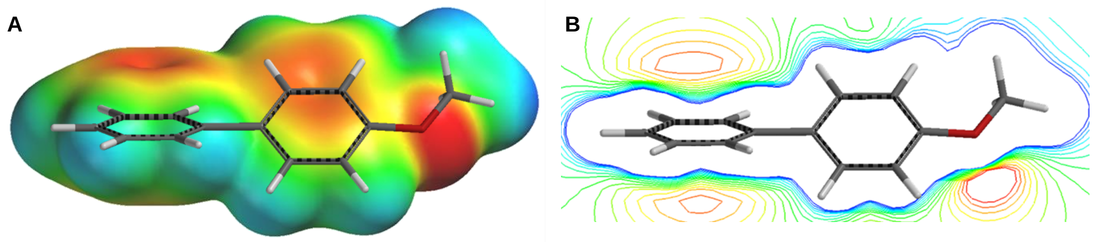

Figure S20. ESP surface (**A**) and slice (**B**) of **1–i**. Calculated using DFT/B3LYP/6–31G*. Scaled from –100 kJ mol^{-1} (red) to +100 kJ mol^{-1} (blue) on the 0.002 electron/Bohr^3 isosurface.

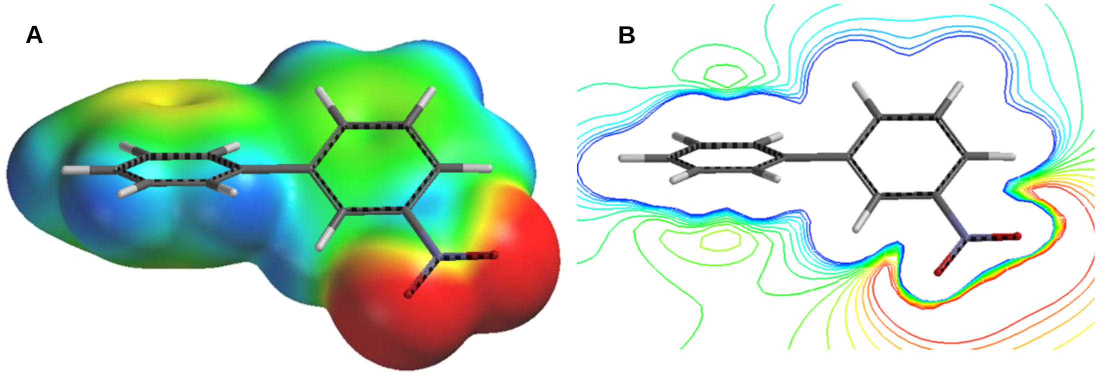

Figure S21. ESP surface (**A**) and slice (**B**) of **1–j**. Calculated using DFT/B3LYP/6–31G*. Scaled from –100 kJ mol^{-1} (red) to +100 kJ mol^{-1} (blue) on the 0.002 electron/Bohr^3 isosurface.

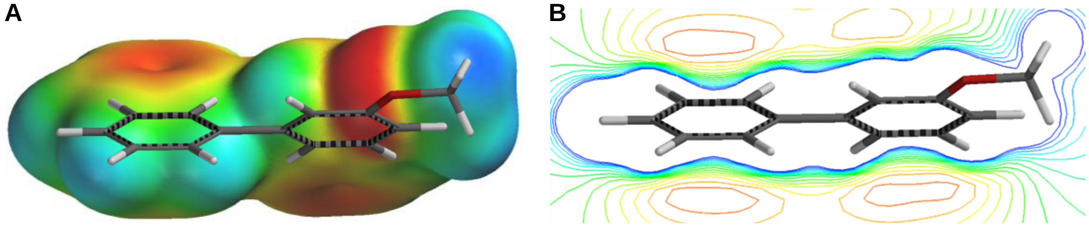

Figure S22. ESP surface (**A**) and slice (**B**) of **1–k**. Calculated using DFT/B3LYP/6–31G*. Scaled from –100 kJ mol^{-1} (red) to +100 kJ mol^{-1} (blue) on the 0.002 electron/Bohr^3 isosurface.

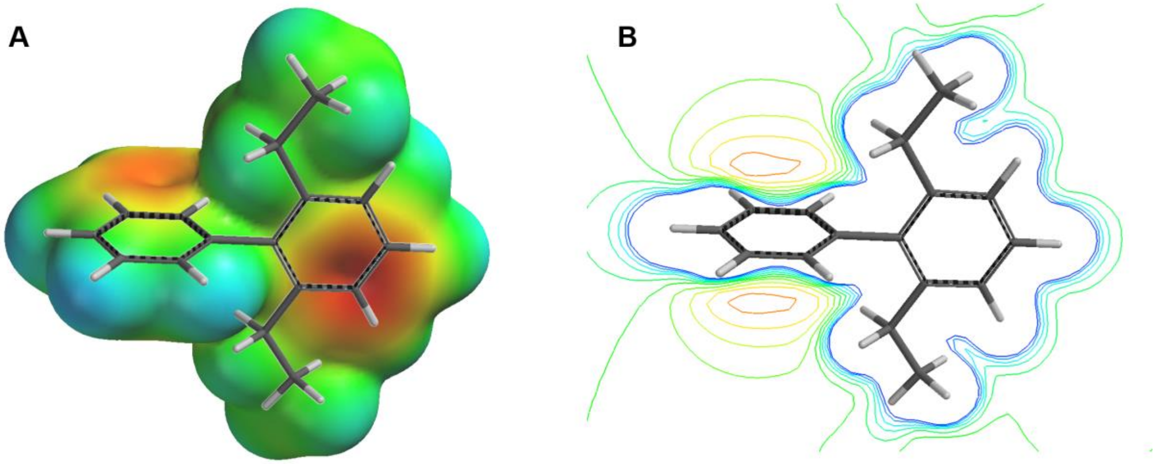

Figure S23. ESP surface (**A**) and slice (**B**) of **1–l**. Calculated using DFT/B3LYP/6–31G*. Scaled from –100 kJ mol^{-1} (red) to +100 kJ mol^{-1} (blue) on the 0.002 electron/Bohr^3 isosurface.

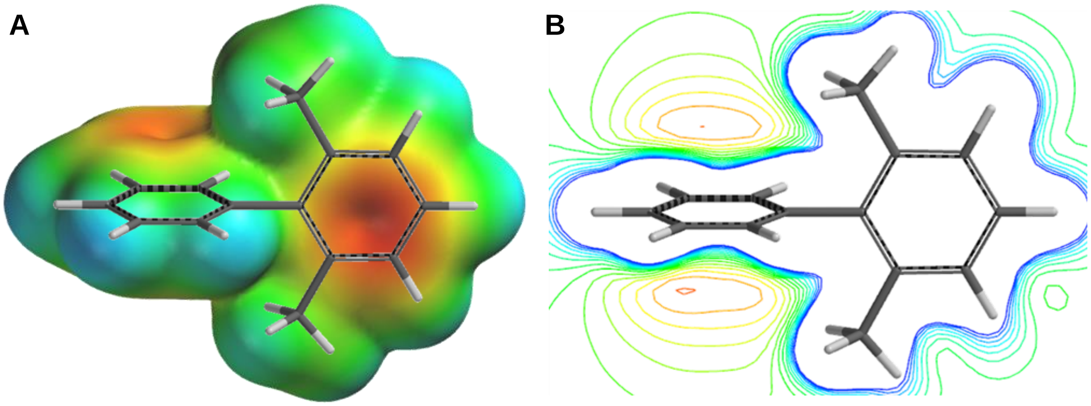

Figure S24. ESP surface (**A**) and slice (**B**) of **1–m**. Calculated using DFT/B3LYP/6–31G*. Scaled from –100 kJ mol^{-1} (red) to +100 kJ mol^{-1} (blue) on the 0.002 electron/Bohr^3 isosurface.

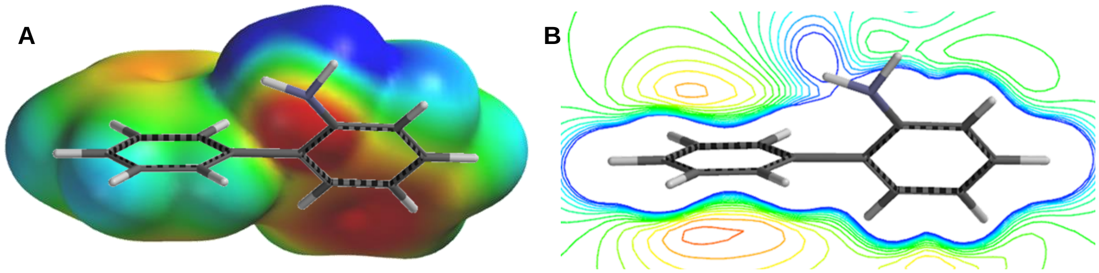

Figure S25. ESP surface (**A**) and slice (**B**) of **1–n**. Calculated using DFT/B3LYP/6–31G*. Scaled from –100 kJ mol^{-1} (red) to +100 kJ mol^{-1} (blue) on the 0.002 electron/$Bohr^3$ isosurface.

S2. Conformer Assignment by NMR

All molecular torsion balances were fully characterized by NMR in $CDCl_3$ to assign conformer peaks of all balances. Figures S26 to S34 show the NMR spectra (^{1}H, ^{13}C, HSQC, COSY, HMBC and NOESY) of **1–Me** in $CDCl_3$ together with the full assignment of both conformers (Figure S26). The 2D and 1D spectra were used to assign the conformers of **1–Me** as shown below. In this example, proton resonances have been labelled numerically and carbon resonances alphabetically. The minor conformer peaks have been denoted with a prime (′).

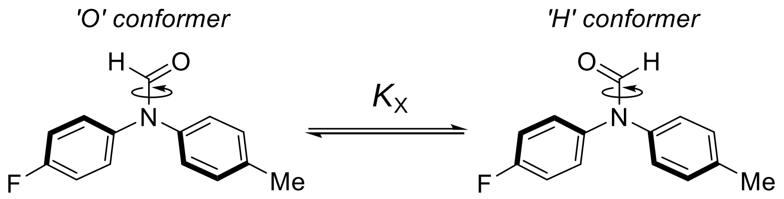

Figure S26. Conformational equilibrium of **1–Me** between the 'O' and 'H' conformers.

The major conformer in the ^{19}F spectra were assumed to be the same as that in $CDCl_3$. The ^{19}F chemical shift difference of the two conformers was plotted across the range of solvents studied for all balances to identify outliers where the dominant conformer switched as the solvent in different solvents. Full NMR analysis was performed on any outliers identified. For those solvents where a deuterated sample was not available, the major conformer in the ^{19}F spectra were assumed to be the same as that in $CDCl_3$ for DCM outliers, and methanol for ethanol outliers.

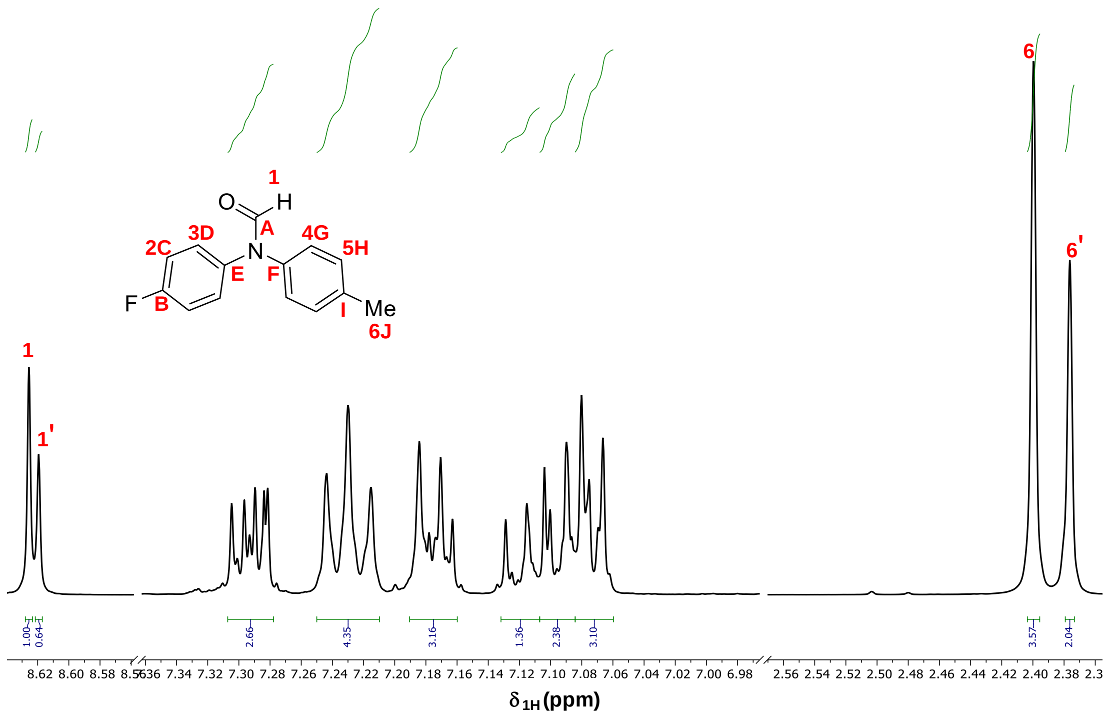

Figure S27. ^1H NMR of **1–Me** in $CDCl_3$ (500.1 MHz) at 298 K.

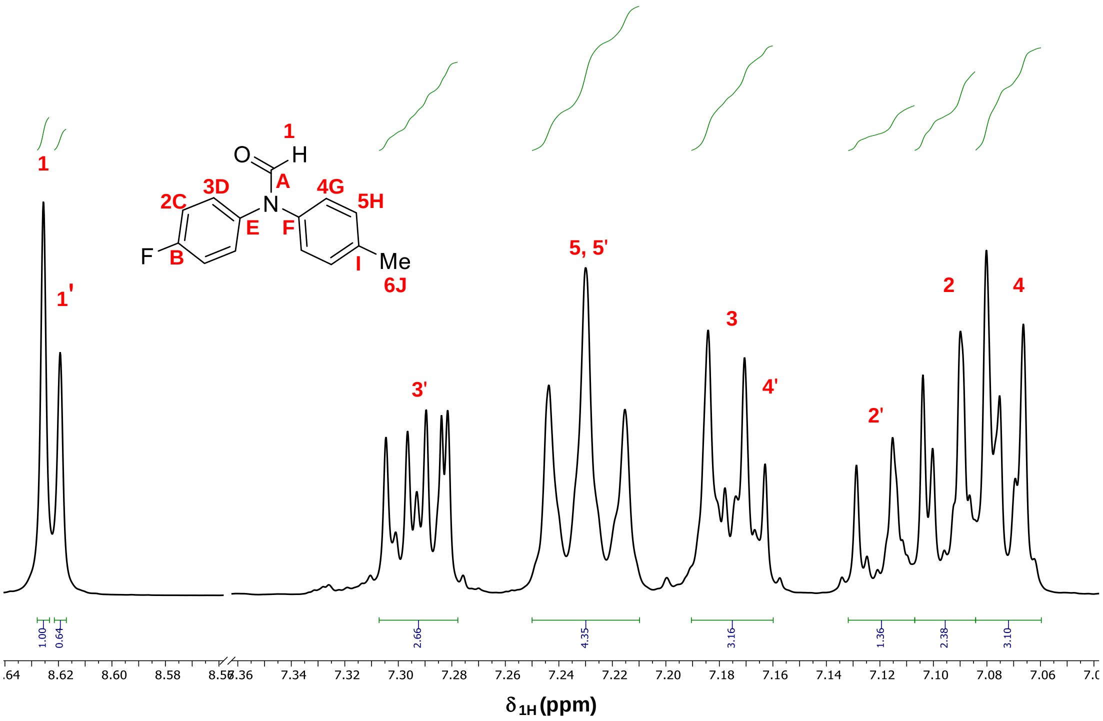

Figure S28. ^1H NMR of **1–Me** in $CDCl_3$ at (500.1 MHz) 298 K, zoomed into aromatic region.

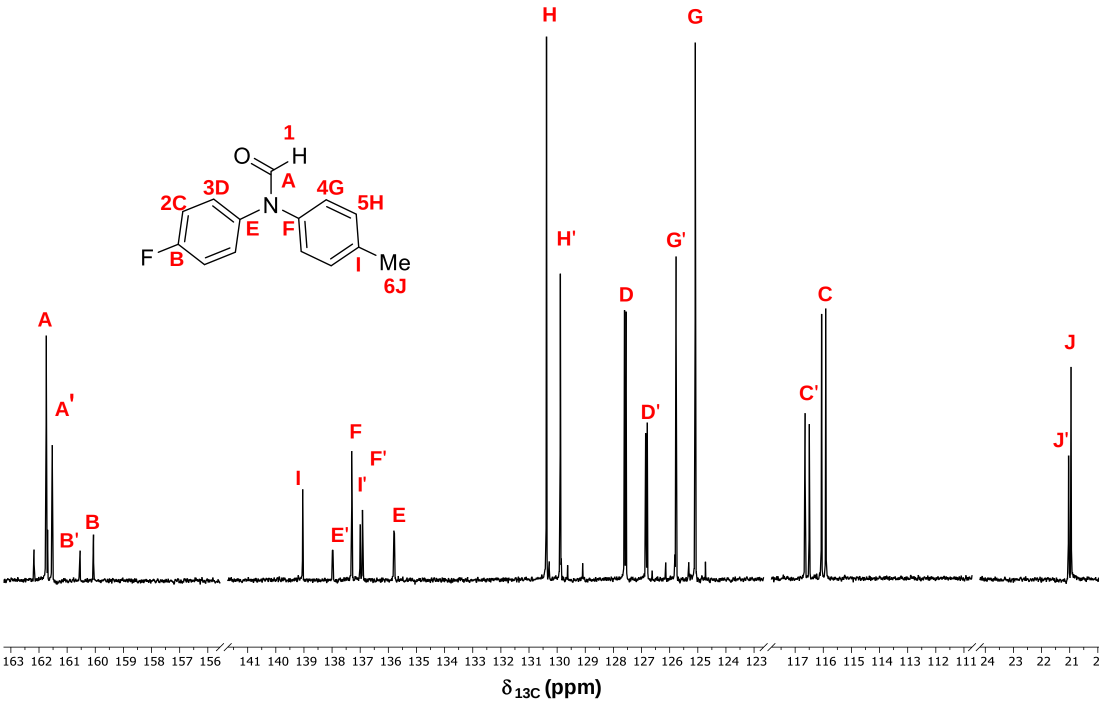

Figure S29. ^{13}C NMR of **1–Me** in $CDCl_3$ (126 MHz) at 298 K.

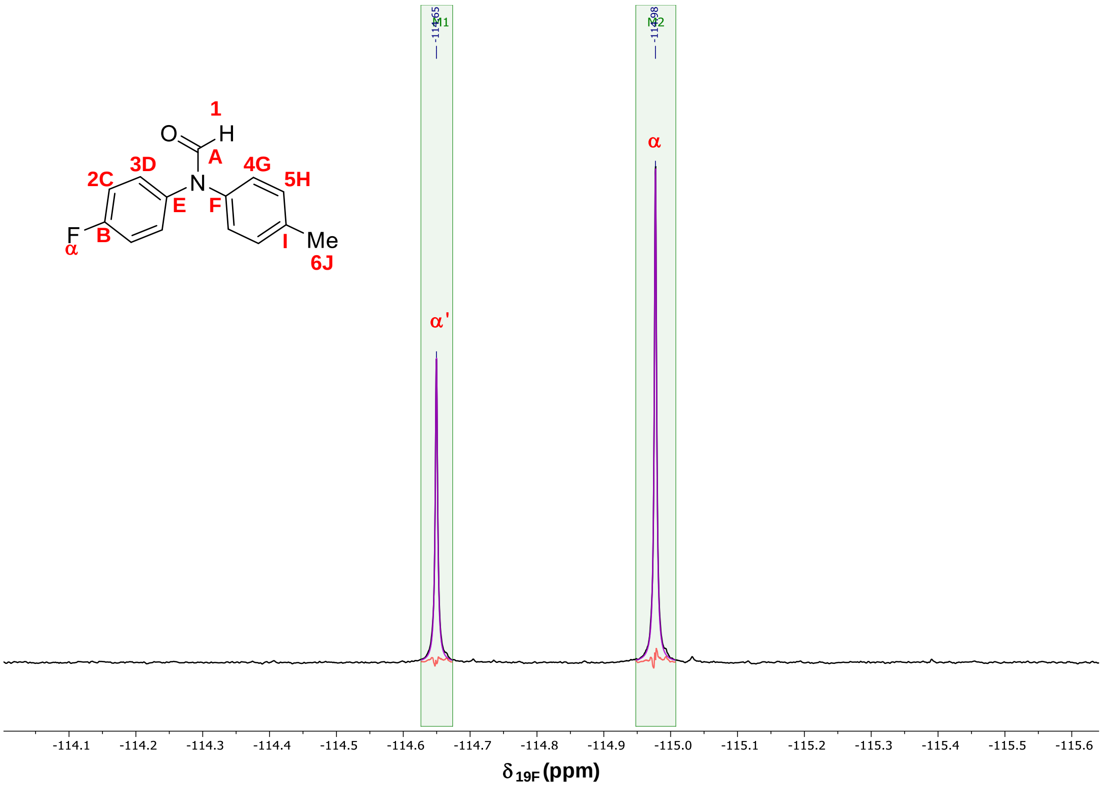

Figure S30. Proton decoupled ^{19}F NMR of **1–Me** in (376.5 MHz) $CDCl_3$ at 298 K.

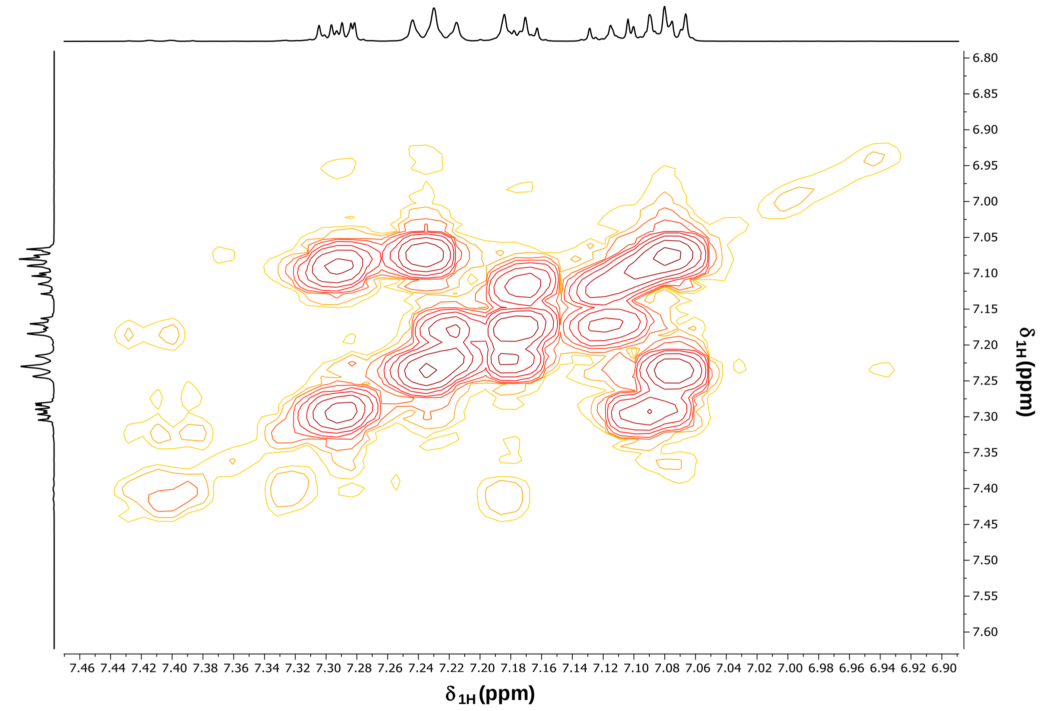

Figure S31. COSY of **1–Me** in $CDCl_3$ at (500.1 MHz) 298 K, zoomed into the aromatic region.

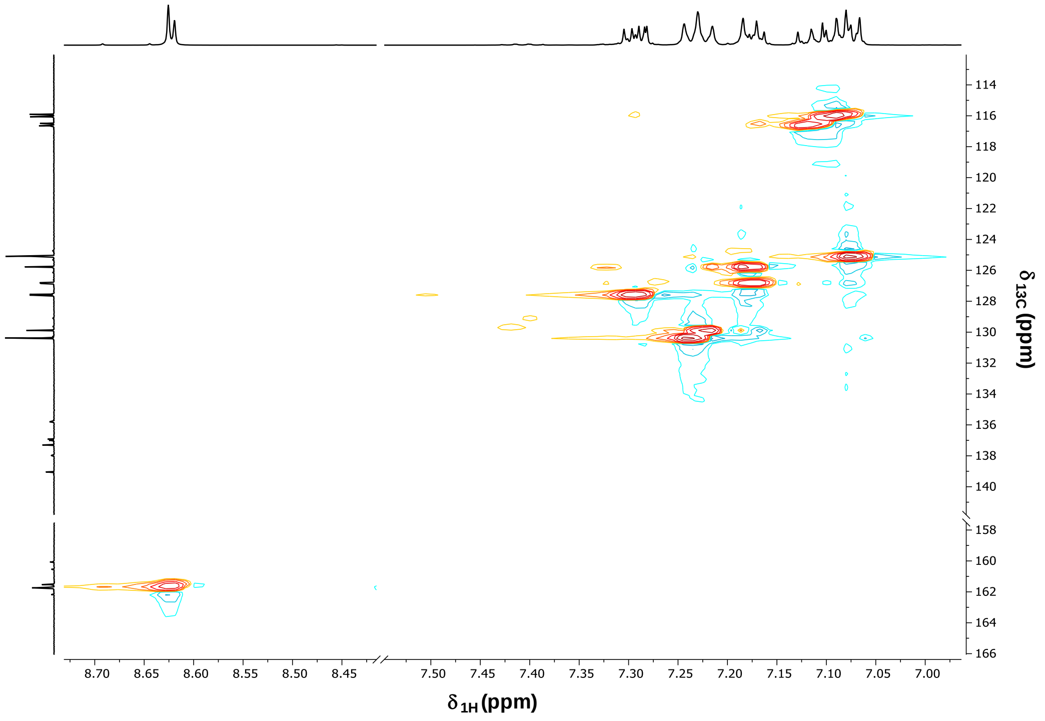

Figure S32. HSQC of **1–Me** in $CDCl_3$ (500.1/126 MHz) at 298 K.

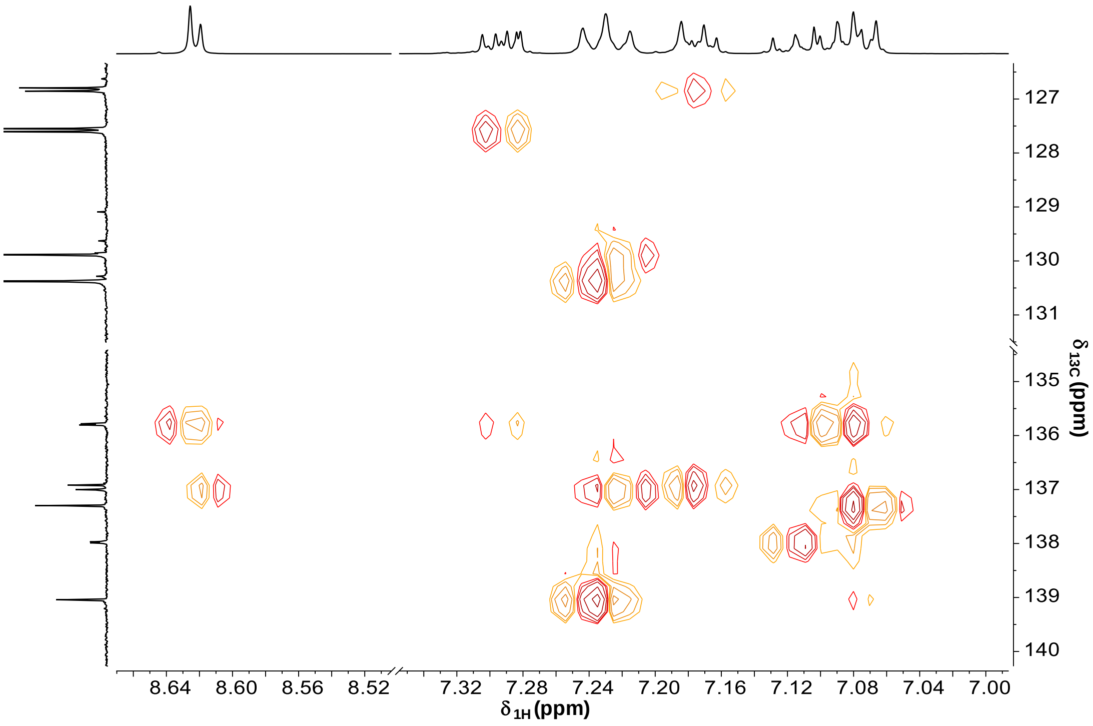

Figure S33. HMBC of **1–Me** in $CDCl_3$ (500.1/126 MHz) at 298 K.

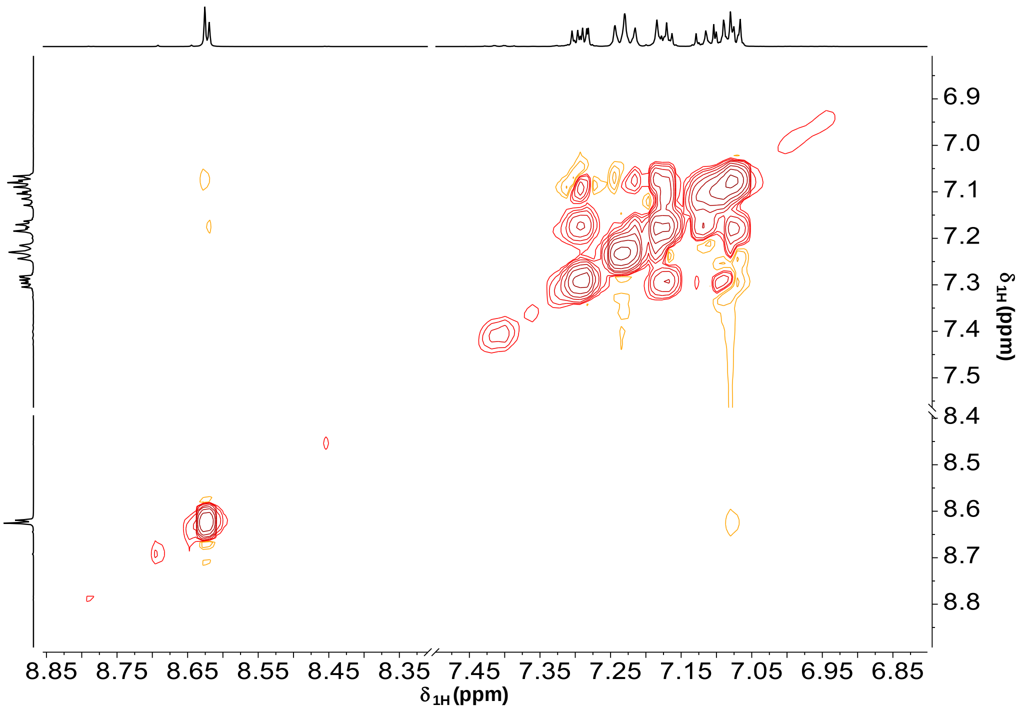

Figure S34. NOESY of **1–Me** in $CDCl_3$ (500.1 MHz) at 298 K.

S3. Determination of K_X and error analysis

All molecular balances were fully characterized and the ‘O’ and ‘H’ conformers (Figure S35) were assigned in chloroform-*d* *via* 2D NMR analysis (detailed in Section S2). Quantitative analysis was performed using proton decoupled ^{19}F NMR spectroscopy ($^{19}F\{^1H\}$ NMR) recorded using a Bruker Ultrashield 400 MHz, heteronuclear (128 scans). Integrals of the $^{19}F\{^1H\}$ peaks were obtained through manual line fitting *via* MestreNova following manual phase correction and Whittaker Smoother baseline correction. The conformational equilibrium constant, K_X, was determined using the ratios of these peak integrals in benzene-d_6 at 298 K *via* Equation S1.

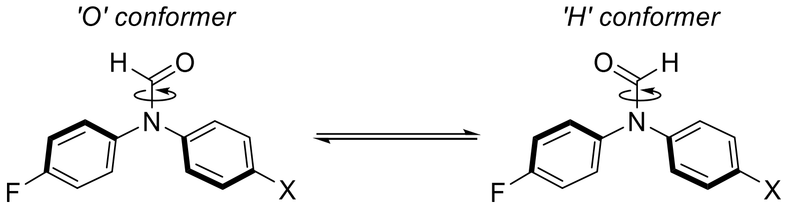

Figure S35. Definition of the equilibrium between the ‘O’ conformer and the ‘H’ conformers of the molecular balance studies. The relationship of each conformer with the equilibrium constant, K_X, is shown.

$$K_X = \frac{[H\ conformer]}{[O\ conformer]} \qquad \textbf{Equation S1}$$

Where “H conformer” is the ^{19}F integral of the H conformer and “O conformer” is that of the O conformer.

An error of 3% was applied to the integral of the minor NMR peak (e.g. the integration ratio was [∫major conformer = 1] / [∫minor conformer ± 0.03]). K_X values and their errors are presented in Table S2.

Conformer ratios were shown not to vary with concentration within the range used for our NMR study (Figure S36).

Table S2. Experimental equilibrium constants (K_X) measured in benzene-d_6 (376.5 MHz, 298 K).

Compound	K_X	δK_X
**1–H**	1.32	0.04
**1–OMe**	1.35	0.04
**1–NEt_2**	1.75	0.05
**1–Me**	1.20	0.04
**1–Ph**	1.20	0.04
**1–CN**	0.73	0.02
**1–NO_2**	0.65	0.02
**1–CF_3**	0.85	0.03
**1–$COCH_3$**	0.89	0.03
**1–Br**	0.97	0.03
**1–F**	1.00	*N/A*
**1–a**	1.97	0.06
**1–b**	1.13	0.03
**1–c**	0.82	0.02
**1–d**	1.28	0.04
**1–e**	0.61	0.02
**1–f**	1.23	0.02
**1–g**	1.45	0.04
**1–h**	0.73	0.02
**1–i**	1.27	0.04
**1–j**	0.82	0.02
**1–k**	1.40	0.04
**1–l**	1.33	0.04
**1–m**	1.30	0.04
**1–n**	1.08	0.03

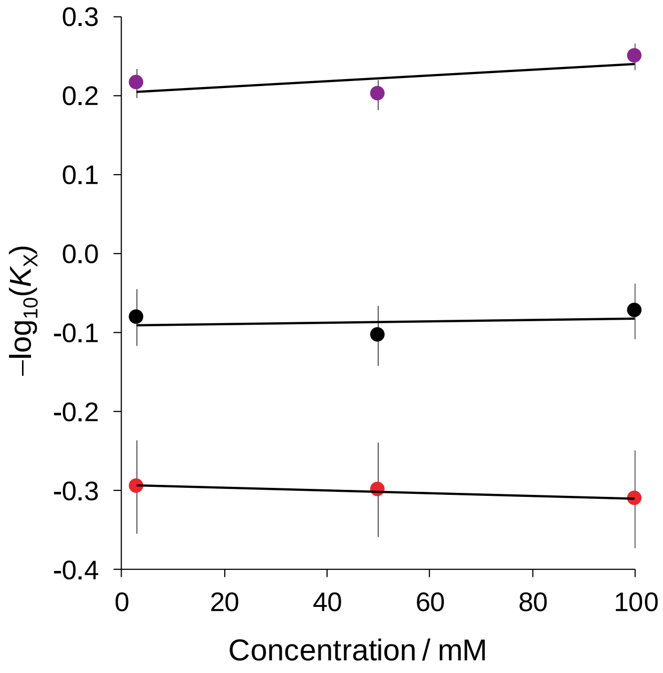

Figure S36: Concentration dependence of molecular balances **1**–**a** (red), **1**–**e** (purple) and **1**–**Ph** (black) over 100 mM range measured by $^{19}F\{^{1}H\}$ NMR spectroscopy (376.5 MHz at 298 K) in benzene-d_6 with error bars included.

S4. Determination of $-\log_{10}(K_X/K_H)$ and $\sigma_{p(conf)}$ and error analysis

Hammett-style analysis using the relationship $-\log_{10}(K_X/K_H)$ was performed on the experimental conformational free energies, K_X, measured in in benzene-d_6 at 400 MHz, 298 K (Table S3). Errors in $-\log_{10}(K_X/K_H)$, $\delta\log_{10}(K_X/K_H)$, were calculated *via* a propagation of errors from K_X values (Equation S2).

$$\delta log_{10}\left(\frac{K_X}{K_H}\right) = \sqrt{\left(\frac{\delta K_X}{K_X}\right)^2 + \left(\frac{\delta K_H}{K_H}\right)^2} \qquad \textbf{Equation S2}$$

With the conservative error in K_X set at 3% in the integral of the minor NMR peak for all compounds, the value of $\delta\log_{10}(K_X/K_H)$ was < ±0.04 for series **1–X**.

Table S3. $-\log_{10}(K_X/K_H)$ values of all molecular balances studied measured in benzene-d_6 (376.5 MHz, 298 K) and substituent constants, $\sigma_{p(conf)}$, obtained from the correlation shown in Figure 2B of the main text. The error in $-\log_{10}(K_X/K_H)$ was 0.04 and the conservative error in $\sigma_{p(conf)}$ was ±0.08 for all compounds (see above). Numbers in parentheses are the accepted Hammett σ_p values.[4-5]

Compound	$-\log_{10}(K_X/K_H)$	$\sigma_{p(conf)}$
**1–H**	0	–0.21 (0.00)
**1–OMe**	–0.01	–0.25 (–0.27)
**1–NEt$_2$**	–0.12	–0.66 (–0.72)
**1–Me**	0.04	–0.07 (–0.17)
**1–Ph**	0.04	–0.07 (+0.01)
**1–CN**	0.26	+0.72 (+0.66)
**1–NO$_2$**	0.31	+0.90 (+0.78)
**1–CF$_3$**	0.19	+0.44 (+0.54)
**1–COCH$_3$**	0.17	+0.37 (+0.50)
**1–Br**	0.13	+0.25 (+0.23)
**1–F**	0.12	+0.22 (+0.15)
**1–a**	–0.18	–0.85
**1–b**	0.07	+0.03
**1–c**	0.21	+0.54
**1–d**	0.01	–0.17
**1–e**	0.34	+1.00
**1–f**	0.03	–0.11
**1–g**	–0.04	–0.37
**1–h**	0.26	+0.72
**1–i**	0.02	–0.15
**1–j**	0.20	+0.53
**1–k**	–0.03	–0.31
**1–l**	0.00	–0.23
**1–m**	0.01	–0.19
**1–n**	0.09	+0.10

The values of $-\log_{10}(K_X/K_H)$ were transposed onto the established Hammett scale through correlation between these values of the **1–X** series (Figure 2B main text). An empirical estimate of the error in $\sigma_{p(conf)}$, $\delta\sigma_{p(conf)}$, was taken as the value by which the data point that deviates furthest from

the best fit line in Figure 2B in the main text, which was 0.06. Thus, a conservative empirical estimate of the error, $\delta\sigma_{p(conf)} < \pm 0.08$ could be assumed.

S5. NMR Chemical Shift Data for Central Ring Protons

Chemical shift data (Table S4) of the X substituted ring in the *ortho* and *meta* position to the formamide of molecular balances **1–X** (Figure S37).

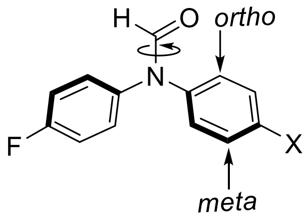

Figure S37. Position of "*ortho*" and "*meta*" referred to in Table S4 and in the accompanying discussion regarding ^{1}H NMR chemical shifts.

Table S4. ^{1}H chemical shifts of the **1–X** series in chloroform-*d* (500.1 MHz, 298 K).[2-3]

	**Chemical shift (ppm) of *ortho* proton in $CDCl_3$**		**Chemical shift (ppm) of *meta* proton in $CDCl_3$**	
**Compound**	**'H' conformer**	**'O' conformer**	**'H' conformer**	**'O' conformer**
**1–H**	7.16	7.29	7.42	7.40
**1–OMe**	7.11	7.19	6.94	6.91
**1–NEt_2**	7.01	7.05	6.66	6.64
**1–Me**	7.07	7.18	7.24	7.22
**1–Ph**	7.23	7.35	7.63	7.60
**1–CN**	7.20	7.44	7.68	7.63
**1–NO_2**	7.29	7.54	8.29	8.25
**1–CF_3**	7.24	7.46	7.70	7.66
**1–$COCH_3$**	7.22	7.44	8.02	7.99
**1–Br**	7.03	7.18	7.54	7.51
**1–a**	7.17	7.31	7.41	7.39
**1–b**	7.23	7.37	7.59	7.55
**1–c**	7.37	7.54	7.51	7.48
**1–d**	7.18	7.40	7.40	7.25
**1–e**	7.34	7.52	7.74	7.70
**1–f**	7.24	7.38	7.38	7.36
**1–g**	7.20	7.36	7.61	7.57

**1–h**	7.23	7.34	7.57	7.61
**1–i**	7.22	7.35	7.60	7.57
**1–j**	7.60	7.72	7.46	7.33
**1–k**	7.21	7.60	7.62	7.35
**1–l**	7.21	7.37	7.23	7.19
**1–m**	7.21	7.36	7.21	7.10
**1–n**	7.25	7.37	7.52	7.50

S6. Determination of ΔG_{exp} and Error Analysis

Conformational equilibrium constants, K_X, were determined in eleven solvents at 298 K using the method outlined in Section S4 (Table 1 in main text, Figures S38 and S39). Experimental conformational free energies, ΔG_{exp}, were then calculated according to Equation S3 where R is the gas constant and T is the temperature in Kelvin. The ΔG_{exp} values of all balances in the **1−X** series measured in a range of solvents are provided in Tables S5 to S8.

$$\Delta G_{exp} = -RTlnK_X \quad \textbf{Equation S3}$$

As discussed in Section S4, a conservative 3% estimate of the error in the integral of the minor conformer NMR peak was applied (e.g. the integration ratio was [∫major conformer = 1] / [∫minor conformer ± 0.03]). The error in ΔG_{exp} due to integration errors is very small when K_X lies close to 1. Thus, where the estimated error determined using the approach above was less than ±0.12 kJ mol^{-1}, a standard error of ±0.12 kJ mol^{-1} was used to accommodate potential systematic experimental errors. Indeed, we have previously investigated[3] the errors associated with our conformational ratio measurements from ^{19}F NMR spectroscopy and found that the largest error at the 95% confidence interval to be ±0.10 kJ mol^{-1} (twice the standard deviation), with an average 95% confidence interval of ±0.06 kJ mol^{-1} across the samples. This is within the ΔG_{exp} value range of −4 kJ mol^{-1} and +4 kJ mol^{-1}. Thus, an estimated minimum error of ±0.12 kJ mol^{-1} is likely to accommodate any systematic experimental variation.

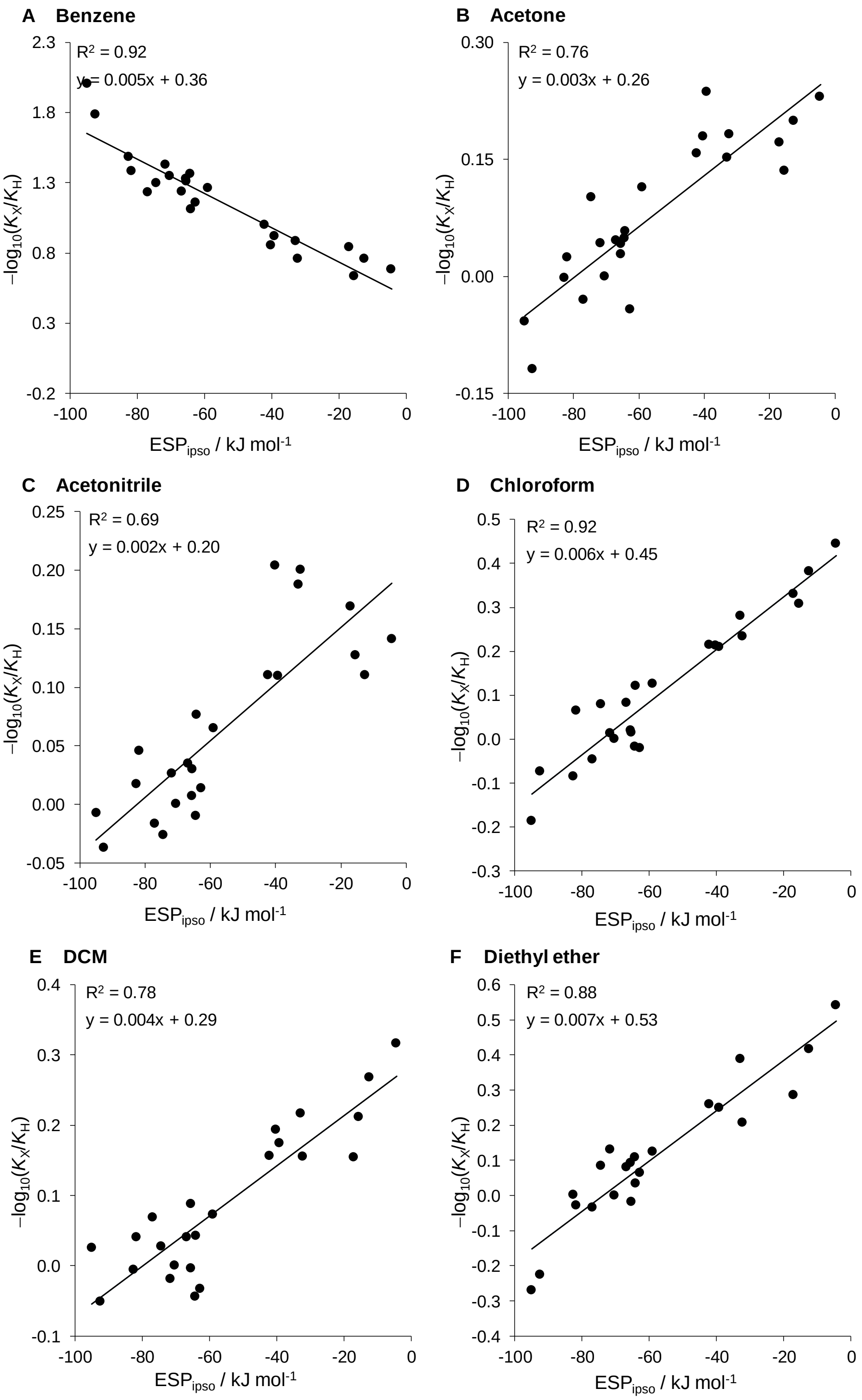

Figure S38. Plots of ESP_{ipso} values against $-\log_{10}(K_X/K_H)$ values for all balances obtained in **(A)** benzene-d_6, **(B)** acetone-d_6, **(C)** acetonitrile-d_3, **(D)** chloroform-d, **(E)** DCM-d_2 and **(F)** diethyl ether. All errors in $-\log_{10}(K_X/K_H)$ were 0.04. Structures and surfaces were minimized using DFT/B3LYP/6-31G*.

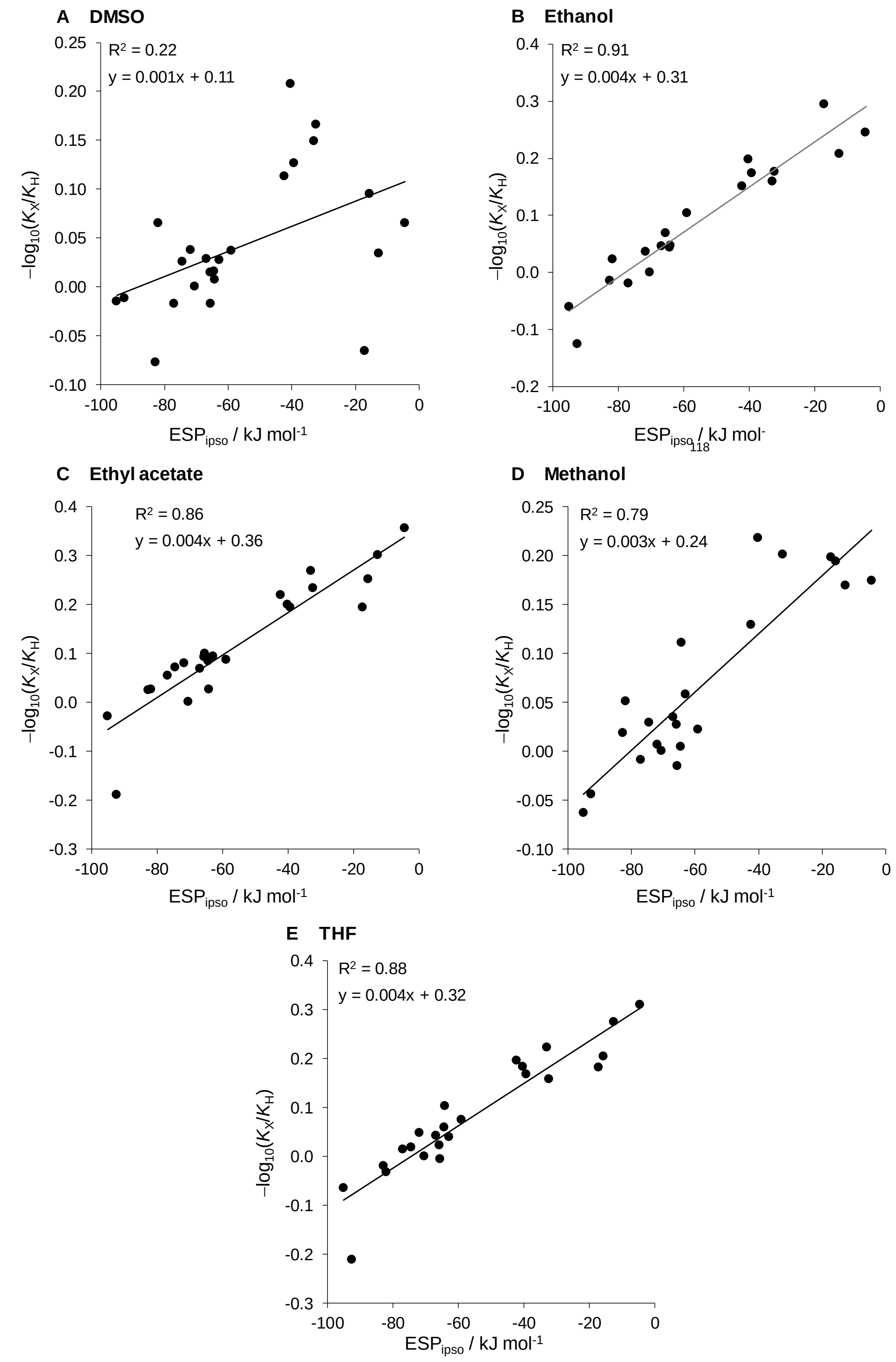

Figure S39. Plots of ESP_{ipso} values against $-\log_{10}(K_X/K_H)$ values for all balances obtained in **(A)** DMSO-d_3, **(B)** ethanol, **(C)** ethyl acetate, **(D)** methanol-d_2 and **(E)** THF-d_8. All errors in $-\log_{10}(K_X/K_H)$ were 0.04. Structures and surfaces were minimized using DFT/B3LYP/6-31G*.

Table S5. Values of ΔG_{exp} for series **1–X** in acetone-d_6, acetonitrile-d_3 and benzene-d_6 (376.5 MHz, 298 K). All values in kJ mol^{-1}. All errors were less than ±0.12 kJ mol^{-1}.

	acetone-d_6	acetonitrile-d_3	benzene-d_6
	ΔG_{exp}	ΔG_{exp}	ΔG_{exp}
**1–H**	-0.85	-0.78	-0.68
**1–OMe**	-0.71	-0.52	-0.75
**1–NEt$_2$**	-1.53	-0.99	-1.39
**1–Me**	-0.88	-0.85	-0.75
**1–Ph**	-0.58	-0.58	-0.46
**1–CN**	0.29	-0.15	0.78
**1–NO$_2$**	0.46	0.02	1.07
**1–CF$_3$**	0.15	0.10	0.55
**1–COCH$_3$**	0.50	-0.16	0.29
**1–Br**	0.05	-0.15	0.08
**1–a**	-1.18	-0.82	-1.68
**1–b**	-1.09	-0.70	-0.31
**1–c**	0.13	0.19	0.51
**1–d**	-0.68	-0.74	-0.61
**1–e**	-0.07	-0.05	1.23
**1–f**	-0.20	-0.41	-0.51
**1–g**	-0.86	-0.68	-0.93
**1–h**	0.19	0.36	0.70
**1–i**	-0.27	-0.93	-0.58
**1–j**	0.17	0.38	0.49
**1–k**	-0.61	-0.63	-0.83
**1–l**	-0.57	-0.84	-0.71
**1–m**	-0.61	-0.61	-0.64
**1–n**	-0.52	-0.34	-0.19

Table S6. Values of ΔG_{exp} for series **1–X** in chloroform-*d*, DCM-d_2 and diethyl ether (376.5 MHz, 298 K). All values in kJ mol^{-1}. All errors were less than ±0.12 kJ mol^{-1}. Those denoted as "n.s" were not soluble.

	chloroform-*d*	DCM-d_2	diethyl ether
	ΔG_{exp}	ΔG_{exp}	ΔG_{exp}
**1–H**	-1.14	-0.81	-1.11
**1–OMe**	-0.78	-0.58	-1.27
**1–NEt$_2$**	-1.57	-1.11	-2.40
**1–Me**	-1.14	-0.81	-1.27
**1–Ph**	-0.68	-0.58	-0.65
**1–CN**	1.03	0.71	1.27
**1–NO$_2$**	1.39	0.99	1.98
**1–CF$_3$**	0.40	0.29	0.78
**1–COCH$_3$**	0.05	0.18	0.32
**1–Br**	0.08	0.08	0.37
**1–a**	-2.21	-0.67	-2.65
**1–b**	-1.27	-1.00	-0.74
**1–c**	0.74	0.07	0.52
**1–d**	-1.04	-0.31	-0.58
**1–e**	0.60	0.39	*n.s*
**1–f**	-0.43	-0.40	-0.39
**1–g**	-1.63	-0.85	-1.09
**1–h**	0.19	0.07	0.07
**1–i**	-0.70	-0.66	-0.62
**1–j**	0.06	0.29	*N/A*[a]
**1–k**	-1.07	-0.92	-0.36
**1–l**	-1.25	-1.06	-0.49
**1–m**	-1.06	-0.84	-1.21
**1–n**	-0.46	-0.57	-0.92

Table S7. Values of ΔG_{exp} for series **1–X** in DMSO-d_6, ethanol and ethyl acetate (376.5 MHz, 298 K). All values in kJ mol^{-1}. All errors were less than ±0.12 kJ mol^{-1}. Those denoted as "n.s" were not soluble and "n.r" were not resolved.

	DMSO-d_6	ethanol	ethyl acetate
	ΔG_{exp}	ΔG_{exp}	ΔG_{exp}
**1–H**	-0.75	-0.81	-1.07
**1–OMe**	-0.37	-0.68	-0.92
**1–NEt_2**	-0.81	-1.53	-2.15
**1–Me**	-0.68	-0.88	-1.03
**1–Ph**	-0.58	-0.55	-0.68
**1–CN**	-0.55	0.37	0.64
**1–NO_2**	-0.37	0.58	0.96
**1–CF_3**	0.43	0.15	0.40
**1–$COCH_3$**	-0.02	0.18	0.03
**1–Br**	-0.10	0.05	0.18
**1–a**	-0.83	-1.16	-1.23
**1–b**	-0.59	*n.s*	-0.54
**1–c**	-1.12	0.87	0.04
**1–d**	-0.66	*n.s*	-0.54
**1–e**	-0.20	*n.s*	0.36
**1–f**	-0.54	-0.46	-0.57
**1–g**	-1.19	-1.07	-0.93
**1–h**	0.30	0.21	0.26
**1–i**	-0.60	*n.r*	-0.67
**1–j**	0.44	-0.32	0.07
**1–k**	-0.53	-0.65	-0.62
**1–l**	-0.66	-0.56	-0.59
**1–m**	-0.84	-0.42	-0.50
**1–n**	-0.70	-0.55	-0.92

Table S8. Values of ΔG_{exp} for series **1–X** in methanol-d_4 and THF-d_8 (376.5 MHz, 298 K). All values in kJ mol^{-1}. All errors were less than ±0.12 kJ mol^{-1}. Those denoted as "n.s" were not soluble and "n.r" were not resolved.

	methanol-d_4	THF-d_8
	ΔG_{exp}	ΔG_{exp}
**1–H**	-0.81	-0.88
**1–OMe**	-0.52	-1.07
**1–NEt$_2$**	-1.53	-2.09
**1–Me**	-0.85	-1.07
**1–Ph**	-0.62	-0.65
**1–CN**	0.15	0.68
**1–NO$_2$**	0.18	0.88
**1–CF$_3$**	*n.r*	0.49
**1–COCH$_3$**	*n.r*	0.07
**1–Br**	-0.08	0.23
**1–a**	-1.17	-1.26
**1–b**	-0.48	-0.66
**1–c**	0.32	0.15
**1–d**	-0.66	-0.76
**1–e**	0.29	0.28
**1–f**	-0.69	-0.46
**1–g**	-0.71	-1.00
**1–h**	0.33	0.01
**1–i**	-0.71	-0.78
**1–j**	0.43	0.16
**1–k**	-0.78	-0.61
**1–l**	-0.79	-0.55
**1–m**	-0.90	-0.92
**1–n**	-0.18	-0.30

S7. Linear Regression to Obtain Solvent-Independent Conformational Free Energies (ΔE)

Experimental conformational free energies ΔG_{solv} were fitted for each balance against ΔG_{solv} as defined by Equation S4 using the multiple linear regression tool in Origin 2019.

$$\Delta G_{solv} = \Delta E + \beta_s \Delta \alpha + \alpha_s \Delta \beta \qquad \textbf{Equation S4}$$

The β_s and α_s hydrogen bond constants for each solvent were locked as constants (listed Table S9), while the coefficients ΔE, $\Delta \alpha$ and $\Delta \beta$ were iteratively fitted variables to give the best agreement between the experimental ΔG_{solv} values and modelled ΔG_{solv} values (across eleven solvents for each balance). The fitting of ΔG_{solv} against ΔG_{exp} gave a correlation with $R^2 = 0.85$ (Figure S40).

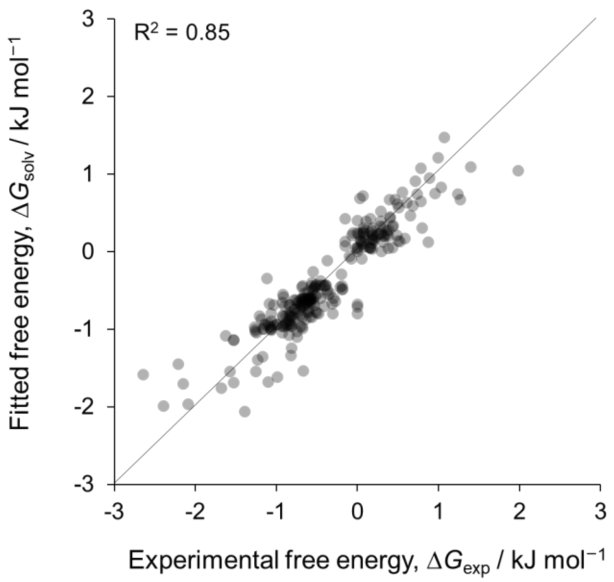

Figure S40. Plot of fitted ΔG_{solv} values for all molecular balances examined in eleven solvents vs. the experimentally determined conformational free energies, ΔG_{exp}.

The output coefficients ΔE, $\Delta \alpha$ and $\Delta \beta$ and corresponding fitting errors $\delta \Delta E$, $\delta \Delta \alpha$ and $\delta \Delta \beta$ for each balance are listed in Table S10. The fitted ΔG_{Solv} values are reported in Tables S11-S13, with the errors in ΔG_{Solv} ($\delta \Delta G$) being defined by Equation S5.

$$\delta\Delta G_{Solv} = \sqrt{(\delta\Delta E)^2 + (\delta\Delta\alpha)^2 + (\delta\Delta\beta)^2} \qquad \textbf{Equation S5}$$

ΔE values were correlated against ESP_{ipso} and this plot, with error bars included, is given in Figure S41.

Table S9. α_s and β_s values used in multiple linear regression to obtain ΔE.[6]

Solvent	α_s	β_s
Chloroform	2.2	0.9
Acetone	1.5	5.8
Acetonitrile	1.7	5.1
Benzene	1.1	2.1
Ethyl acetate	1.5	5.3
THF	0.9	5.9
DCM	1.9	1.1
Ethanol	2.7	5.3
Methanol	2.7	5.3
DMSO	2.2	8.7
Diethyl ether	0.9	5.3

Table S10. ΔE, $\Delta\alpha$ and $\Delta\beta$ values of series **1–X** obtained from multiple linear regression analysis to obtain ΔG_{solv} with their associated errors as output by Origin 2019.

Compound	ΔE / kJ mol^{-1}	$\delta\Delta E$	$\Delta\beta$	$\delta\Delta\beta$	$\Delta\alpha$	$\delta\Delta\alpha$
**1–H**	-1.09	0.18	0.05	0.02	0.02	0.08
**1–OMe**	-1.49	0.22	0.33	0.03	0.03	0.10
**1–NEt_2**	-2.61	0.52	0.47	0.07	0.04	0.23
**1–Me**	-1.15	0.20	0.10	0.03	0.01	0.09
**1–Ph**	-0.59	0.08	0.00	0.01	0.00	0.03
**1–CN**	1.74	0.40	-0.35	0.05	-0.14	0.18
**1–NO_2**	2.31	0.53	-0.49	0.07	-0.16	0.24
**1–CF_3**	1.21	0.22	-0.33	0.03	-0.05	0.10
**1–$COCH_3$**	0.35	0.23	-0.10	0.03	0.10	0.11
**1–Br**	0.44	0.14	-0.16	0.02	-0.02	0.06
**1–a**	-2.23	0.65	0.33	0.08	0.07	0.29
**1–b**	-0.82	0.35	-0.09	0.04	0.05	0.17
**1–c**	0.88	0.51	0.00	0.07	-0.14	0.23
**1–d**	-0.58	0.24	-0.05	0.03	0.00	0.11
**1–e**	1.23	0.39	-0.22	0.04	0.12	0.19
**1–f**	-0.40	0.17	-0.01	0.02	0.00	0.08
**1–g**	-1.09	0.30	-0.01	0.04	0.03	0.14
**1–h**	0.37	0.25	-0.02	0.03	-0.02	0.11
**1–i**	-0.64	0.22	-0.03	0.03	0.01	0.10
**1–j**	0.15	0.20	0.04	0.02	0.01	0.09
**1–k**	-0.83	0.11	-0.10	0.01	0.07	0.05
**1–l**	-0.72	0.15	-0.17	0.02	0.06	0.07
**1–m**	-0.97	0.28	0.07	0.04	0.01	0.13
**1–n**	-0.47	0.29	0.06	0.04	-0.03	0.13

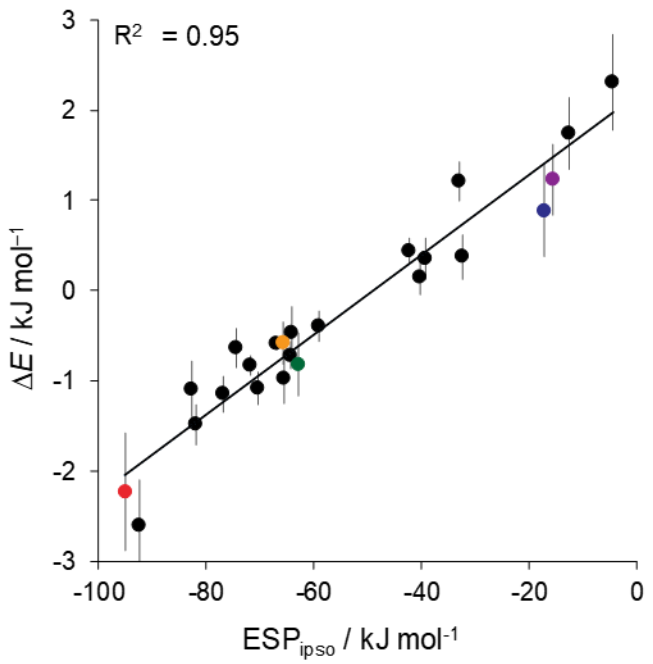

Figure S41. Plot of dissected solvent-independent Δ*E* values against ESP_{ipso} as given in main text Figure 5, but with error bars included.[1-3]

Table S11. Values of ΔG_{solv} for series **1–X** obtained from multiple linear regression analysis with their associated errors in acetone-d_6, acetonitrile-d_3 and benzene-d_6. All values in kJ mol^{-1}.

		acetone-d_6	acetonitrile-d_3	benzene-d_6
	$\delta\Delta G_{solv}$	ΔG_{solv}	ΔG_{solv}	ΔG_{solv}
**1–H**	0.23	-0.88	-0.89	-0.68
**1–OMe**	0.25	-0.82	-0.77	-0.75
**1–NEt$_2$**	0.57	-1.68	-1.61	-1.39
**1–Me**	0.22	-0.94	-0.93	-0.75
**1–Ph**	0.08	-0.60	-0.60	-0.46
**1–CN**	0.44	0.40	0.42	0.78
**1–NO$_2$**	0.58	0.68	0.69	1.07
**1–CF$_3$**	0.24	0.43	0.40	0.55
**1–COCH$_3$**	0.26	0.14	0.13	0.23
**1–Br**	0.15	0.10	0.08	0.08
**1–a**	0.72	-1.36	-1.38	-1.82
**1–b**	0.39	-0.63	-0.71	-0.32
**1–c**	0.56	0.10	0.18	0.68
**1–d**	0.26	-0.61	-0.62	-0.71
**1–e**	0.44	0.17	0.20	1.48
**1–f**	0.19	-0.44	-0.44	-0.42
**1–g**	0.34	-0.94	-0.96	-1.04
**1–h**	0.27	0.22	0.23	0.31
**1–i**	0.24	-0.64	-0.65	-0.65
**1–j**	0.22	0.28	0.28	0.22
**1–k**	0.12	-0.58	-0.65	-0.78
**1–l**	0.17	-0.63	-0.68	-0.58
**1–m**	0.31	-0.69	-0.71	-0.62
**1–n**	0.32	-0.55	-0.52	-0.13

Table S12. Values of ΔG_{solv} for series **1–X** obtained from multiple linear regression analysis with their associated errors in chloroform-*d*, DCM-d_2, diethyl ether and DMSO-d_6. All values in kJ mol^{-1}.

	chloroform-*d*	DCM-d_2	diethyl ether	DMSO-d_6
	ΔG_{solv}	ΔG_{solv}	ΔG_{solv}	ΔG_{solv}
**1–H**	-1.14	-0.96	-0.92	-0.78
**1–OMe**	-0.78	-0.83	-1.03	-0.50
**1–NEt$_2$**	-1.57	-1.67	-1.98	-1.24
**1–Me**	-1.14	-0.95	-1.00	-0.84
**1–Ph**	-0.68	-0.59	-0.60	-0.61
**1–CN**	1.03	0.91	0.68	-0.25
**1–NO$_2$**	1.39	1.21	1.05	-0.11
**1–CF$_3$**	0.40	0.53	0.65	0.05
**1–COCH$_3$**	0.13	0.16	0.21	0.05
**1–Br**	0.08	0.11	0.21	-0.06
**1–a**	-2.27	-1.75	-1.58	-0.87
**1–b**	-1.18	-0.96	-0.54	-0.62
**1–c**	0.49	0.80	0.26	-0.46
**1–d**	-0.96	-0.69	-0.60	-0.58
**1–e**	0.62	0.69	0.47	-0.50
**1–f**	-0.43	-0.43	-0.43	-0.46
**1–g**	-1.08	-1.07	-0.95	-0.87
**1–h**	0.31	0.31	0.25	0.15
**1–i**	-0.69	-0.68	-0.63	-0.64
**1–j**	0.26	0.25	0.25	0.34
**1–k**	-1.00	-0.95	-0.55	-0.45
**1–l**	-1.03	-0.90	-0.63	-0.52
**1–m**	-0.99	-0.82	-0.72	-0.59
**1–n**	-0.37	-0.34	-0.52	-0.69

Table S13. Values of ΔG_{solv} for series **1–X** obtained from multiple linear regression analysis with their associated errors in acetone-d_6, acetonitrile-d_3 and benzene-d_6. All values in kJ mol^{-1}.

	ethanol	ethyl acetate	methanol-d_4	THF-d_8
	ΔG_{solv}	ΔG_{solv}	ΔG_{solv}	ΔG_{solv}
**1–H**	-0.83	-0.89	-0.83	-0.91
**1–OMe**	-0.44	-0.83	-0.44	-1.01
**1–NEt$_2$**	-1.14	-1.70	-1.14	-1.96
**1–Me**	-0.83	-0.94	-0.83	-1.00
**1–Ph**	-0.60	-0.60	-0.60	-0.60
**1–CN**	0.05	0.47	0.05	0.59
**1–NO$_2$**	0.17	0.76	0.17	0.96
**1–CF$_3$**	0.05	0.45	0.05	0.62
**1–COCH$_3$**	0.04	0.15	0.04	0.20
**1–Br**	-0.09	0.11	-0.09	0.20
**1–a**	-1.09	-1.41	-1.09	-1.51
**1–b**	-0.90	-0.66	-0.90	-0.51
**1–c**	0.02	0.18	0.02	0.16
**1–d**	-0.65	-0.62	-0.65	-0.59
**1–e**	-0.21	0.24	-0.21	0.38
**1–f**	-0.45	-0.44	-0.45	-0.43
**1–g**	-0.97	-0.96	-0.97	-0.94
**1–h**	0.21	0.23	0.21	0.23
**1–i**	-0.68	-0.64	-0.68	-0.62
**1–j**	0.33	0.27	0.33	0.26
**1–k**	-0.74	-0.61	-0.74	-0.51
**1–l**	-0.72	-0.66	-0.72	-0.60
**1–m**	-0.68	-0.71	-0.68	-0.71
**1–n**	-0.54	-0.53	-0.54	-0.55

S8. Synthetic Procedures and Standard Characterization Data

All chemicals were obtained from commercial sources and used as received. All reactions were performed under a nitrogen atmosphere and using degassed solvents unless stated otherwise. Dry solvent use is stated and such solvents were HPLC grade and collected from the departmental Solvent Purification Facility (SPS). Analytical TLC was carried out on Merck aluminium sheets coated with silica gel 60F and visualized using UV light (254 nm). Preparatory TLC was carried out on Analtech 20 x 20 cm glass mounted plates on 2000 micron silica and flash chromatography was performed using silica gel Geduran 60 (40 – 63 μm). Solvent ratios have been indicated in brackets. Mass spectrometry was performed by the University of Edinburgh technician-supported mass spectrometry service, using a ThermoElectron MAT XP spectrometer for EI-HRMS and ESI-HRMS.

IR spectra were obtained on neat samples using a Shimadzu IR Affinity-1 machine. Absorptions are reported in frequency of absorption (cm^{-1}). Absorptions in the fingerprint region are not reported. ^{1}H and ^{13}C NMR spectra were recorded on a 500 MHz Bruker Avance III spectrometer. ^{19}F NMR spectra were recorded on a 400 or 500 MHz Bruker Avance III spectrometer. NMR chemical shifts (δ) are reported in parts per million (ppm) relative to trimethylsilane (δ = 0 ppm) or $CDCl_3$ (^{1}H δ = 7.26 ppm, ^{13}C δ = 77.16 ppm) as an internal reference.[7] All ^{13}C were ^{1}H decoupled and ^{19}F spectra with poor resolution were ^{1}H decoupled. Coupling constants, J, are reported in Hertz (Hz).

S8.1 General procedure for copper-mediated coupling of halophenyls to aryl-amides

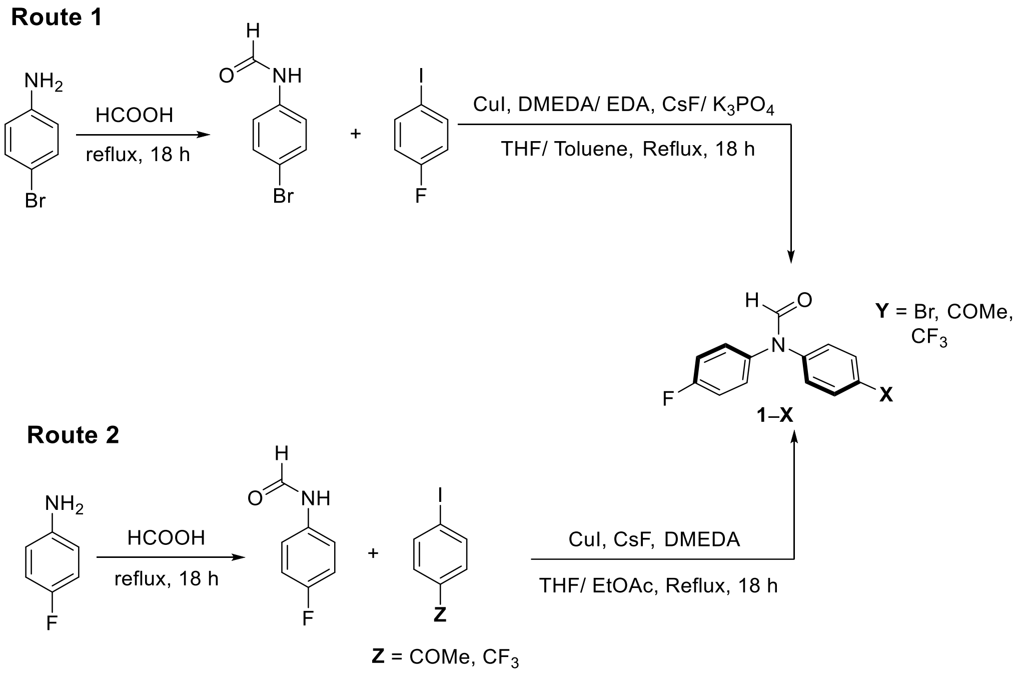

A subset of series **1–X** were prepared according to the same general copper (I) iodide catalysed cross-coupling conditions.[8] An oven dried flask was sealed before evacuating and back filling with nitrogen three times before the amide, aryl halide, catalyst and base (CsF/ K_3PO_4) were added. The flask was evacuated and back-filled with nitrogen a further three times. Dry, degassed solvent (THF / toluene) and ligand (*N,N*-dimethylethylenediamine (DMEDA) / ethylenediamine (EDA)) were added *via* syringe and the suspension was heated at reflux for 18 h under a nitrogen atmosphere. The reaction mixture was then cooled to ambient temperature, diluted with EtOAc or DCM and quenched with saturated ammonium chloride. The aqueous phase was extracted with organic solvent and the combined organic phases were washed with brine, dried over $MgSO_4$ and concentrated *in vacuo*. The resulting products were further purified by chromatography. **Route 1** was followed for **1–Br**, while **Route 2** was used to obtain **1–COMe** and **1–CF$_3$**. Amine formylation is detailed in ***Section S8.4***.

N-(4-fluorophenyl)-N-(4-(trifluoromethyl)phenyl)formamide (1–CF_3)

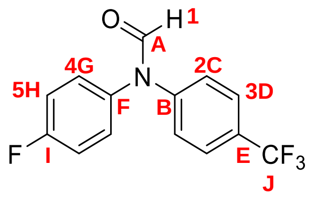

Prepared using the general copper-mediated cross coupling procedure: THF (2 mL), 4-iodobenzotrifluoride (396 mg, 0.214 ml, 1.46 mmol), *N*-(4-fluorophenyl)formamide (205 mg, 1.48 mmol), CsF (441 mg, 2.9 mmol), Cu(I)I (140 mg, 0.74 mmol) and DMEDA (18 µL, 0.17 mmol). Purification by preparative TLC (1:1 EtOAc: *n*-Hex) yielded **1–CF_3** as a light yellow solid (217 mg, 1.47 mmol, 52.5%).

^{1}H NMR (500 MHz, $CDCl_3$) δ 8.78 (1', s, 1H), 8.62 (1, s, 1H), 7.70 (3', d, J = 8.4 Hz, 2H), 7.66 (3, d, J = 8.5 Hz, 2H), 7.46 (2, d, J = 8.4 Hz, 2H), 7.29 – 7.24 (2, 4', m, 4H), 7.25 – 7.20 (4, m, 2H), 7.20 – 7.14 (5, 5', m, 4H); ^{13}C NMR (126 MHz, $CDCl_3$) δ 161.96 (I, d, J = 249.0 Hz), 161.54 (I', d, J = 248.3 Hz), 161.53 (A), 161.26 (A'), 144.78 (B'), 142.84 (B), 136.83 (F), 134.64 (F'), 129.40 – 127.90 (E, E', m, partially lost to noise), 128.55 (G, d, J = 8.6 Hz), 128.14 (G', d, J = 8.7 Hz), 127.04 (D', dd, J = 7.3, 3.7 Hz), 126.30 (D, dd, J = 7.3, 3.7 Hz), 125.05 (C), 123.86 (C), 126.98 – 120.30 (J, J', m, partially lost to noise), 117.11 (H, d, J = 23.0 Hz), 116.58 (H', d, J = 22.9 Hz); MP: 53 – 56 °C; ^{19}F NMR (376.5 MHz, $CDCl_3$) δ −62.51 (major), −112.80 (minor); EI-HRMS: obtained *m/z* 283.061428 M^+ (expected *m/z* 283.06148 M^+).

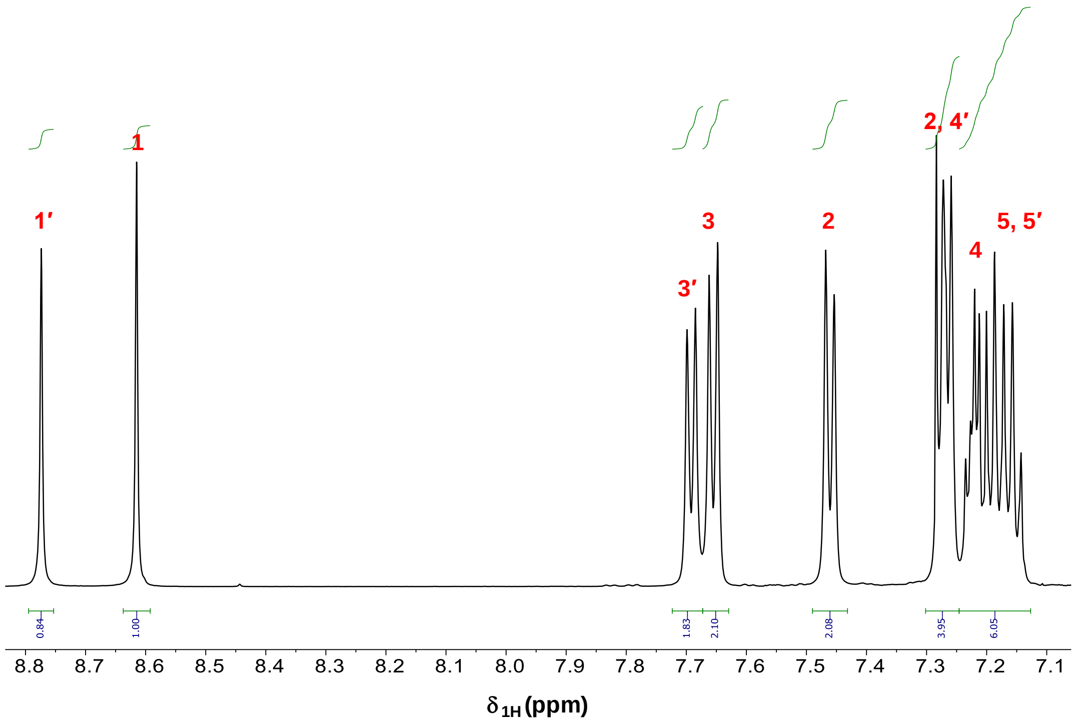

N-(4-pyridyl)-*N*-(4-acetophenyl)formamide (1–COMe)

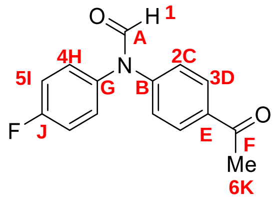

Prepared using the general copper-mediated cross coupling procedure: THF (2 mL), 4-iodoacetophenone (180 mg, 0.73 mmol), *N*-(4-fluoro-phenyl)formamide (90 mg, 0.65 mmol), CsF (187 mg, 1.23 mmol), Cu(I)I (59 mg, 0.31 mmol) and DMEDA (9 µL, 0.08 mmol). Purification by preparative TLC (2:1 EtOAc: *n*-Hex) yielded **1–COMe** as a light-yellow solid (103 mg, 0.65 mmol, 61.9%).

^{1}H NMR (500 MHz, $CDCl_3$) δ 8.82 (1’, s, 1H), 8.62 (1, s, 1H), 8.02 (3’, d, J = 8.5 Hz, 1H), 7.99 (3, d, J = 8.6 Hz, 1H), 7.44 (2, d, J = 8.6 Hz, 2H), 7.28 – 7.25 (4, m, 2H), 7.22 (4’, 2’, m, 4H), 7.20 – 7.13 (5, 5’, m, 4H), 2.63 (6, s, 1H), 2.62 (6’, s, 1H); ^{13}C NMR (126 MHz, $CDCl_3$) δ 196.91 (F), 196.60 (F’), 161.98 (J’, d, J = 249.3 Hz), 161.59 (J, d, J = 248.2 Hz), 161.56 (A), 160.29 (A’), 145.73 (B’), 143.92 (B), 136.84 (G), 135.15 (E’), 134.79 (E), 134.57 (G’), 130.07 (D’), 129.37 (D), 128.69 (H, d, J = 8.6 Hz), 128.27 (H’ d, J = 8.3 Hz), 124.55 (C), 123.20 (C’), 117.09 (I, d, J

= 22.9 Hz), 116.58 (I', d, J = 23.0 Hz), 26.60 (K, K'); ^{19}F NMR (376.5 MHz, $CDCl_3$) δ −112.78 (major), −113.30 (minor); EI-HRMS: obtained *m/z* 257.084534 M^+ (expected *m/z* 257.08466 M^+)

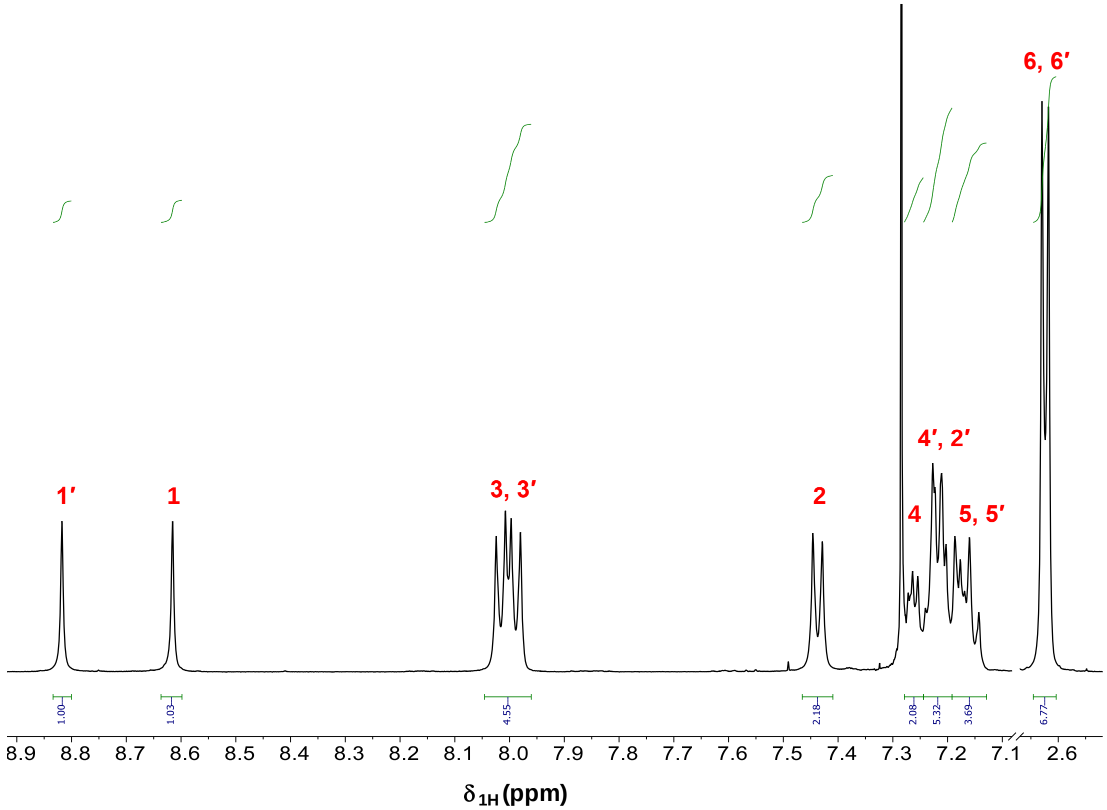

N-(4-Bromophenyl)-N-(4-fluorophenyl)formamide (1–Br)

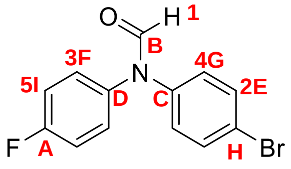

Prepared using the general copper-mediated cross coupling procedure: THF (2 mL), *N*-(4-bromophenyl)formamide (108 mg, 0.54 mmol), 1-fluoro-4-iodobenzene (0.05 mL, 0.45 mmol), Cu(I)I (43 mg, 0.23 mmol), CsF (171 mg, 1.13 mmol) and DMEDA (4.8 µL, 0.04 mmol). Purification with flash chromatography (1:1 *n*-hex: DCM then 100% EtOAc) yielded **1–Br** as a brown solid (113 mg, 0.38 mmol, 85%). υ_{max} (neat) /cm^{-1} 1668.43 (C=O), 1639.49, 1602.85, 1583.56, 1504.48, 1485.19; ^{1}H NMR (500 MHz, $CDCl_3$) δ 8.63 (1', s, 1H), 8.57 (1, s, 1H), 7.54 (2', m, 2H), 7.51 (2, m, 2H), 7.25 (3', m, 2H), 7.18 (4, m, 2H), 7.16 (3, m, 2H), 7.12 (5, m, 2H), 7.10 (5', m, 2H), 7.03 (4', m, 2H); ^{13}C NMR (126 MHz, $CDCl_3$) δ 161.82 (A, d, J = 248.4 Hz), 161.34 (A', d, J = 247.7 Hz), 161.52 (B'), 161.40 (B), 140.85 (C'), 138.86 (C), 137.33 (D, d, J = 3.1 Hz), 135.20 (D', d, J = 3.2 Hz), 133.10 (E'), 132.44 (E), 128.11 (F', d, J = 8.5 Hz), 127.62 (F, d, J = 8.6 Hz), 127.14

(G), 126.30 (G'), 120.86 (H'), 120.27 (H), 117.03 (I, d, J = 22.9 Hz), 116.44 (I', d, J = 22.8 Hz); ^{19}F NMR (376.5 MHz, $CDCl_3$) δ −113.50 (major), −113.99 (minor); EI-HRMS: obtained m/z 292.984671 M^+ (expected m/z 292.98515 M^+).

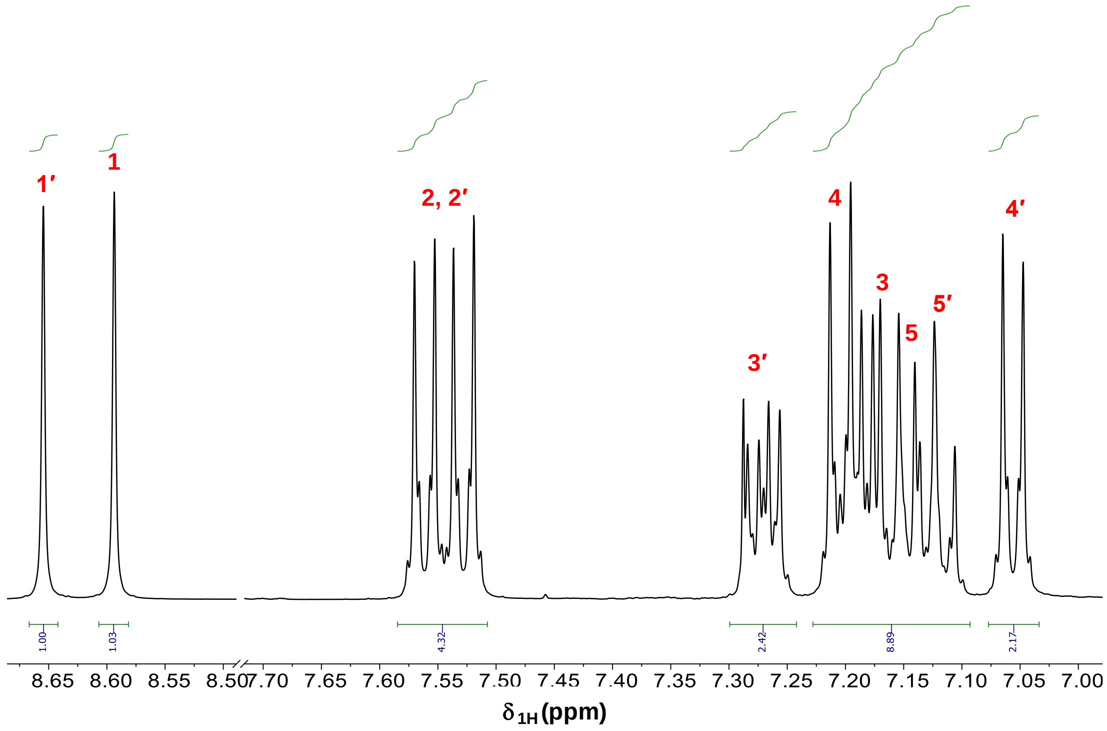

S8.2 General procedure for palladium-mediated cross-coupling of phenylboronic acid derivatives to *N*-(4-Bromophenyl)-*N*-(4-fluorophenyl)formamide (1–Br)

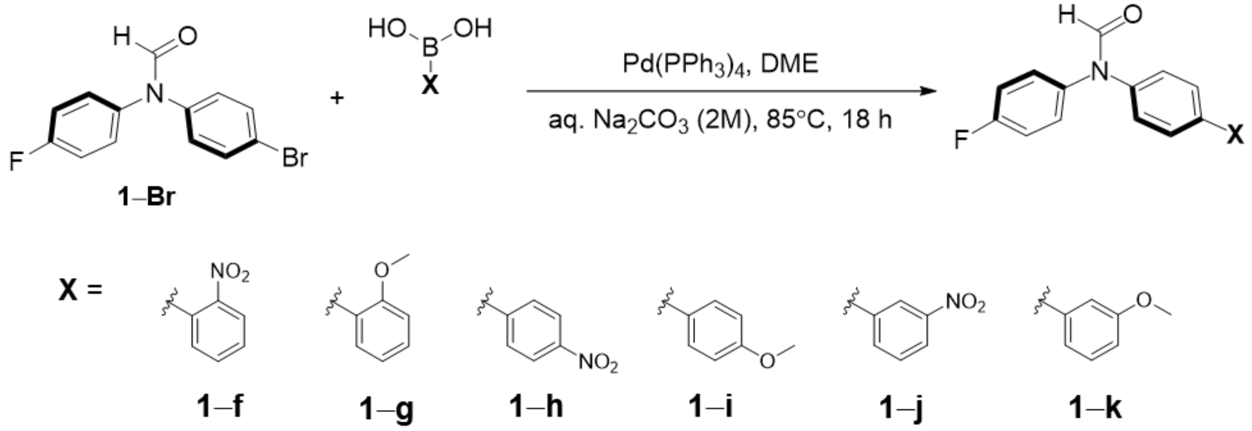

A subset of series **1–X** were prepared according to the same general palladium-mediated cross-coupling conditions.[9] An oven dried flask was sealed before evacuating and back filling with nitrogen three times before *N*-(4-bromophenyl)-*N*-(4-fluorophenyl)formamide, the phenylboronic acid derivative and $Pd(PPh_3)_4$ were added. The flask was evacuated and back filled with nitrogen a further three times. Degassed dimethoxyethane (DME) and aq. Na_2CO_3 (2M) were added *via* syringe and the reaction mixture was heated at 85 °C for 18 h under a nitrogen atmosphere. The reaction mixture was then cooled to ambient temperature, quenched with sat. aq. NH_4Cl and extracted with $CHCl_3$. The combined organic extracts were dried over $MgSO_4$ before concentration *in vacuo*. The resulting products were further purified by chromatography.

N-(3-nitro-[1,1-biphenyl]-4-yl)-N-(4-fluorophenyl)-formamide (1−f)

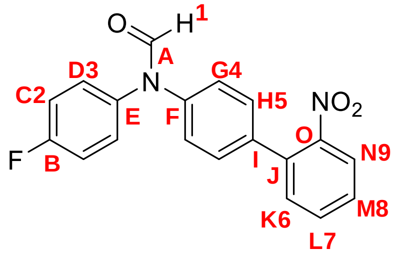

Prepared using the general palladium-mediated cross coupling procedure: *N*-(4-bromophenyl)-*N*-(4-fluorophenyl)formamide (251 mg, 0.85 mmol), $Pd(Ph_3P)_4$ (29.4 mg, 0.03 mmol), (2-nitrophenyl)boronic acid (198.5 mg, 1.19 mmol) DME (11 ml) and aq. Na_2CO_3 (2 M, 2 mL). Purified with preparative TLC (4:1 *n*-Hex: EtOAc) to yield **1−f** an orange solid (150.3 mg, 0.45 mmol, 52 %).

υ_{max} (neat) /cm^{-1} 2922.37, 1682.96 (C=O), 1516.23, 1265.07, 1218.01, 835.78, 737.37; ^{1}H NMR (500 MHz, $CDCl_3$) δ ; 8.77 (1, s, 1H), 8.63 (1’, s, 1H), 7.93 (6, m, 1H), 7.89 (6’, m, 1H), 7.66 (8 and 8’, m, 2H), 7.54 (7 and 7’, m, 2H), 7.46 (9 and 9’, m, 2H), 7.38 (5, 5’ and 4’, m, 3H), 7.36 – 7.31 (3’, m, 1H), 7.27 – 7.22 (3’ and 4’, m, 2H), 7.20 – 7.13 (2 and 2’, m, 2H); ^{13}C NMR (126 MHz, $CDCl_3$) δ 161.75 (B, d, J = 248.3 Hz), 161.60 (A), 161.58 (A’), 161.30 (B’, d, J = 247.5 Hz), 149.18 (O’), 149.12 (O), 141.63 (I), 139.71 (F’), 137.33 (E’, d, J = 3.1 Hz), 136.24 (J), 135.67 (I’), 135.49 (J’), 135.15 (E and F’ overlapped, d, J = 4.0 Hz), 132.54 (M), 132.43 (M’), 131.96 (N’), 131.82 (N), 129.43 (L), 128.81 (L’), 128.69 (H’), 128.43 (G’), 127.78 (D, d, J = 8.6 Hz), 124.37 (D’, d, J = 5.0 Hz), 124.39 (G), 124.35 (K), 124.19 (K’), 116.89 (C’, d, J = 22.9 Hz), 116.34 (C, d, J = 22.8 Hz); ^{19}F NMR (376.5 MHz, $CDCl_3$) δ −113.61 (minor), −114.09 (major); EI-HRMS: obtained *m/z* 336.08932 M^+ (expected *m/z* 336.09047 M^+).

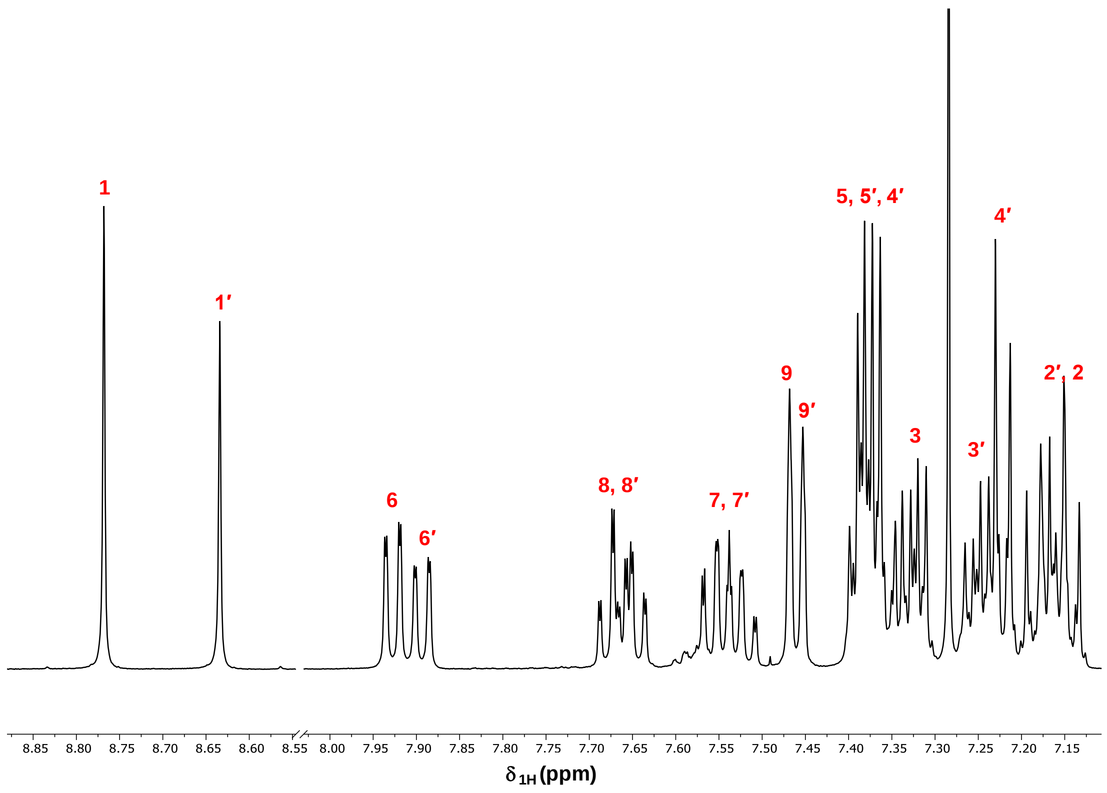
1
1′
6
6′
8, 8′
7, 7′
9
9′
5, 5′, 4′
3
3′
4′
2′, 2
8.85 8.80 8.75 8.70 8.65 8.60 8.55 8.00 7.95 7.90 7.85 7.80 7.75 7.70 7.65 7.60 7.55 7.50 7.45 7.40 7.35 7.30 7.25 7.20 7.15
δ1H (ppm)

N-(2-methoxy-[1,1-biphenyl]-4-yl)-N-(4-fluorophenyl)-formamide (1–g)

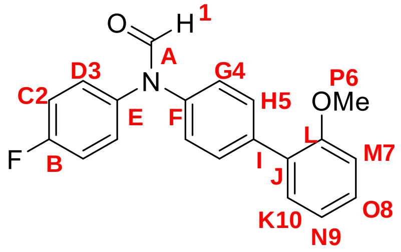

Prepared using the general palladium-mediated cross coupling procedure: N-(4-bromophenyl)-N-(4-fluorophenyl)formamide (251 mg, 0.85 mmol), $Pd(Ph_3P)_4$ (29.47 mg, 0.03 mmol), (2-methoxyphenyl)boronic acid (180.9 mg, 1.19 mmol) DME (11 ml) and aq. Na_2CO_3 (2 M, 2 mL). Purified with flash chromatography (4:1 *n*-Hex: EtOAc) to yield **1–g** a white solid (195.2 mg, 0.61 mmol, 72%).

υ_{max} (neat) /cm^{-1} 2968.01, 2936.63, 2923.79, 1686.54 (C=O), 1595.96, 1506.11, 1488.29, 1272.21, 835.78, 770.17; ^{1}H NMR (500 MHz, $CDCl_3$) δ 8.74 (1, s, 1H), 8.64 (1', s, 1H), 7.61 – 7.57 (5, 9 and 5', m, 5H), 7.39 – 7.33 (8, 8', 10, 3 and 4', m, 6H), 7.27 – 7.24 (10' and 3', m, 3H), 7.21 – 7.19 (4 and 9', m, 3H), 7.17 – 7.12 (2' and 2, m, 4H), 7.09 – 7.00 (7 and 7', m, 2H), 3.86 (6, s, 3H), 3.84 (6', s, 3H); ^{13}C NMR (126 MHz, $CDCl_3$) δ 161.82 (A), 161.60 (A'), 161.58 (B', d, J = 247.4 Hz), 161.12 (B, d, J = 246.9 Hz), 156.47 (L'), 156.37 (L), 140.23 (I), 138.29 (F'), 137.76 (E', d, J = 3.2 Hz), 137.54 (F), 137.10 (J), 136.81 (J'), 135.52 (E, d, J = 3.1 Hz), 130.87 (K'), 130.81 (I), 130.69 (H), 130.33 (H'), 129.65 (I'), 129.16 (N), 128.89 (N'), 128.09 (D, d, J = 8.5 Hz), 127.52 (D', d, J = 8.5 Hz), 125.02 (G'), 124.23 (G), 121.00 (M), 120.92 (M'), 116.68 (C', d, J = 22.8 Hz), 116.14 (C, d, J = 22.7 Hz), 111.28 (O and O' overlapped), 55.55 (P), 55.51 (P'); ^{19}F NMR (376.5 MHz, $CDCl_3$) δ −114.21 (minor), −114.61 (major); EI-HRMS: obtained *m/z* 321.11619 M^+ (expected *m/z* 321.11596 M^+).

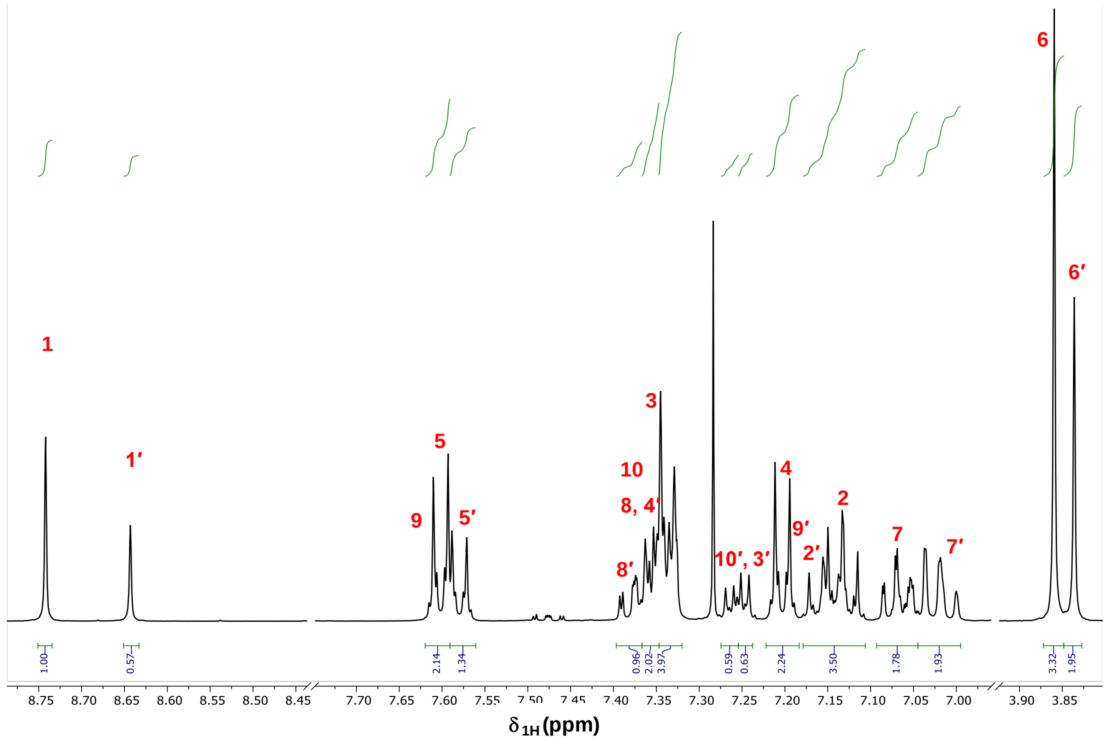

1
1′
9
5
5′
10
8, 4′
3
8′
10′, 3′
4
9′
2′
2
7
7′
6
6′
δ1H (ppm)

N-(4-nitro-[1,1-biphenyl]-4-yl)-N-(4-fluorophenyl)-formamide (1−h)

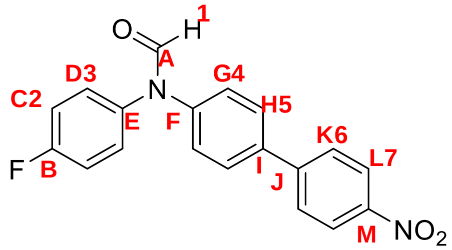

Prepared using the general palladium-mediated cross coupling procedure: *N*-(4-bromophenyl)-*N*-(4-fluorophenyl)formamide (249.0 mg, 0.85 mmol), $Pd(Ph_3P)_4$ (29.3 mg, 0.03 mmol), (4-nitrophenyl)boronic acid (198.5 mg, 1.19 mmol) DME (11 mL) and aq. Na_2CO_3 (2 M, 2 mL). Purified using preparative TLC (3:1 *n*-Hex: EtOAc) to yield **1−h** a dark orange solid (183.0 mg, 0.54 mmol, 64%).

υ_{max} (neat) / cm^{-1} ; 2920.94, 1677.26 (C=O), 1593.24, 1340.67, 1219.44, 825.79; ^{1}H NMR (500 MHz, $CDCl_3$) δ 8.78 (1’, s, 1H), 8.65 (1, s, 1H), 8.37 – 8.29 (7 and 7’, m, 4H), 7.77 – 7.72 (6 and 3, m, 4H), 7.71 – 7.64 (4 and 3’, m, 4H), 7.49 – 7.43 (5’, m, 2H), 7.35 – 7.29 (4’ and 5, m, 4H), 7.27 – 7.24 (6’, m, 2H), 7.21 – 7.11 (2 and 2’. m, 4H); ^{13}C NMR (126 MHz, $CDCl_3$) δ 161.79 (B’, d, J = 248.7 Hz), 161.61 (A’), 161.44 (A), 161.34 (B, d, J = 247.8 Hz), 147.38 (M’), 147.24 (M), 146.52 (F), 146.06 (F’), 142.31 (I’), 140.41 (I), 137.44 (J), 137.26 (E’, d, J = 3.1 Hz), 137.02 (J’), 135.08 (E, d, J = 3.2 Hz), 128.79 (G), 128.26 (D, d, J = 8.6 Hz), 128.18 (G’), 127.74 (H and H’ overlapped, 127.69 (D’, d, J = 5.0 Hz), 125.93 (K), 124.88 (K’), 124.30 (L’), 124.23 (L), 116.95 (C’, d, J = 23.0 Hz), 116.39 (C, d, J = 22.7 Hz); ^{19}F NMR (376.5 MHz, $CDCl_3$) δ −113.38 (major), −113.84 (minor); EI-HRMS: obtained *m/z* 336.08917 M^+ (expected *m/z* 336.09047 M^+)

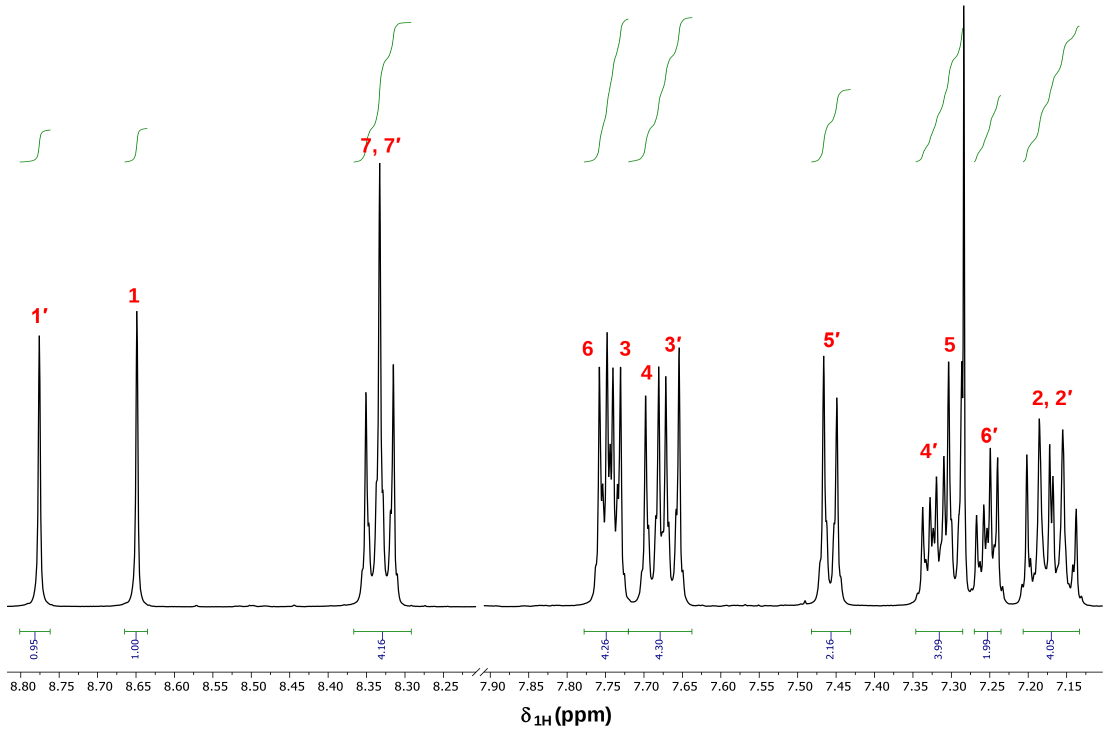

1′
1
7, 7′
6
3
4
3′
5′
5
4′
6′
2, 2′
0.95
1.00
4.16
4.26
4.30
2.16
3.99
1.99
4.05
8.80 8.75 8.70 8.65 8.60 8.55 8.50 8.45 8.40 8.35 8.30 8.25 7.90 7.85 7.80 7.75 7.70 7.65 7.60 7.55 7.50 7.45 7.40 7.35 7.30 7.25 7.20 7.15
δ 1H (ppm)

N-(4-methoxy-[1,1-biphenyl]-4-yl)-N-(4-fluorophenyl)-formamide (1–i)

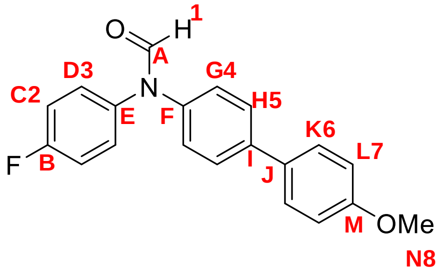

Prepared using the general palladium-mediated cross coupling procedure: *N*-(4-bromophenyl)-*N*-(4-fluorophenyl)formamide (250.8 mg, 0.85 mmol), $Pd(Ph_3P)_4$ (29.1 mg, 0.03 mmol), (4-methoxyphenyl)boronic acid (180.5 mg, 1.19 mmol) DME (11 mL) and aq. Na_2CO_3 (2 M, 2 mL). Purified using flash column chromatography (4:1 *n*-Hex: EtOAc) to yield **1–i** a white solid (155.0 mg, 0.48 mmol, 57%).

υ_{max} (neat) / cm^{-1} ; 3002.24, 2965.15, 2885.28, 1697.23 (C=O), 1505.74, 1330.68, 1210.88, 815.81; ^{1}H NMR (500 MHz, $CDCl_3$) δ 8.72 (1, s, 1H), 8.65 (1’, s, 1H), 7.61 – 7.57 (5 and 5’, m, 4H), 7.54 – 7.52 (7 and 7’, m, 4H), 7.36 – 7.32 (4’ and 3, m, 4H), 7.25 – 7.21 (3’ and 4, m, 4H), 7.17 – 7.11 (2’ and 2, m, 4H), 7.02 – 6.99 (6 and 6’, m, 4H), 3.88 (8, s, 3H), 3.88 (8’, s, 3H); ^{13}C NMR (126 MHz, $CDCl_3$) δ 162.53 (B’, d, J = 247.7 Hz), 161.67 (A), 161.59 (A’), 161.22 (B, d, J = 246.9 Hz), 159.54 (M), 159.37 (M’), 140.17 (I’), 139.89 (I), 139.49 (F’), 138.23 (F), 137.76 (E’, d, J = 3.1 Hz), 135.57 (E, d, J = 3.1 Hz), 132.75 (J’), 132.25 (J), 128.13 (L’), 128.08 (L), 127.94 (H), 127.90 (D, d, J = 8.8 Hz), 127.46 (H’), 127.24 (D’, d, J = 8.5 Hz), 125.94 (G’), 125.16 (G), 116.73 (C’, d, J = 22.9 Hz), 116.15 (C, d, J = 22.7 Hz), 114.41 (K), 114.31 (K’), 55.39 (N), 55.37 (N’); ^{19}F NMR (376.5 MHz, $CDCl_3$) δ –114.21 (minor), –114.61 (major); EI-HRMS: obtained *m/z* 321.11515 M^+ (expected *m/z* 321.11596 M^+);

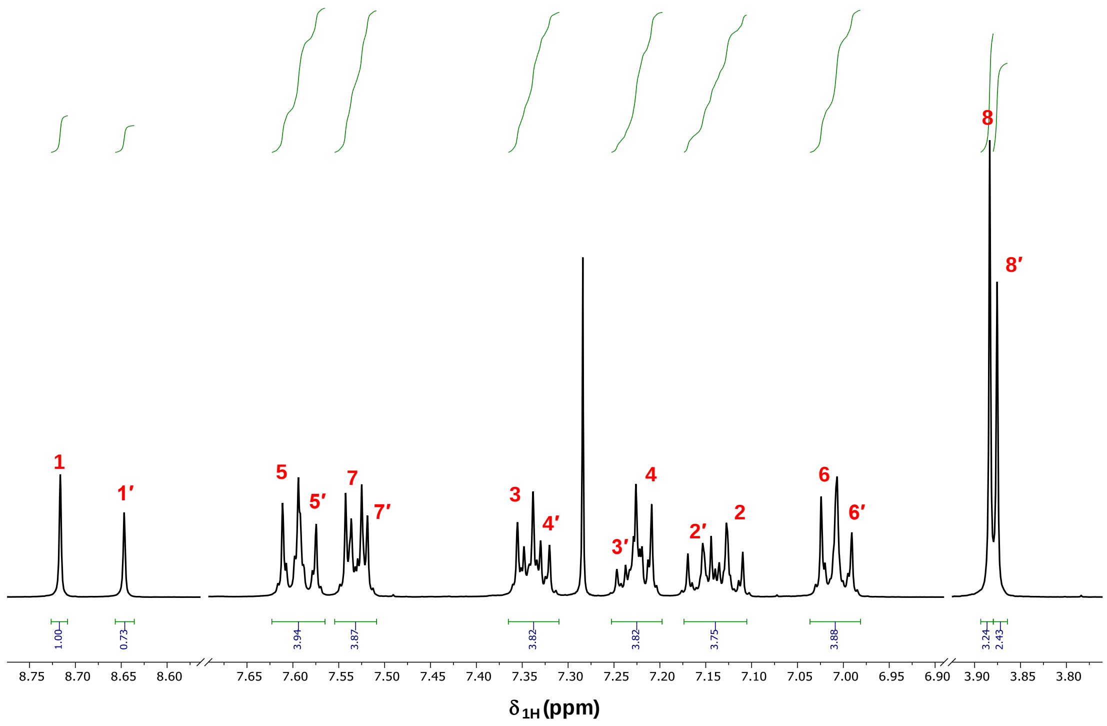
1
1′
5
5′
7
7′
3
4′
3′
4
2′
2
6
6′
8
8′
1.00
0.73
3.94
3.87
3.82
3.82
3.75
3.88
3.24
2.43
8.75 8.70 8.65 8.60 7.65 7.60 7.55 7.50 7.45 7.40 7.35 7.30 7.25 7.20 7.15 7.10 7.05 7.00 6.95 6.90 3.90 3.85 3.80
δ1H (ppm)

N-(3-nitro-[1,1-biphenyl]-4-yl)-N-(4-fluorophenyl)-formamide (1–j)

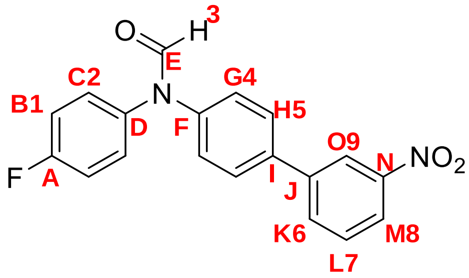

Prepared using the general palladium-mediated cross coupling procedure: *N*-(4-bromophenyl)-*N*-(4-fluorophenyl)formamide (251 mg, 0.85 mmol), $Pd(Ph_3P)_4$ (29.4 mg, 0.03 mmol), (3-nitrophenyl)boronic acid (198.5 mg, 1.19 mmol) DME (11 ml) and aq. Na_2CO_3 (2 M, 2 mL). Purified with preparative TLC (4:1 *n*-Hex: EtOAc) to yield **1–j** an orange solid (169.2 mg, 0.50 mmol, 59%).

υ_{max} (neat) /cm^{-1} (2920.94, 1692.45 (C=O), 1507.54, 1270.78, 838.63, 733.09; ^{1}H NMR (500 MHz, $CDCl_3$) δ 8.77 (3, s, 1H), 8.65 (3', s, 1H), 8.46 (9 and 9', m, 2H), 8.25 (7 and 7', m, 2H), 7.93 (8, s, 1H), 7.91 (8', s, 1H), 7.72 – 7.60 (6, 6', 4 and 4', m, 6H), 7.49 – 7.43 (5', m, 2H), 7.36 – 7.29 (2' and 5, m, 4H), 7.27 – 7.23 (2, m, 2H), 7.22 – 7.12 (1 and 1', m, 4H); ^{13}C NMR (126 MHz, $CDCl_3$) δ 161.75 (A, d, J = 248.4 Hz), 161.58 (E), 161.46 (E'), 161.29 (A', d, J = 247.5 Hz), 148.84 (N), 148.80 (N'), 142.00 (F), 141.88 (J), 141.46 (I), 140.09 (F'), 137.44 (J'), 137.34 (D, d, J = 3.3 Hz), 137.00 (I'), 135.18 (D', d, J = 3.0 Hz), 132.92(O'), 132.83 (O), 129.98 (M'), 129.87 (M), 128.59 (G'), 128.19 (C', d, J = 8.4 Hz), 127.97 (G), 127.63 (C, d, J = 8.5 Hz), 126.03 (H'), 125.04 (H), 122.49 (L), 122.29 (L'), 121.87 (K, K'), 116.92 (B', d, J = 22.9 Hz), 116.36 (B, d, J = 22.9 Hz); ^{19}F NMR (376.5 MHz, $CDCl_3$) δ –113.51 (major), –113.97 (minor); EI-HRMS: obtained *m/z* 336.08840 M$^+$ (expected *m/z* 336.08665 M$^+$).

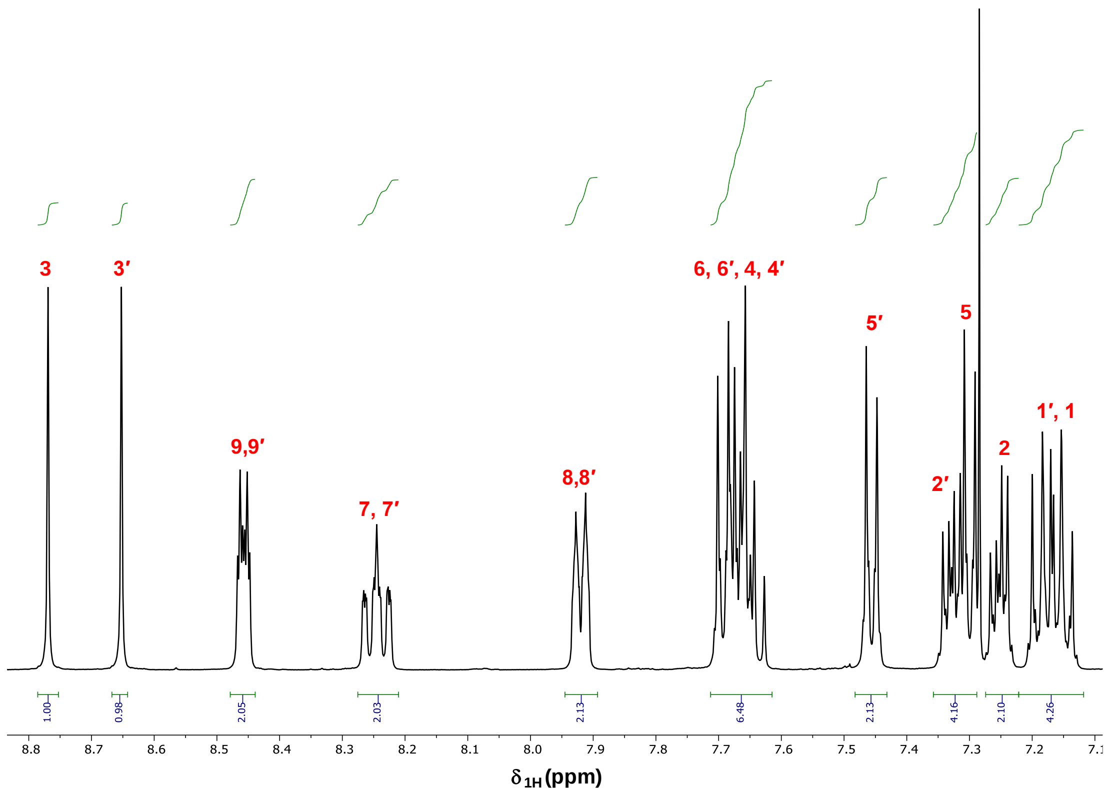
3
3′
9,9′
7, 7′
8,8′
6, 6′, 4, 4′
5′
5
2′
2
1′, 1
1.00
0.98
2.05
2.03
2.13
6.48
2.13
4.16
2.10
4.26
8.8
8.7
8.6
8.5
8.4
8.3
8.2
8.1
8.0
7.9
7.8
7.7
7.6
7.5
7.4
7.3
7.2
δ_{1H} (ppm)

N-(3-methoxy-[1,1-biphenyl]-4-yl)-N-(4-fluorophenyl)-formamide (1–k)

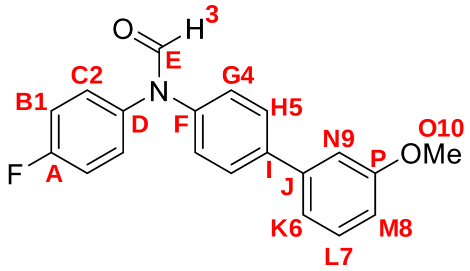

Prepared using the general palladium-mediated cross coupling procedure: $Pd(Ph_3P)_4$ (29.30 mg, 0.03 mmol), DME (11 mL), (3-methoxyphenyl)boronic acid (181.1 mg, 1.19 mmol), aq. Na_2CO_3 (2 M, 2 mL)and *N*-(4-bromophenyl)-*N*-(4-fluorophenyl)formamide (250 mg, 0.85 mmol). Purified with flash chromatography (4:1 *n*-Hex: EtOAc) to yield **1–k** a white solid (210.46 mg, 0.66 mmol, 77%).

υ_{max} (neat) /cm^{-1} 2979.42, 2940.91,1723.25 (C=O), 1608.80, 1508.96, 1479.01, 1263.65, 1213.73, 1138.14, 1032.60, 792.43; ^{1}H NMR (500 MHz, $CDCl_3$) δ 8.71 (3, s, 1H), 8.62 (3’, s, 1H), 7.63 – 7.58 (5 and 4’, m, 4H), 7.39 – 7.35 (7’, 5’ and 9, m, 4H), 7.34 – 7.29 (2, m, 2H), 7.23 – 7.20 (2’ and 4, m, 4H), 7.16 – 7.15 (6’, m, 1H), 7.14 – 7.12 (1’ and 1, m, 4H), 7.11 – 7.09 (9’, m, 1H), 6.94 – 6.89 (8 and 8’, m, 2H), 3.87 (10, s, 3H), 3.86 (10’, s, 3H); ^{13}C NMR (126 MHz, $CDCl_3$) δ 162.58 (E), 162.12 (E’), 160.61 (A’, d, J = 247.8 Hz), 161.14 (A, d, J = 247.0 Hz), 160.07 (P), 159.99 (P’), 141.75 (J’), 141.28 (J), 140.88 (I), 140.05 (F), 139.66 (I’), 138.94 (F’), 137.66 (D’, d, J = 3.2 Hz), 135.46 (D, d, J = 3.3 Hz), 129.99 (L), 129.86 (L’), 128.49 (H), 128.00 (C, d, J = 8.5 Hz), 127.97 (G), 127.37 (C’, d, J = 8.5 Hz), 125.84 (H’), 124.98 (G’), 119.60 (K’), 119.52 (K), 116.76 (B’, d, J = 22.9 Hz), 116.20 (B, d, J = 22.8 Hz), 113.00 (M), 112.96 (N), 112.92 (N’), 112.89 (N’), 55.35 (O), 55.32 (O’); ^{19}F NMR (376.5 MHz, $CDCl_3$) δ −114.04 (minor), −114.45 (major); EI-HRMS: obtained *m/z* 321.11480 M^+ (expected *m/z* 321.11596 M^+).

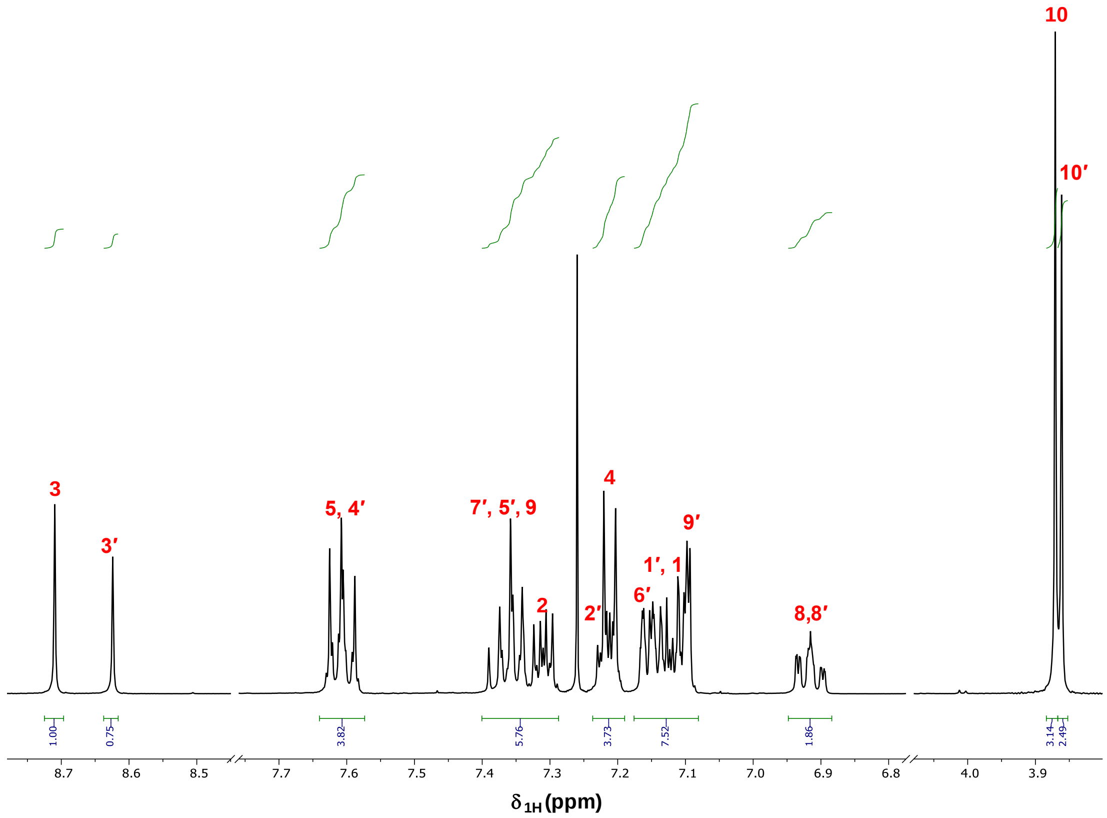
10
10′
3
3′
5, 4′
7′, 5′, 9
2
2′
4
6′
1′, 1
9′
8,8′
1.00
0.75
3.82
5.76
3.73
7.52
1.86
3.14
2.49
8.7
8.6
8.5
7.7
7.6
7.5
7.4
7.3
7.2
7.1
7.0
6.9
6.8
4.0
3.9
δ1H (ppm)

S8.3 Synthesis of 1–Me

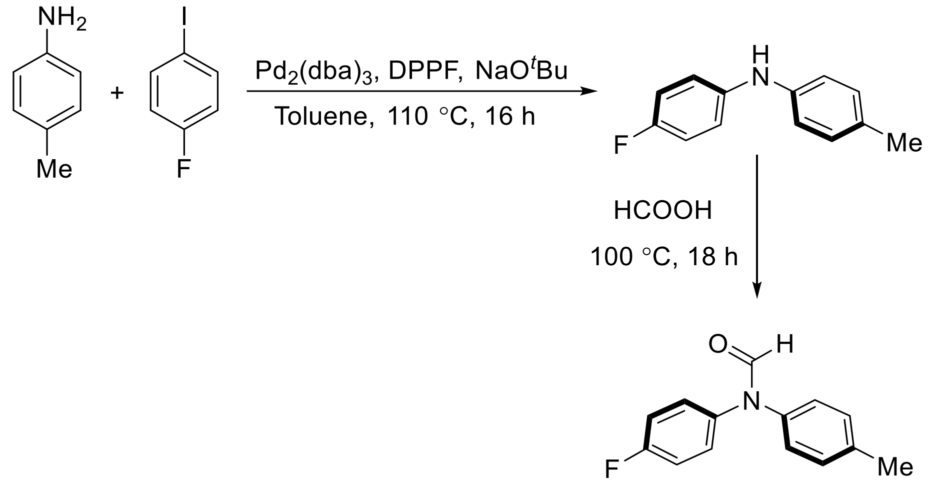

1–Me

1–Me was obtained *via* a two-step synthesis beginning with the palladium mediated coupling between *p*-toluidene and 4-fluoroiodobenzene. Formylation of the resulting secondary amine was then performed to yield **1–Me**.

N-(4-fluorophenyl)-*N*-(4-methylphenyl)formamide (1–Me)[2-3, 10]

p-Toluidene (1.00 g, 9.33 mmol), 4-fluoroiodobenzene (1.1 mL, 9.33 mmol), $Pd_2(dba)_3$ (171.4 mg, 0.19 mmol), 1,1′-ferrocenediyl-bis(diphenylphosphine) (DPPF) (155.8 mg, 0.28 mmol) and NaO*t*-But (1344 mg, 14 mmol) were added to a dry flask which was sealed before evacuating and nitrogen back filling three times. Toluene (36 mL) was added *via* syringe and the reaction mixture was heated at reflux for 48 h. The suspension was cooled to ambient temperature and filtered through kieselguhr, eluting with ethyl acetate (400 mL). The organics were washed with sat. aq. $NaHCO_3$ (2 x 250 mL) and brine (3 x 250 mL) and dried ($MgSO_4$) before concentration *in vacuo*. The product was carried through to the next step without further purification.

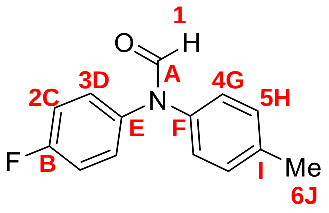

N-(4-Fluorophenyl)-4-methyl-aniline (~1500 mg, 2.7 mmol) was refluxed for 18 h in formic acid (25 mL). Formic acid was removed under reduced pressure and the crude product dissolved in ethyl acetate and washed with sat. aq. $NaHCO_3$ and brine. After being dried

over $MgSO_4$, the crude product was concentrated *in vacuo* to yield *N*-(4-fluorophenyl)-N-(4-methylphenyl)formamide as a light brown solid (1236.5 mg, 5.39 mmol, ~72%).

υ_{max} (neat) / cm^{-1} 2982.27, 1668.70 (C=O), 1504.45, 1323.55, 1226.57, 1053.99, 1012.60, 838.94; ^{1}H NMR (500 MHz, $CDCl_3$) δ 8.63 (1, s, 1H), 8.62 (1', s, 1H), 7.30 – 7.28 (3', m, 2H), 7.25 – 7.21 (5 and 5', m, 4H), 7.19 – 7.16 (3 and 4', m, 4H), 7.13 – 7.11 (2', m, 2H) 7.11 – 7.08 (2, m, 2H), 7.08 – 7.06 (4, m, 2H), 2.40 (6, s, 3H), 2.38 (6', s, 3H); ^{13}C NMR (126 MHz, $CDCl_3$) δ 161.74 (A), 161.53 (A'), 161.36 (B', d, J = 247.4 Hz), 160.88 (B, d, J = 246.7 Hz), 139.04 (I), 137.98 (E', d, J = 3.1 Hz), 137.30 (F), 137.00 (I'), 136.92 (F'), 135.79 (E, d, J = 3.1 Hz), 130.38 (H), 129.89 (H'), 127.58 (D, d, J = 8.4 Hz), 126.82 (D', d, J = 8.5 Hz), 125.77 (G'), 125.09 (G), 116.57 (C', d, J = 22.8 Hz), 115.99 (C, d, J = 22.7 Hz), 21.04 (J'), 20.95 (J); ^{19}F NMR (376.5 MHz, $CDCl_3$) δ –114.66 (minor), –114.99 (major); EI-HRMS: obtained *m/z* 229.08805 M^+ (expected *m/z* 229.08974 M^+)

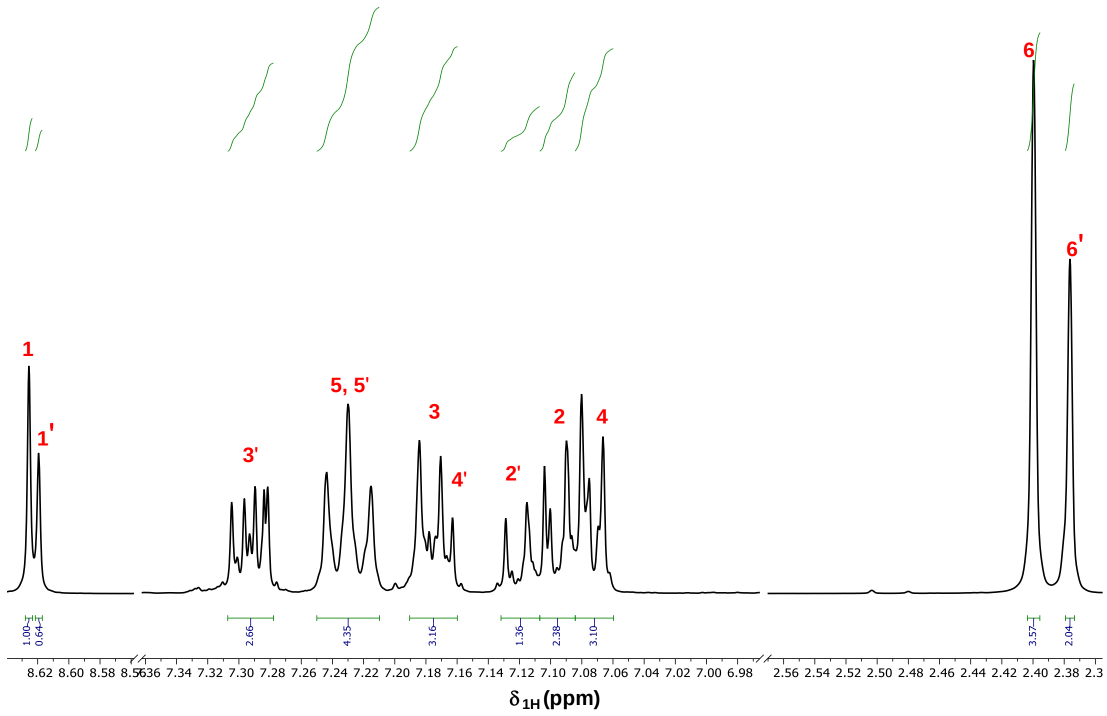

S8.4 General procedure for the Formylation of Anilines

N-(4-Fluorophenyl)-formamide

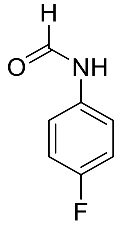

4-Fluoroaniline (256 µL, 2.7 mmol) was refluxed for 18 h in formic acid (15.25 mL). After quenching with sat. aq. $NaHCO_3$, the aqueous phase was extracted with EtOAc (3 x 15 mL) and concentrated *in vacuo* to yield *N*-(4-fluorophenyl)-formamide as a brown solid (342 mg, 2.46 mmol, 91%).

^{1}H NMR (400 MHz, $CDCl_3$) δ 8.57 (d, J = 11.4 Hz, 1H, trans), 8.37 (d, J = 1.6 Hz, 1H, cis), 7.90 (s, NH), 7.51 (m, 2H), 7.28 (s, NH), 7.03 (m, 2H); ^{13}C NMR (101 MHz, $CDCl_3$) δ 163.35', 160.47' (d, J = 244.8 Hz), 159.64 (d, J = 244.1 Hz), 159.62, 133.06 (d, J = 2.9 Hz), 132.89' (d, J = 2.9 Hz), 122.03 (d, J = 7.9 Hz), 121.14' (d, J = 8.2 Hz), 116.57' (d, J = 22.9 Hz), 115.77 (d, J = 22.5 Hz); EI-HRMS: obtained m/z 139.042796 M^+ (expected m/z 139.04279 M^+).

Characterization was consistent with existing literature.[11]

N-(4-Bromophenyl)-formamide

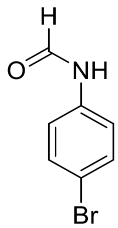

4-bromoaniline (10.01 g, 50 mmol) was refluxed for 18 h in formic acid (30 mL). Formic acid was removed under reduced pressure before ethyl acetate (110 mL) was added and the reaction mixture washed with sat. aq. $NaHCO_3$, brine, dried ($MgSO_4$) and concentrated in *vacuo* to yield crude *N*-(4-bromophenyl)-formamide as a brown solid which was carried to the next step without further purification.

^{1}H NMR (400 MHz, $CDCl_3$) δ 8.41 (d, J = 1.6 Hz, 1H), 7.51 (d, J = 8.8 Hz, 1H), 7.14 (s, 0H), 6.99 (d, J = 8.7 Hz, 1H).

Characterization was consistent with existing literature.[11]

S9. References for Section A

1. Mati, I. K. Molecular torsion balances for quantifying non-covalent interactions. The University of Edinburgh, 2013.

2. Mati, I. K.; Adam, C.; Cockroft, S. L., Seeing through solvent effects using molecular balances. *Chem. Sci.* **2013,** *4,* 3965-3972.

3. Muchowska, K. B.; Adam, C.; Mati, I. K.; Cockroft, S. L., Electrostatic Modulation of Aromatic Rings *via* Explicit Solvation of Substituents. *J. Am. Chem. Soc.* **2013,** *135* (27), 9976 - 9979.

4. Hansch, C.; Leo, A.; Taft, R. W., A survey of Hammett substituent constants and resonance and field parameters. *Chem. Rev.* **1991,** *91* (2), 165-95.

5. McDaniel, D. H.; Brown, H. C., An Extended Table of Hammett Substitutent Constants Based on the Ionization of Substituted Benzoic Acids. *J. Org. Chem.* **1958,** *23* (3), 420-427.

6. Hunter, C. A., Quantifying Intermolecular Interactions: Guidelines for the Molecular Recognition Toolbox. *Angew. Chem. Int. Ed.* **2004,** *43* (40), 5310 - 5324.

7. Fulmer, G. R.; Miller, A. J. M.; Sherden, N. H.; Gottlieb, H. E.; Nudelman, A.; Stoltz, B. M.; Bercaw, J. E.; Goldberg, K. I., NMR Chemical Shifts of Trace Impurities: Common Laboratory Solvents, Organics, and Gases in Deuterated Solvents Relevant to the Organometallic Chemist. *Organometallics* **2010,** *29* (9), 2176-2179.

8. Phillips, D. P.; Zhu, X.-F.; Lau, T. L.; He, X.; Yang, K.; Liu, H., Copper-catalyzed C–N coupling of amides and nitrogen-containing heterocycles in the presence of cesium fluoride. *Tetrahedron Lett.* **2009,** *50* (52), 7293-7296.

9. Leão Lana, E. J.; Carazza, F.; Aparacida de Oliveira, R., Synthesis of 2-Aryl- and 2-Heteroaryl-3,5-dimethoxy-1,4-benzoquinones Involving Pd-Catalyzed Cross-Coupling of (2,3,4,6-Tetramethoxyphenyl)boronic Acid. *Helvetica Chimica Acta* **2004,** *87* (7), 1825-1831.

10. Dominelli-Whiteley, N.; Brown, J. J.; Muchowska, K. B.; Mati, I. K.; Adam, C.; Hubbard, T. A.; Elmi, A.; Brown, A. J.; Bell, I. A. W.; Cockroft, S. L., Strong Short-Range Cooperativity in Hydrogen-Bond Chains. *Angew. Chem. Int. Ed.* **2017,** *56* (26), 7658-7662.

11. Hosseini-Sarvari, M.; Sharghi, H., ZnO as a New Catalyst for *N*-Formylation of Amines under Solvent-Free Conditions. *J. Org. Chem.* **2006,** *71* (17), 6652-6654.

Section B: Pyridine derivative data

S10. Computational Methods and Data

S10.1 Electrostatic Potential Surface and Slice Calculations

Minimized geometries, electrostatic potentials and average local ionization potentials of the X-substituted pyridine derivatives were calculated at the DFT/B3LYP/6–31G* level using Spartan '14 (Figures S43-S59 and S60-S76).

ESP_N values were measured on the ESP surface over the pyridine nitrogen atom (Figure S42, Table S14). ESP surfaces and slices are given for series **2–X** in Figures S43-S59.

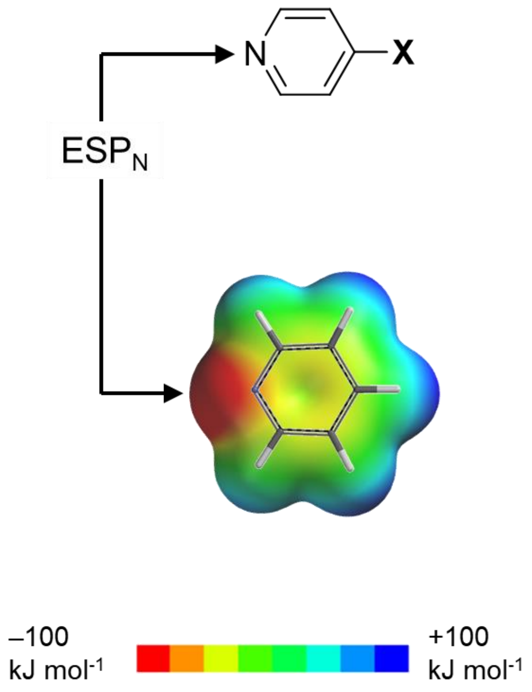

Figure S42. DFT/B3LYP/6–31G* electrostatic potential (ESP) surface used to model the electrostatic properties of pyridine derivatives where X = H. The ESP values over the pyridine nitrogen atom were taken at the 0.002 electron/Bohr^3 isosurface as indicated.

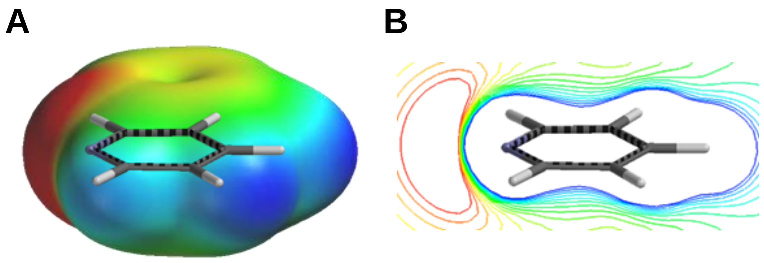

Figure S43. ESP surface (**A**) and slice (**B**) of **2–H**. Calculated using DFT/B3LYP/6–31G*. Scaled from –100 kJ mol^{-1} (red) to +100 kJ mol^{-1} (blue) on the 0.002 electron/Bohr^3 isosurface.

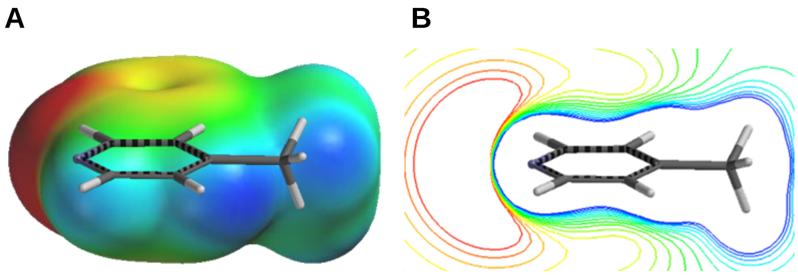

Figure S44. ESP surface (**A**) and slice (**B**) of **2–Me**. Calculated using DFT/B3LYP/6–31G*. Scaled from –100 kJ mol^{-1} (red) to +100 kJ mol^{-1} (blue) on the 0.002 electron/Bohr^3 isosurface.

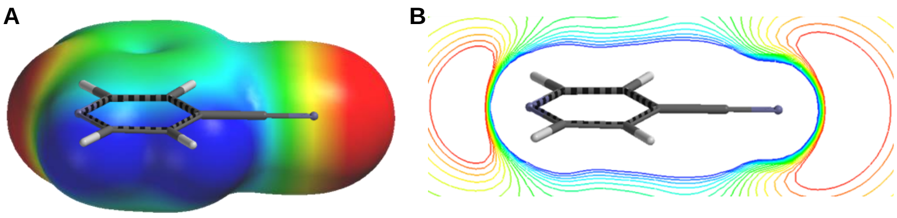

Figure S45. ESP surface (**A**) and slice (**B**) of **2–CN**. Calculated using DFT/B3LYP/6–31G*. Scaled from –100 kJ mol^{-1} (red) to +100 kJ mol^{-1} (blue) on the 0.002 electron/Bohr^3 isosurface.

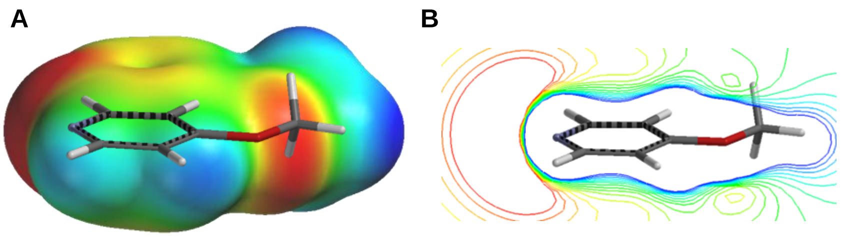

Figure S46. ESP surface (**A**) and slice (**B**) of **2–OMe**. Calculated using DFT/B3LYP/6–31G*. Scaled from –100 kJ mol^{-1} (red) to +100 kJ mol^{-1} (blue) on the 0.002 electron/Bohr^3 isosurface.

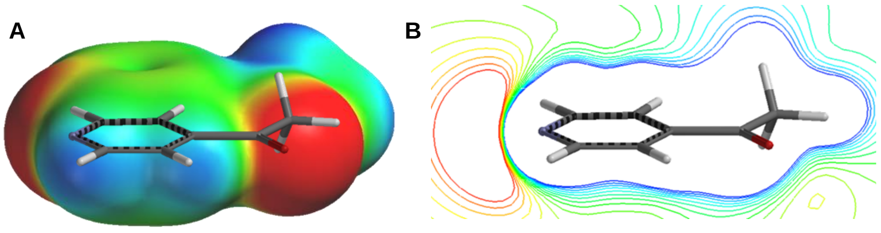

Figure S4.: ESP surface (**A**) and slice (**B**) of **2–COMe**. Calculated using DFT/B3LYP/6–31G*. Scaled from –100 kJ mol^{-1} (red) to +100 kJ mol^{-1} (blue) on the 0.002 electron/Bohr^3 isosurface.

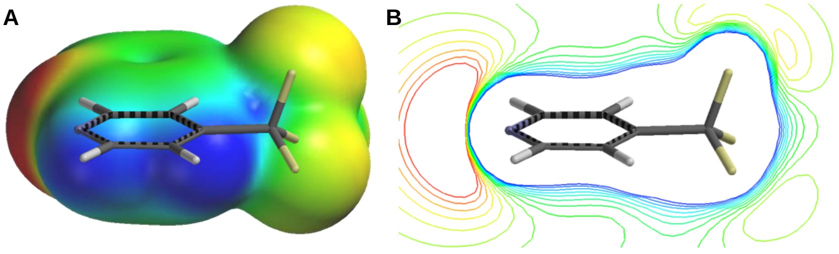

Figure S48. ESP surface (**A**) and slice (**B**) of **2–CF$_3$**. Calculated using DFT/B3LYP/6–31G*. Scaled from –100 kJ mol^{-1} (red) to +100 kJ mol^{-1} (blue) on the 0.002 electron/Bohr^3 isosurface.

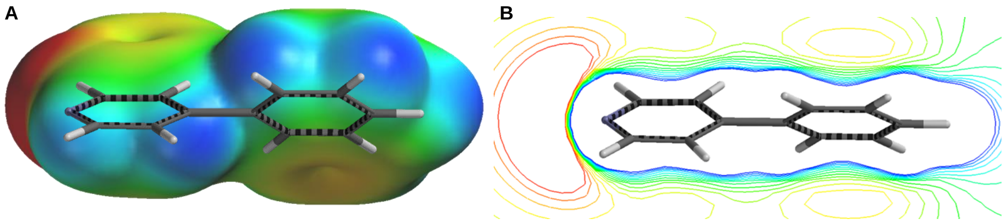

Figure S49. ESP surface (**A**) and slice (**B**) of **2–Ph**. Calculated using DFT/B3LYP/6–31G*. Scaled from –100 kJ mol^{-1} (red) to +100 kJ mol^{-1} (blue) on the 0.002 electron/Bohr^3 isosurface.

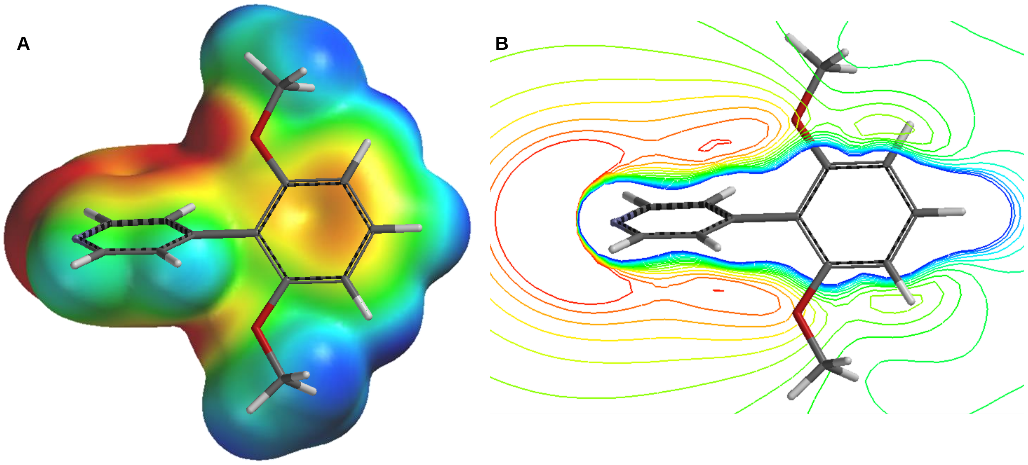

Figure S50. ESP surface (**A**) and slice (**B**) of **2**–**a**. Calculated using DFT/B3LYP/6–31G*. Scaled from –100 kJ mol^{-1} (red) to +100 kJ mol^{-1} (blue) on the 0.002 electron/Bohr^3 isosurface.

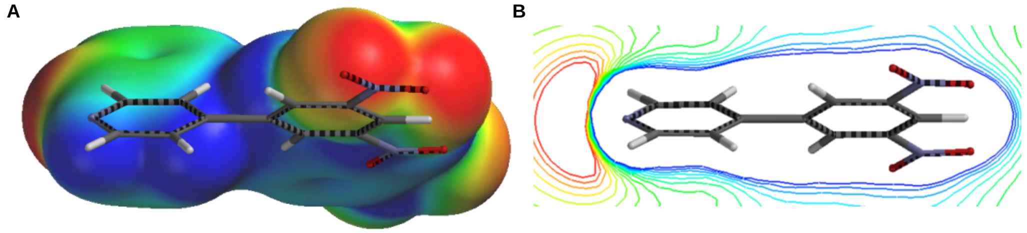

Figure S51. ESP surface (**A**) and slice (**B**) of **2**–**e**. Calculated using DFT/B3LYP/6–31G*. Scaled from –100 kJ mol^{-1} (red) to +100 kJ mol^{-1} (blue) on the 0.002 electron/Bohr^3 isosurface.

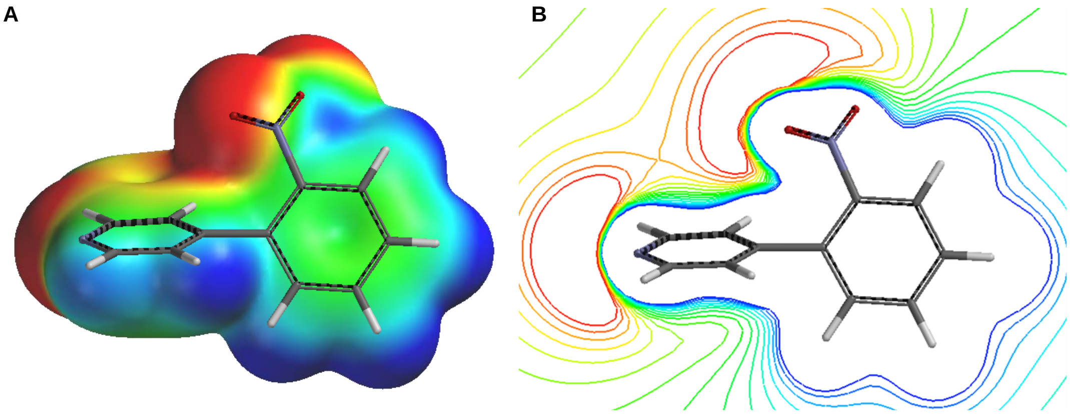

Figure S52. ESP surface (**A**) and slice (**B**) of **2–f**. Calculated using DFT/B3LYP/6–31G*. Scaled from –100 kJ mol^{-1} (red) to +100 kJ mol^{-1} (blue) on the 0.002 electron/Bohr^3 isosurface.

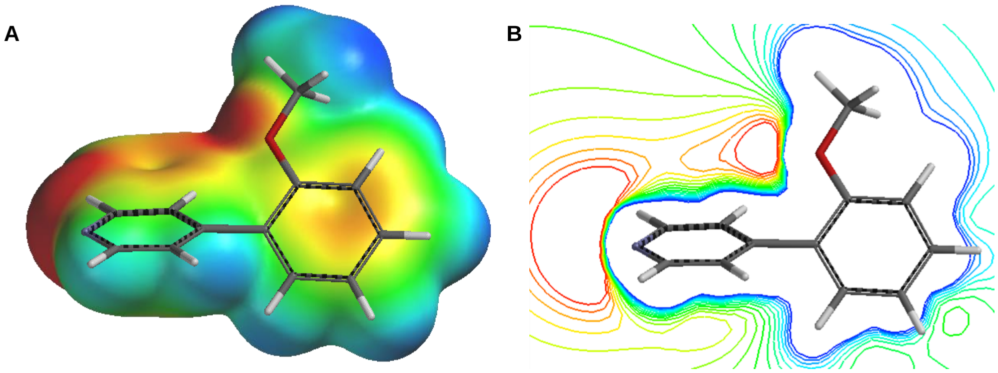

Figure S53. ESP surface (**A**) and slice (**B**) of **2–g**. Calculated using DFT/B3LYP/6–31G*. Scaled from –100 kJ mol^{-1} (red) to +100 kJ mol^{-1} (blue) on the 0.002 electron/Bohr^3 isosurface.

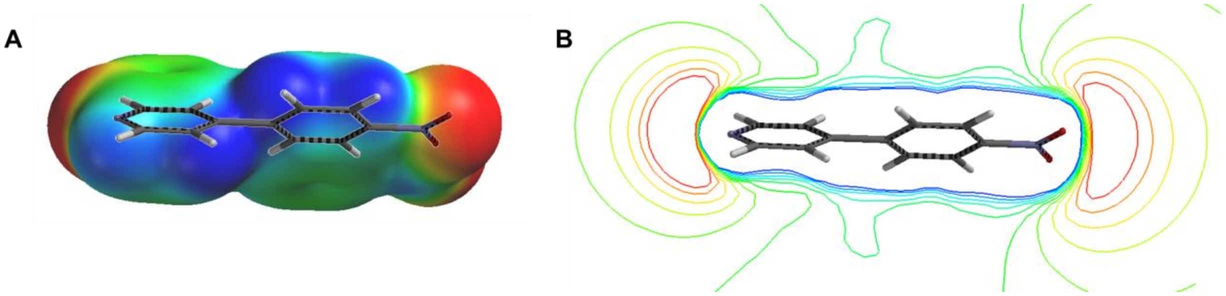

Figure S54. ESP surface (**A**) and slice (**B**) of **2–h**. Calculated using DFT/B3LYP/6–31G*. Scaled from –100 kJ mol^{-1} (red) to +100 kJ mol^{-1} (blue) on the 0.002 electron/Bohr^3 isosurface.

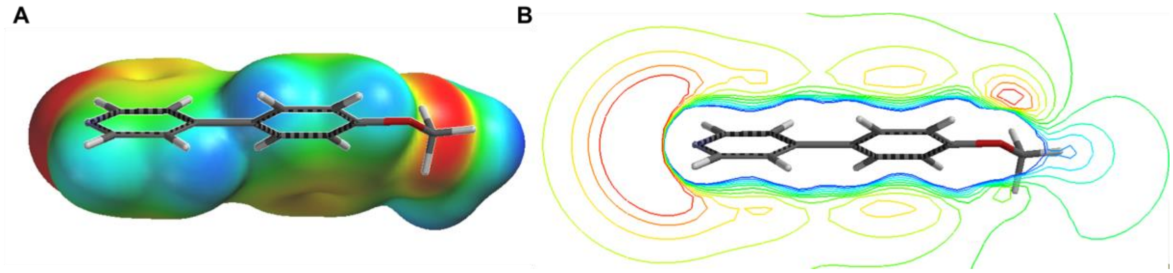

Figure S55. ESP surface (**A**) and slice (**B**) of **2–i**. Calculated using DFT/B3LYP/6–31G*. Scaled from –100 kJ mol^{-1} (red) to +100 kJ mol^{-1} (blue) on the 0.002 electron/Bohr^3 isosurface.

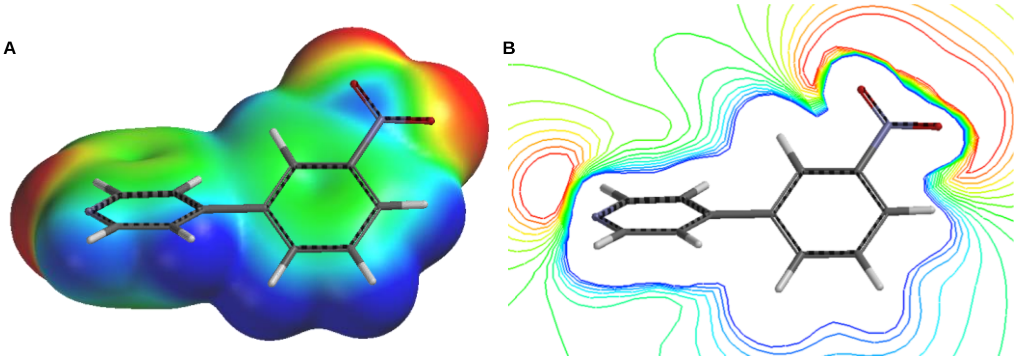

Figure S56. ESP surface (**A**) and slice (**B**) of **2–j**. Calculated using DFT/B3LYP/6–31G*. Scaled from –100 kJ mol^{-1} (red) to +100 kJ mol^{-1} (blue) on the 0.002 electron/Bohr^3 isosurface.

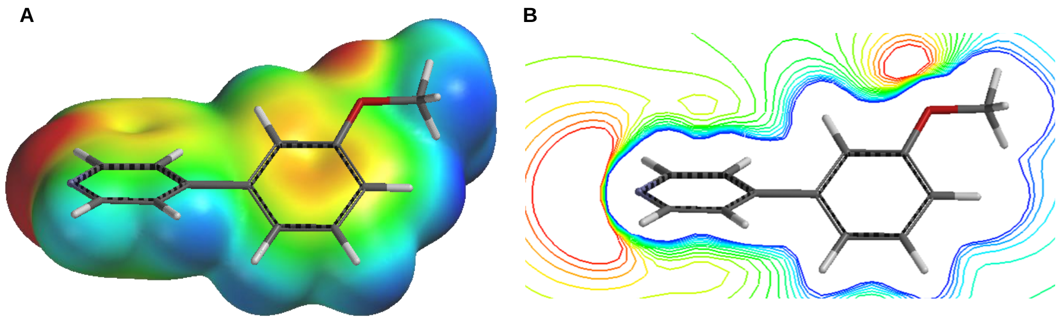

Figure S57. ESP surface (**A**) and slice (**B**) of **2–k**. Calculated using DFT/B3LYP/6–31G*. Scaled from –100 kJ mol^{-1} (red) to +100 kJ mol^{-1} (blue) on the 0.002 electron/Bohr^3 isosurface.

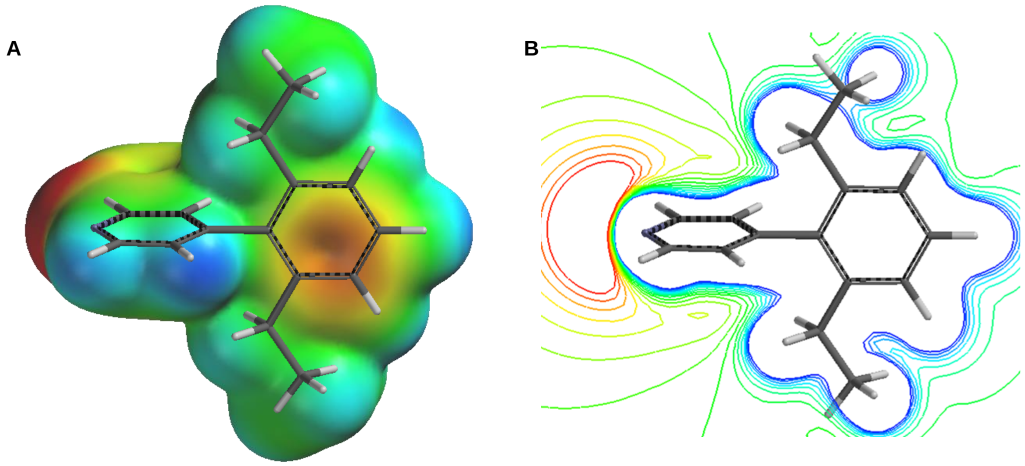

Figure S58. ESP surface (**A**) and slice (**B**) of **2–l**. Calculated using DFT/B3LYP/6–31G*. Scaled from –100 kJ mol^{-1} (red) to +100 kJ mol^{-1} (blue) on the 0.002 electron/Bohr^{3} isosurface.

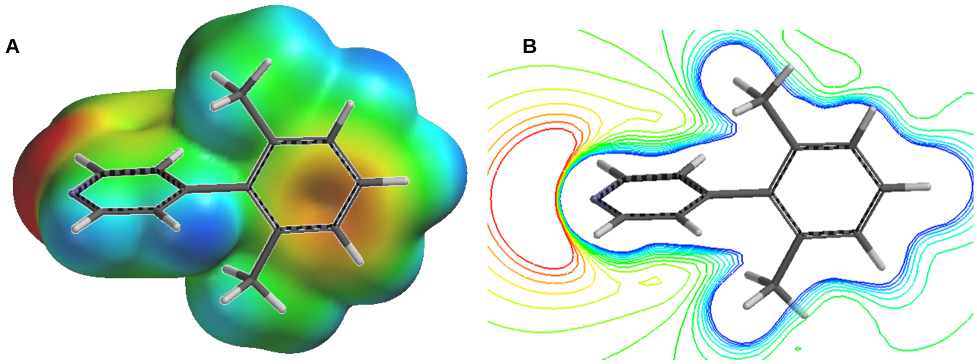

Figure S59. ESP surface (**A**) and slice (**B**) of **2–m**. Calculated using DFT/B3LYP/6–31G*. Scaled from –100 kJ mol^{-1} (red) to +100 kJ mol^{-1} (blue) on the 0.002 electron/Bohr^{3} isosurface.

S10.2 Ionization Energy Surface calculations

Ionization energy surfaces were calculated at the DFT/B3LYP/6–31G* level of theory for the minimized structures using Spartan '14. IE_N values were measured on the IE surface over the pyridine nitrogen atom (Figure S60, Table S14).

IE surfaces are given for series **2–X** in Figures S60 to S76.

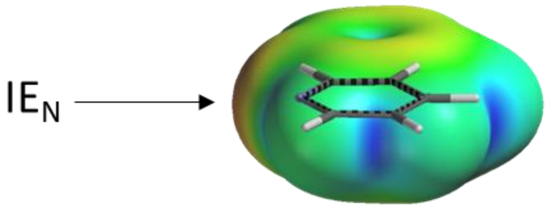

Figure S60. IE surface of **2–H**. Calculated using DFT/B3LYP/6–31G*. Scaled from +7.5 eV to +15 eV on the 0.002 electron/Bohr^3 isosurface.

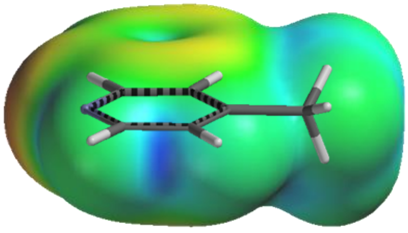

Figure S61. IE surface of **2–Me**. Calculated using DFT/B3LYP/6–31G*. Scaled from +7.5 eV to +15 eV on the 0.002 electron/Bohr^3 isosurface.

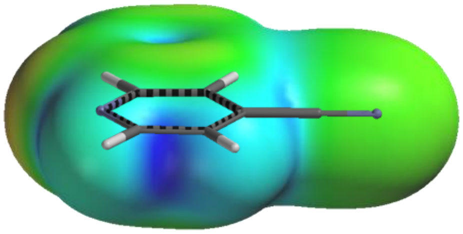

Figure S62. IE surface of **2–CN**. Calculated using DFT/B3LYP/6–31G*. Scaled from +7.5 eV to +15 eV on the 0.002 electron/Bohr^3 isosurface.

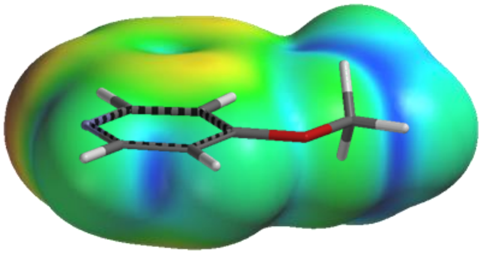

Figure S63. IE surface of **2–OMe**. Calculated using DFT/B3LYP/6–31G*. Scaled from +7.5 eV to +15 eV on the 0.002 electron/Bohr^3 isosurface.

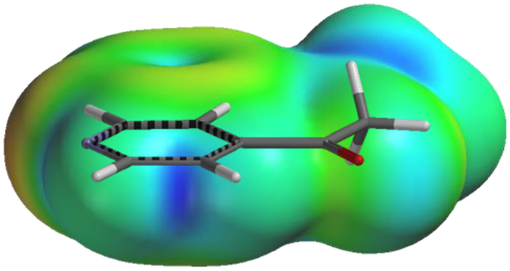

Figure S64. IE surface of **2–COMe**. Calculated using DFT/B3LYP/6–31G*. Scaled from +7.5 eV to +15 eV on the 0.002 electron/Bohr^3 isosurface.

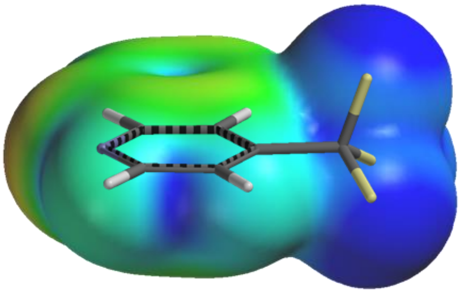

Figure S65. IE surface of **2–CF$_3$**. Calculated using DFT/B3LYP/6–31G*. Scaled from +7.5 eV to +15 eV on the 0.002 electron/Bohr^3 isosurface.

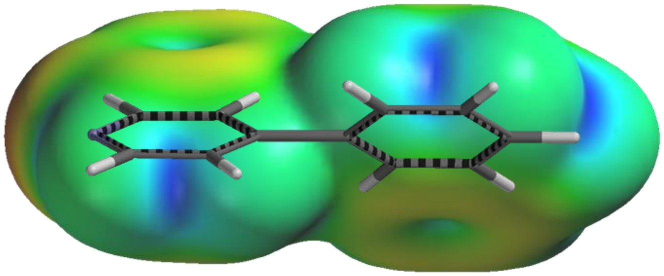

Figure S66. IE surface of **2–Ph**. Calculated using DFT/B3LYP/6–31G*. Scaled from +7.5 eV to +15 eV on the 0.002 electron/Bohr^3 isosurface.

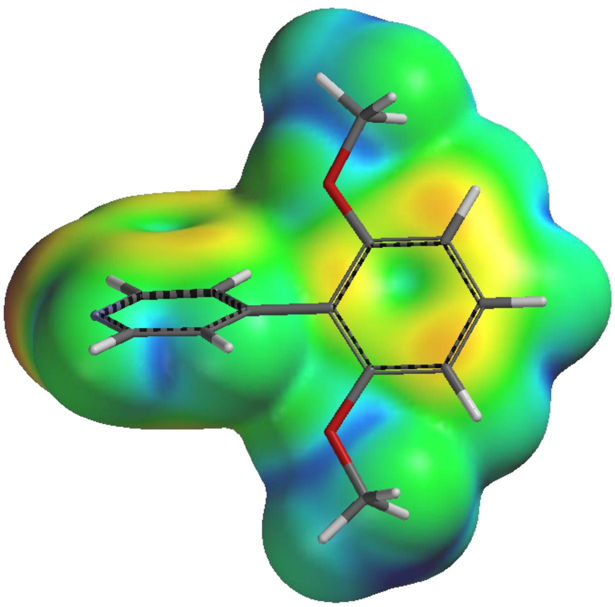

Figure S67. IE surface of **2–a**. Calculated using DFT/B3LYP/6–31G*. Scaled from +7.5 eV to +15 eV on the 0.002 electron/Bohr^3 isosurface.

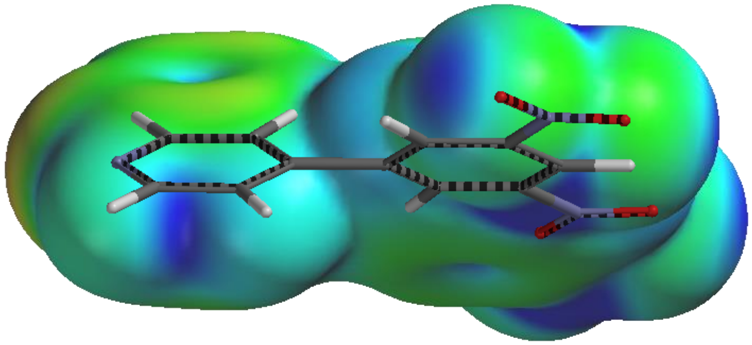

Figure S68. IE surface of **2–e**. Calculated using DFT/B3LYP/6–31G*. Scaled from +7.5 eV to +15 eV on the 0.002 electron/Bohr^3 isosurface.

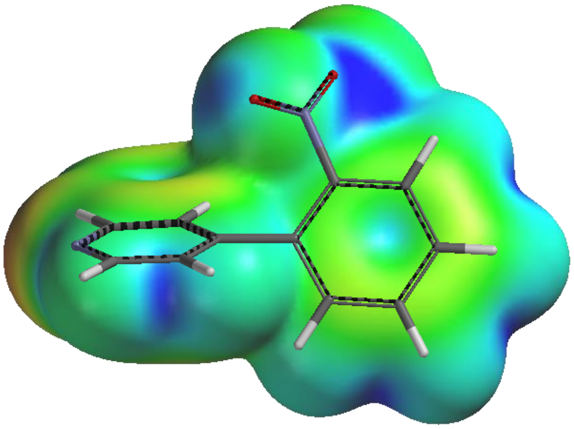

Figure S69. IE surface of **2–f**. Calculated using DFT/B3LYP/6–31G*. Scaled from +7.5 eV to +15 eV on the 0.002 electron/Bohr^3 isosurface.

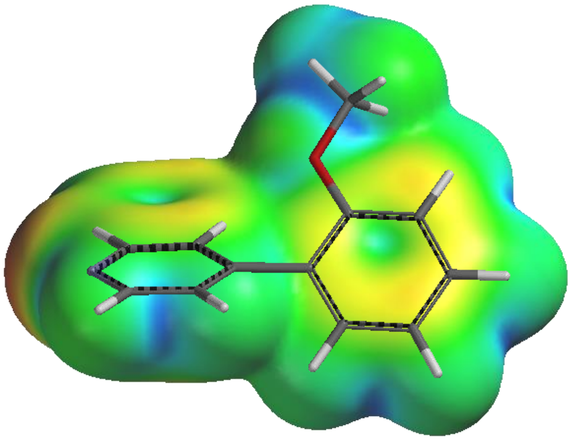

Figure S70. IE surface of **2–g**. Calculated using DFT/B3LYP/6–31G*. Scaled from +7.5 eV to +15 eV on the 0.002 electron/Bohr^3 isosurface.

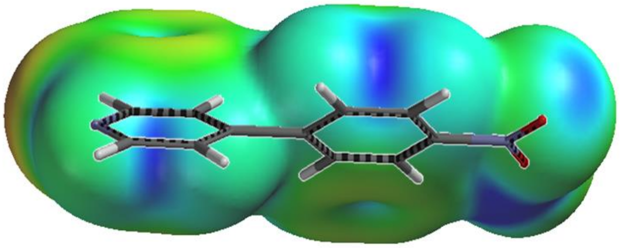

Figure S71. IE surface of **2–h**. Calculated using DFT/B3LYP/6–31G*. Scaled from +7.5 eV to +15 eV on the 0.002 electron/Bohr^3 isosurface.

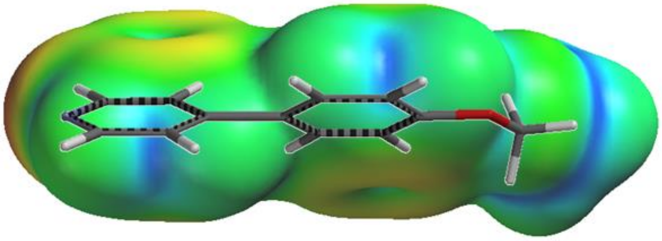

Figure S72. IE surface of **2–i**. Calculated using DFT/B3LYP/6–31G*. Scaled from +7.5 eV to +15 eV on the 0.002 electron/Bohr^3 isosurface.

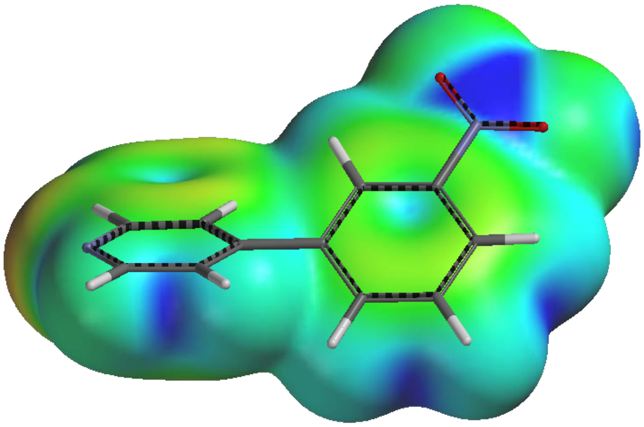

Figure S73. IE surface of **2–j**. Calculated using DFT/B3LYP/6–31G*. Scaled from +7.5 eV to +15 eV on the 0.002 electron/Bohr^3 isosurface.

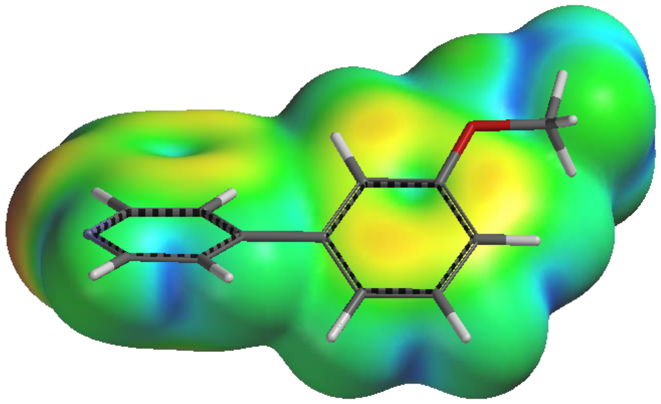

Figure S74. IE surface of **2–k**. Calculated using DFT/B3LYP/6–31G*. Scaled from +7.5 eV to +15 eV on the 0.002 electron/Bohr^3 isosurface.

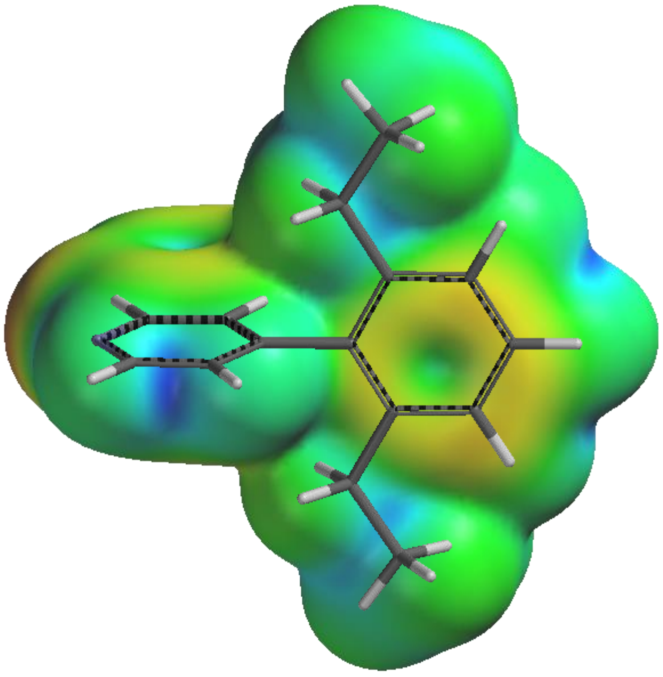

Figure S75. IE surface of **2–l**. Calculated using DFT/B3LYP/6–31G*. Scaled from +7.5 eV to +15 eV on the 0.002 electron/Bohr^3 isosurface.

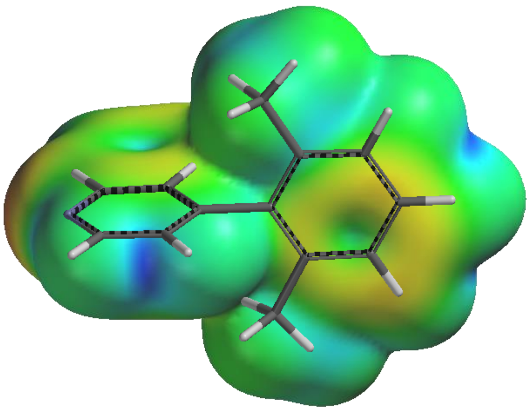

Figure S76. IE surface of **2–m**. Calculated using DFT/B3LYP/6–31G*. Scaled from +7.5 eV to +15 eV on the 0.002 electron/Bohr^3 isosurface.

Table S14. ESP_N and IE_N values of compounds in the **2–X** series. All structures and surfaces (from which ESP_N and IE_N values were calculated) were minimized and calculated using DFT/B3LYP/6–31G* in Spartan '14.

Compound	ESP_N / kJ mol^{-1}	IE_N / kJ mol^{-1}
**2–H**	–192.2	8.12
**2–OMe**	–203.5	7.95
**2–Me**	–199.2	8.02
**2–Ph**	–195.3	8.09
**2–CN**	–148.3	8.80
**2–CF_3**	–164.0	8.62
**2–NO_2**	–164.0	8.89
**2–$COCH_3$**	–172.4	8.43
**2–a**	–217.4	7.74
**2–e**	–156.0	8.23
**2–f**	–186.4	8.22
**2–g**	–205.6	7.92
**2–h**	–168.4	8.47
**2–i**	–200.9	7.99
**2–j**	–174.4	8.40
**2–k**	–199.6	8.03
**2–l**	–192.6	8.09
**2–m**	–192.4	8.11

S11. Determination of Rate Constants (k_X) and Error Analysis

NMR tubes and precision glassware used were dried under vacuum for at least 24 hours prior to the experiment. ^{1}H NMR spectra were recorded using a Bruker Ultrashield 400 MHz spectrometer at 298 K with a BBFO+ probe. The internal standard used for kinetic studies was acetone-d_6 (δ = 2.05 ppm).[4] Prior to performing kinetics experiments, all pyridine derivatives were dried under vacuum for a minimum of 16 h. Each experimental was run in duplicate to ensure the result was reproducible.

Stock solutions of pyridine derivatives (20 – 100 mM) and methyl iodide (1 M) were each prepared in acetone-d_6 and were prepared fresh for each experiment. Equal volumes of each (0.5 mL) were added to a vacuum dried NMR tube. A ^{1}H NMR spectrum taken as soon as possible after addition of methyl iodide to the pyridine derivative and then every 30 minutes for 6 hours. Each reaction was run in duplicate and the rate constants from each run, together with the average are given in Table S15.

The integrals of the signals corresponding to the protons *ortho* to the pyridine nitrogen atom of both the starting material and the *N*-methylated product (2,6 and 2,6 protons respectively) were determined at thirty-minute intervals. A representative example of this is given in Figure S77 for pyridine.

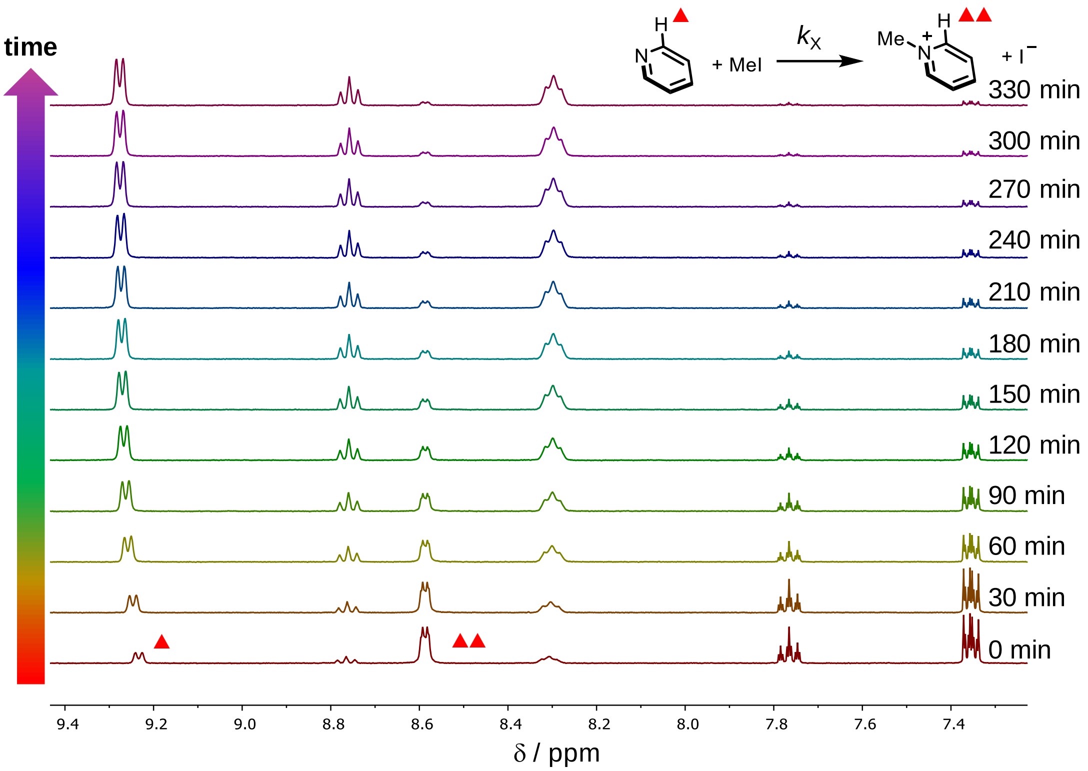

Figure S77. *N*-Methylation of **2–H** was monitored by ^{1}H NMR spectra (acetone-d_6, 400 MHz, 298 K) over five hours and 30 minutes with the 2,6 and 2,6 signals denoted by one and two red triangles respectively.

Through division of the 2,6 integral with the sum of these integrals, the change in concentration of the starting material, [sm], over the time of the experiment was determined. As an excess of methyl iodide was used, the reaction was under *pseudo* first order conditions thus allowing the experimental data to be fitted to this rate equation (Equation S6) to obtain the experimental rate constant, k_X, through linear regression (Figure S78).

$$[sm] = [sm]_0 \times e^{-k_X t} \qquad \textbf{Equation S6}$$

Where t is time and $[sm]_0$ is the concentration of the starting material at the beginning of the experiment, i.e. when t = 0.

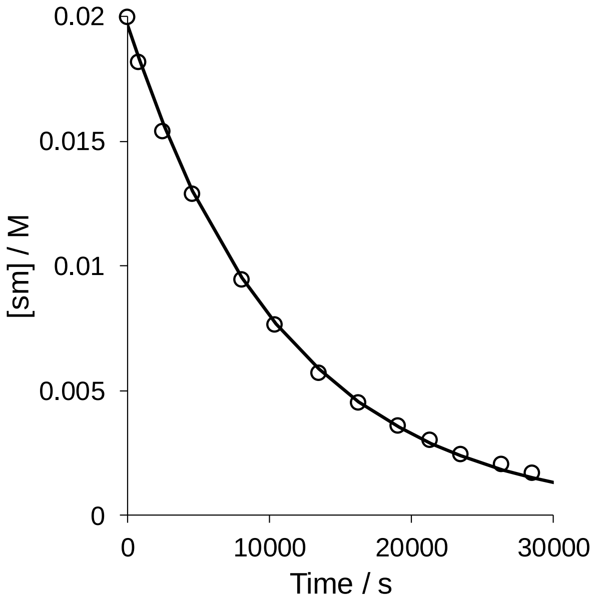

Figure S78. The experimental change in the starting material concentration, [sm], over time (hollow circles) with the fit of this data to the *pseudo* first order rate equation, **Equation S6**, (solid line) for **2–H** (20 mM, acetone-d_6, 400 MHz, 298 K).

The error in the rate constant, δk_X, is given as the standard deviation as calculated by Excel 2016 (*via* Equation S7) of the two experimental that comprise the average rate constant for each compound. The values of k_X for both experiments, the average k_X and δk_X are given in Table S15.

$$\delta k_X = \sqrt{\frac{\Sigma\left(k_x - \overline{k_X}\right)^2}{n}} \qquad \textbf{Equation S7}$$

Where k_X is the value of each experiment, $\overline{k_X}$ is the average k_X value and n is the number of values.

Table S15. Rate constants (k_X) of all pyridine derivatives studies measured in acetone-d_6 (400 MHz, 298 K). The values of each experiment (k_X (1) and (2)) and their average are given together with their error (δk_X). All values of k_X and δk_X were in x10^{-5} s^{-1}.

	k_X (1)	k_X (2)	Average k_X	δkX
**2–H**	9.55	8.98	9.26	0.40
**2–OMe**	25.20	24.73	24.97	0.33
**2–Me**	18.13	18.83	18.48	0.49
**2–Ph**	10.65	10.26	10.46	0.28
**2–CN**	0.41	0.34	0.37	0.05
**2–CF_3**	0.58	0.61	0.60	0.01
**2–$COCH_3$**	2.29	2.13	2.21	0.11
**2–a**	23.71	23.03	23.37	0.48
**2–e**	2.97	3.00	2.98	0.02
**2–f**	5.22	5.37	5.29	0.11
**2–g**	15.48	14.96	15.22	0.37
**2–h**	3.37	3.41	3.39	0.03
**2–i**	15.39	15.02	15.21	0.27
**2–j**	3.57	3.16	3.37	0.29
**2–k**	10.27	10.71	10.49	0.31
**2–l**	8.88	8.95	8.92	0.05
**2–m**	9.66	9.55	9.61	0.08

Verification that the reaction was indeed under *pseudo* first order conditions were performed by obtaining rate constants at varying concentrations of compounds whose experimental behavior span the extremes observed (Figure S79).

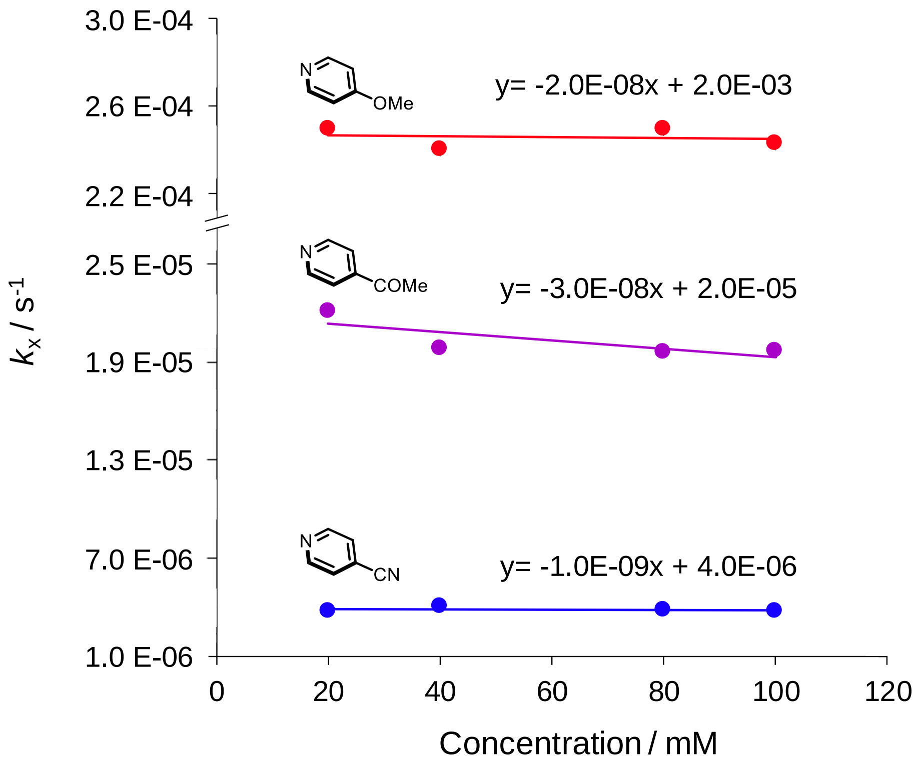

Figure S79. Rate constants, k_x, of **2–OMe**, **2–COMe** and **2–CN** at 20 mM, 40 mM, 80 mM and 100 mM pyridine concentration. Error bars are smaller than the data points. Measured in acetone-d_6 (400 MHz, 298 K).

A Hammett-style analysis using the relationship $-\log_{10}(k_X/k_H)$ was performed on the experimental rate constants, k_X, measured in in acetone-d_6 at 400 MHz, 298 K (Table S16). Errors in $-\log_{10}(k_X/k_H)$, $\delta\log_{10}(k_X/k_H)$, were calculated *via* a propagation of errors from k_X values (Equation S8). The values of $\delta k_{X/H}$ were determined from the standard deviation of the two $k_{X/H}$ values used to determine the mean $k_{X/H}$ values quoted in Table S16.

$$\delta log_{10}\left(\frac{k_X}{k_H}\right) = \sqrt{\left(\frac{\delta k_X}{k_X}\right)^2 + \left(\frac{\delta k_H}{k_H}\right)^2} \qquad \textbf{Equation S8}$$

Table S16. Rate constants (k_X) of all pyridine derivatives studies measured in acetone-d_6 (400 MHz, 298 K), values averaged over two experiments and their $-\log_{10}(k_X/k_H)$ and associated errors.

	$-\log_{10}(k_X/k_H)$	$\delta\log_{10}(k_X/k_H)$
**2–H**	0.00	0.06
**2–OMe**	–0.43	0.05
**2–Me**	–0.30	0.05
**2–Ph**	–0.05	0.05
**2–CN**	+1.39	0.15
**2–CF_3**	+1.19	0.05
**2–$COCH_3$**	+0.62	0.07
**2–a**	–0.40	0.05
**2–e**	+0.49	0.04
**2–f**	+0.24	0.05
**2–g**	–0.22	0.05
**2–h**	+0.44	0.04
**2–i**	–0.22	0.05
**2–j**	+0.44	0.10
**2–k**	–0.05	0.05
**2–l**	+0.02	0.04
**2–m**	–0.02	0.04

During transferability analysis of $\sigma_{p(conf)}$, $-\log_{10}(K_X/K_H)$ and $-\log_{10}(k_X/k_H)$ were correlated against each other with the former being obtained in eleven solvents: : acetone-d_6, acetonitrile-d_3, benzene-d_6, chloroform-d, DCM-d_2, diethyl ether, DMSO-d_6, ethanol, ethyl acetate, methanol-d_4 and THF-d_8. These plots are given in Figures S80 and S81.

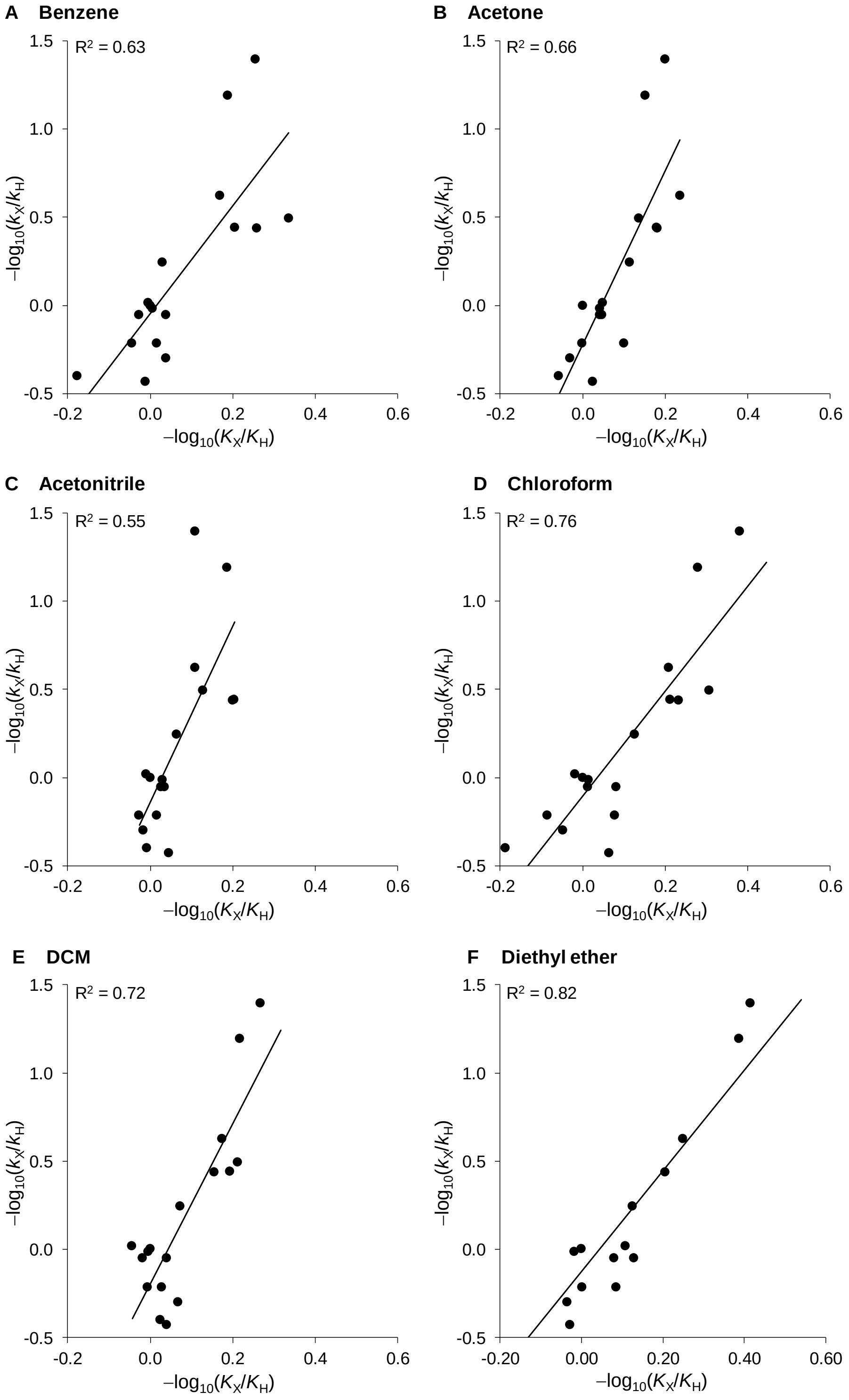

Figure S80. Kinetic data for *N*-methylation, $-\log_{10}(k_X/k_H)$ obtained in acetone-d_6 plotted against the values of $-\log_{10}(K_X/K_H)$ from the **1**–**X** series of molecular balances obtained in **(A)** benzene-d_6, **(B)** acetone-d_6, **(C)** acetonitrile-d_3, **(D)** chloroform-d_1, **(E)** DCM-d_2 and **(F)** diethyl ether.

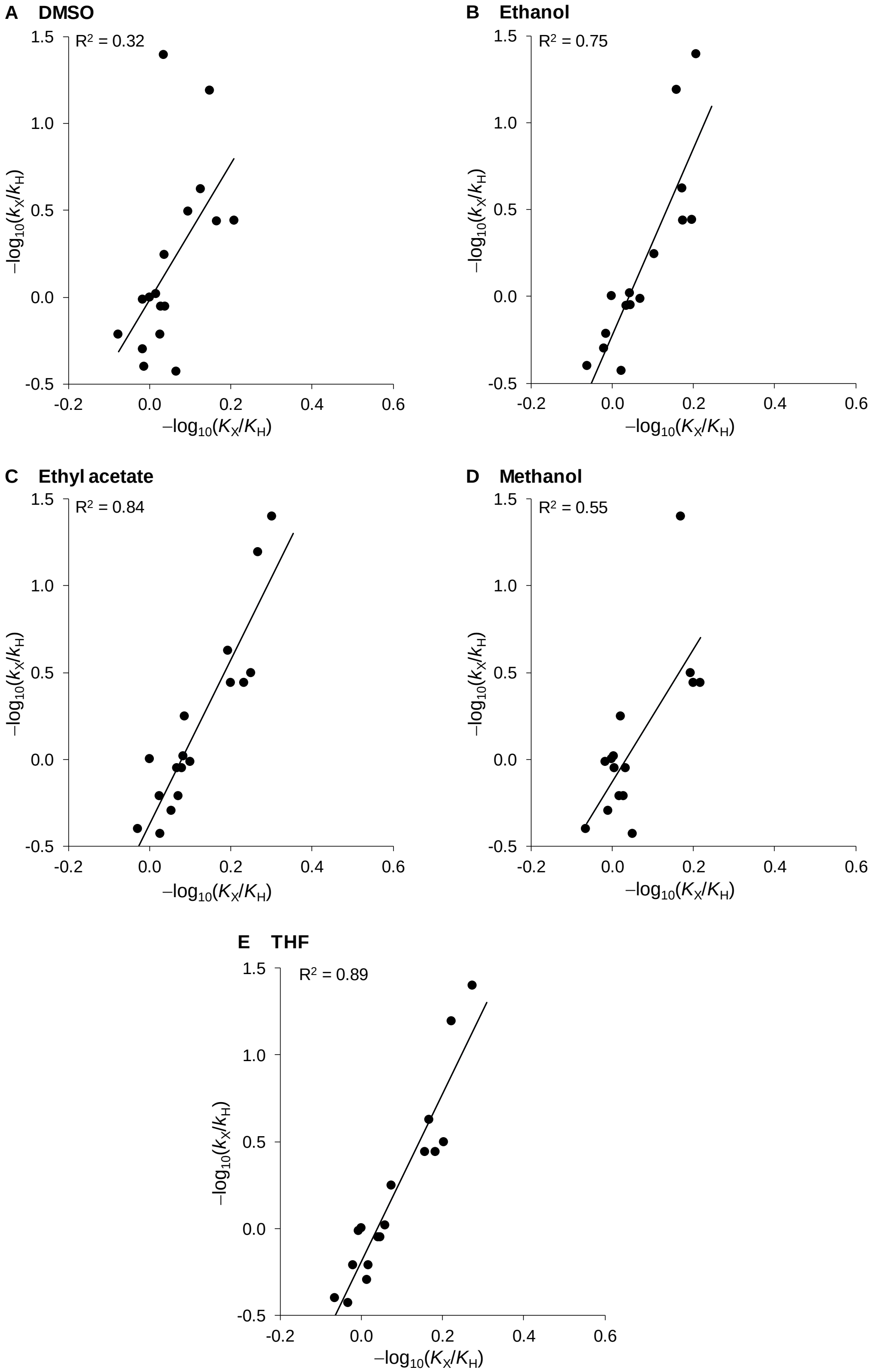

Figure S81. Kinetic data for *N*-methylation, $-\log_{10}(k_X/k_H)$ obtained in acetone-d_6 plotted against the values of $-\log_{10}(K_X/K_H)$ for the **1**–**X** series of molecular balances obtained in **(A)** DMSO-d_6, **(B)** ethanol, **(C)** ethyl acetate, **(D)** methanol-d_4, **(E)** THF-d_8 and **(F)** diethyl ether.

S12. Synthetic Procedures and Standard Characterization Data

All chemicals were obtained from commercial sources and used as received. All reactions were performed under a nitrogen atmosphere and using degassed solvents unless stated otherwise. Dry solvent use is stated and such solvents were HPLC grade and collected from the departmental Solvent Purification Facility (SPS). Analytical TLC was carried out on Merck aluminium sheets coated with silica gel 60F and visualized using UV light (254 nm). Preparatory TLC was carried out on Analtech 20 x 20 cm glass mounted plates on 2000 micron silica and flash chromatography was performed using silica gel Geduran 60 (40 – 63 μm). Solvent ratios have been indicated in brackets. Mass spectrometry was performed by the University of Edinburgh technician-supported mass spectrometry service, using a ThermoElectron MAT XP spectrometer for EI-HRMS and ESI-HRMS.

IR spectra were obtained on neat samples using a Shimadzu IR Affinity-1 machine. Absorptions are reported in frequency of absorption (cm^{-1}). Absorptions in the fingerprint region are not reported. ^{1}H and ^{13}C NMR spectra were recorded on a 500 MHz Bruker Avance III spectrometer. ^{19}F NMR spectra were recorded on a 400 or 500 MHz Bruker Avance III spectrometer. NMR chemical shifts (δ) are reported in parts per million (ppm) relative to trimethylsilane (δ = 0 ppm) or $CDCl_3$ (^{1}H: δ = 7.26 ppm, ^{13}C: δ = 77.16 ppm) as an internal reference.[4] All ^{13}C were ^{1}H decoupled and ^{19}F spectra with poor resolution were ^{1}H decoupled. Coupling constants, J, have been reported in Hertz (Hz).

S12.1 General procedure for tetrakis(triphenylphosphine)palladium (0)-mediated coupling of 4-bromopyridine hydrochloride with phenylboronic acids

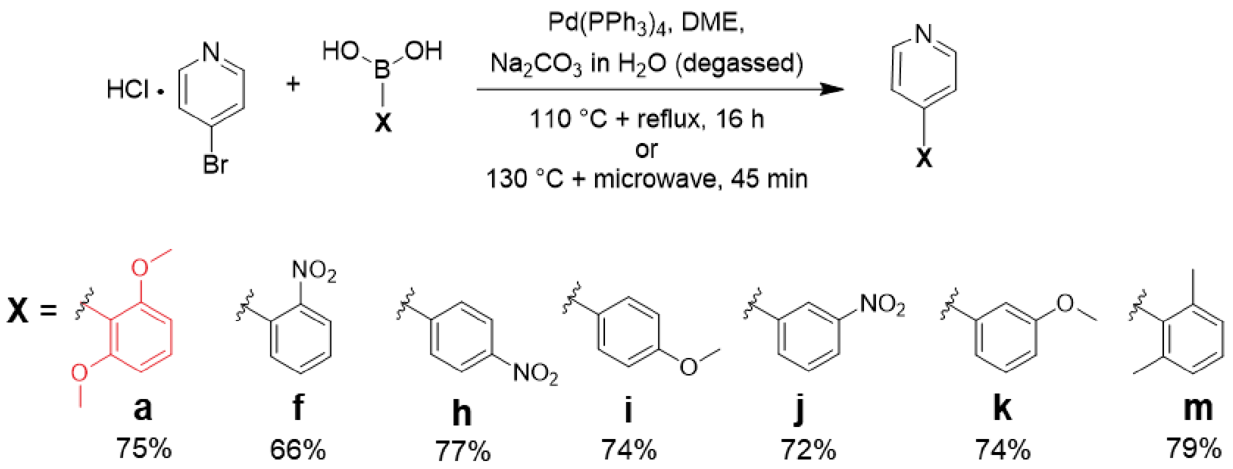

A subset of series **2–X** were prepared according to the same general tetrakis(triphenylphospine)palladium(0)-mediated cross-coupling conditions.[7] An oven dried flask was sealed before evacuating and back filling with nitrogen three times before 4-bromopyridine hydrochloride, the phenylboronic acid derivative and $Pd(PPh_3)_4$ were added. The flask was evacuated and back filled with nitrogen a further three times. Degassed dimethoxyethane (DME) and aq. Na_2CO_3 (2M) were added *via* syringe and the reaction mixture was heated at 85 °C for 18 h under a nitrogen atmosphere. For those performed under microwave radiation, the same process was performed but in a microwave vial and not flask. Microwave reactions were heated at normal absorbency at 130 °C for 45 min (with a 2 min pre-stir). The reaction mixture was then cooled to ambient temperature, quenched with sat. aq. NH_4Cl and extracted with $CHCl_3$. The combined organic extracts were dried over $MgSO_4$ before concentration *in vacuo*. The resulting products were further purified by chromatography.

4-(2,6-dimethoxyphenyl)pyridine (2–a)

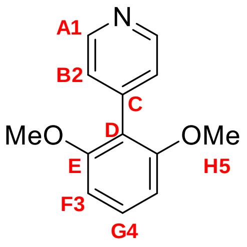

Prepared using the general tetrakis(triphenylphospine)palladium (0) mediated coupling procedure: 4-bromopyridine hydrochloride (388.5 mg, 2.0 mmol), $Pd(Ph_3P)_4$ (188.7 mg, 0.2 mmol), (2,6-dimethoxyphenyl)boronic acid (145.7 mg, 2.5 mmol), DME (40 mL) and aq. Na_2CO_3 (2 M, 7 mL). Purified with flash chromatography (1:1 petroleum ether (60/80): diethyl ether) to yield **2–a** as a yellow solid (394.5 mg, 1.83 mmol, 75%).

υ_{max} (neat) /cm^{-1} 2934.23, 1603.09, 1588.83, 1470.45, 1431.94, 1247.96, 1106.76, 820.09, 781.48; ^{1}H NMR (500 MHz, $CDCl_3$) δ 8.66 – 8.61 (1, m, 1H), 7.34 (4, t, J = 8.4 Hz, 1H), 7.33 – 7.28 (2, m, 1H), 6.68 (3, d, J = 8.4 Hz, 1H), 3.76 (5, s, 3H); ^{13}C NMR (126 MHz, $CDCl_3$) δ 157.34 (E), 149.10 (A), 142.66 (C), 129.81 (G), 126.18 (B), 116.73 (D), 104.17 (F), 55.84 (H); EI-HRMS: obtained *m/z* 215.09454 M^+ (expected *m/z* 215.09408 M^+).

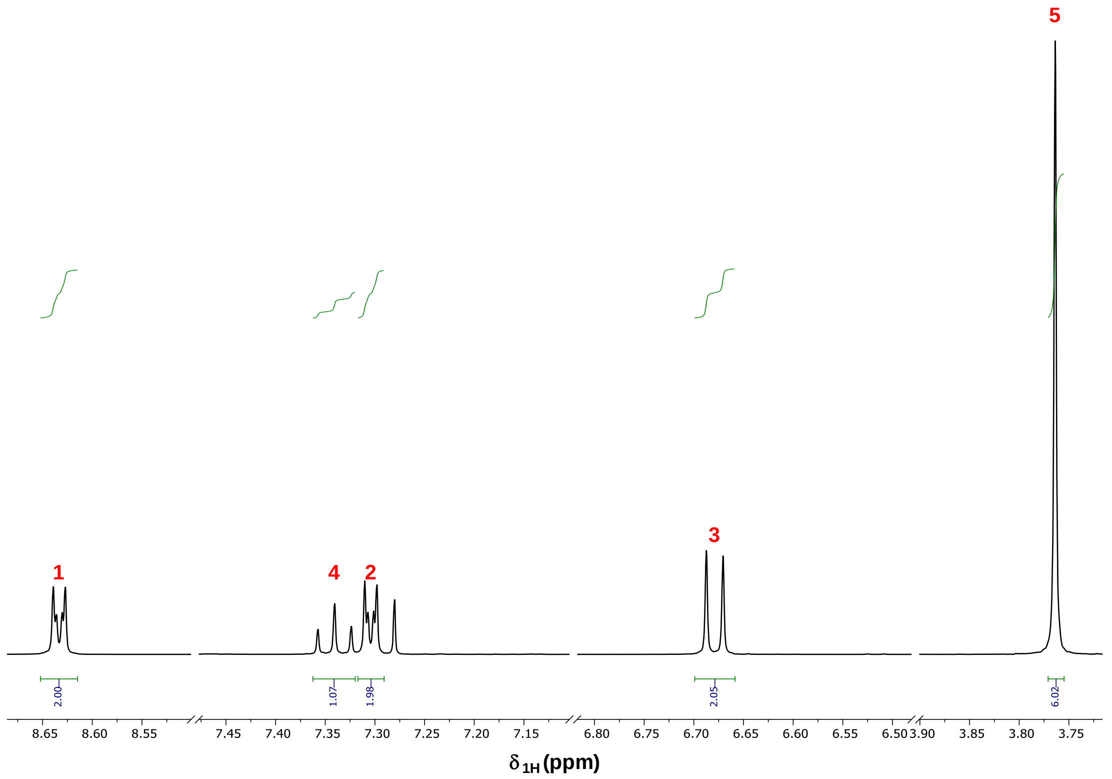

4-(2-nitrophenyl)pyridine (2−f)

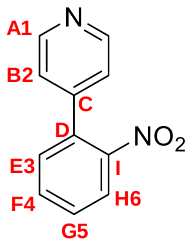

Prepared using the general tetrakis(triphenylphospine)palladium (0) mediated coupling procedure (microwave): 4-bromopyridine hydrochloride (150.2 mg, 0.77 mmol), $Pd(Ph_3P)_4$ (26.7 mg, 0.02 mmol), (2-nitrophenyl)boronic acid (180.7 mg, 1.08 mmol) DME (3.5 mL) and aq. Na_2CO_3 (2 M, 2 mL). Purified with flash chromatography (4:1 *n*-Hex: EtOAc) to yield **2−f** as a yellow solid (102.3 mg, 0.51 mmol, 66%).

υ_{max} (neat) /cm^{-1} 3035.04, 3025.06, 1593.11, 1517.52, 1352.08,854.32, 738.79; ^{1}H NMR (500 MHz, $CDCl_3$) δ 8.74 – 8.62 (1, m, 1H), 8.01 (6, dd, J = 8.2, 1.4 Hz, 1H), 7.71 (5, td, J = 7.6, 1.3 Hz, 1H), 7.65 – 7.57 (4, m, 1H), 7.44 (3, dd, J = 7.6, 1.4 Hz, 1H), 7.28 – 7.25 (2, m, 1H); ^{13}C NMR (126 MHz, $CDCl_3$) δ 150.04 (A), 148.57 (I), 145.79 (D), 134.07 (C), 132.95 (G), 131.54 (E), 129.50 (F), 124.63 (H), 122.79 (B); EI-HRMS: obtained *m/z* 200.05737 M$^+$ (expected *m/z* 200.05803 M$^+$).

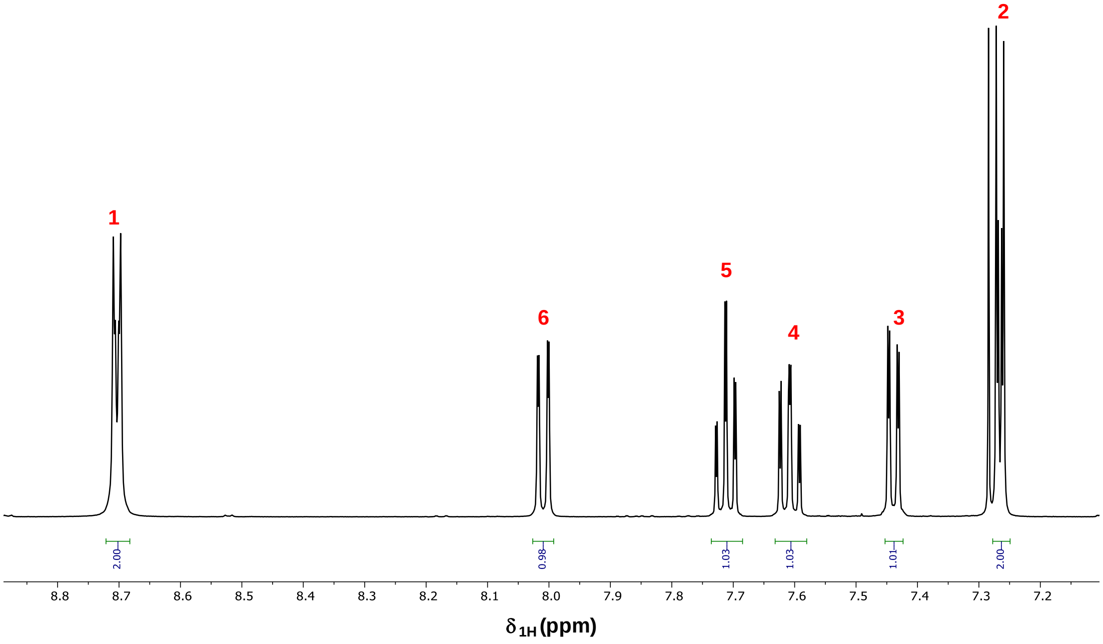

4-(4-nitrophenyl)pyridine (2−h)

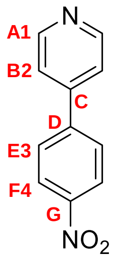

Prepared using the general tetrakis(triphenylphospine)palladium (0) mediated coupling procedure (microwave): 4-bromopyridine hydrochloride (250.6 mg, 1.29 mmol), $Pd(Ph_3P)_4$ (44.4 mg, 0.04 mmol), (4-nitrophenyl)boronic acid (300.5 mg, 1.80 mmol) DME (2.5 mL) and aq. Na_2CO_3 (2 M, 1.5 mL). Purified with flash chromatography (EtOAc) to yield **2−h** as a yellow solid (197.8 mg, 0.99 mmol, 77%).

υ_{max} (neat) /cm^{-1} 3094.94, 3035.04, 2119.51, 2851.05, 1591.68, 1513.24, 1364.59, 854.32, 812.96, 768.05; ^{1}H NMR (500 MHz, $CDCl_3$) δ 8.80 – 8.75 (1, m, 2H), 8.42 – 8.35 (4, m, 2H), 7.85 – 7.79 (3, m, 2H), 7.58 – 7.54 (2, m, 2H); ^{13}C NMR (126 MHz, $CDCl_3$) δ 150.72 (A), 148.27 (G), 146.01 (C), 144.55 (D), 128.04 (E), 124.41 (F), 121.75 (B); EI-HRMS: obtained *m/z* 200.05731 M$^+$ (expected *m/z* 200.05803 M$^+$).

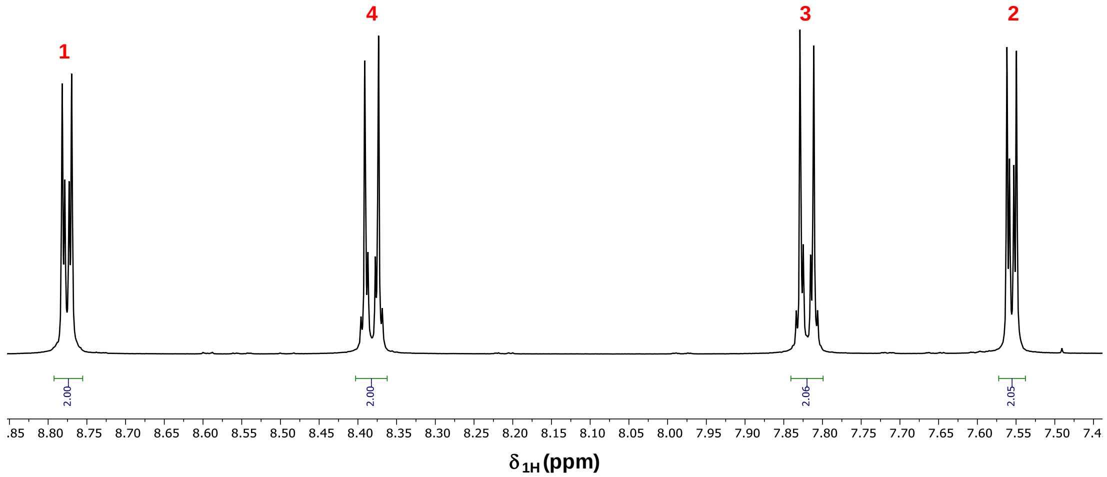

4-(4-methoxyphenyl)pyridine (2–i)

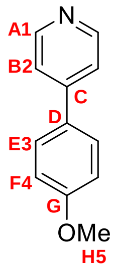

Prepared using the general tetrakis(triphenylphospine)palladium (0) mediated coupling procedure (microwave): 4-bromopyridine hydrochloride (150.2 mg, 0.77 mmol), $Pd(Ph_3P)_4$ (26.8 mg, 0.02 mmol), (4-methoxyphenyl)boronic acid (169.5 mg, 1.08 mmol) DME (3.5 mL) and aq. Na_2CO_3 (2 M, 2 mL). Purified with flash chromatography (2:2:1 petroleum ether (60/80): diethyl ether: EtOAc) to yield **2–i** as a pale yellow solid (106.1 mg, 0.57 mmol, 74%).

υ_{max} (neat) /cm^{-1} 3037.89, 2968.01, 2843.92, 1604.52, 1593.11, 1521.80, 1486.14, 1285.04, 1255.09, 1223.71, 1186.63, 1034.02, 1015.48, 805.83; ^{1}H NMR (500 MHz, $CDCl_3$) δ 8.66 – 8.61 (1, m, 2H), 7.65 – 7.59 (3, m, 2H), 7.51 – 7.47 (2, m, 2H), 7.06 – 7.01 (4, m, 2H), 3.89 (5, s, 3H); ^{13}C NMR (126 MHz, $CDCl_3$) δ 160.58 (G), 150.18 (A), 147.88 (C), 130.39 (D), 128.18 (E), 121.10 (B), 114.58, 55.42 (H); EI-HRMS: obtained *m/z* 185.08282 M$^+$ (expected *m/z* 185.08352 M$^+$).

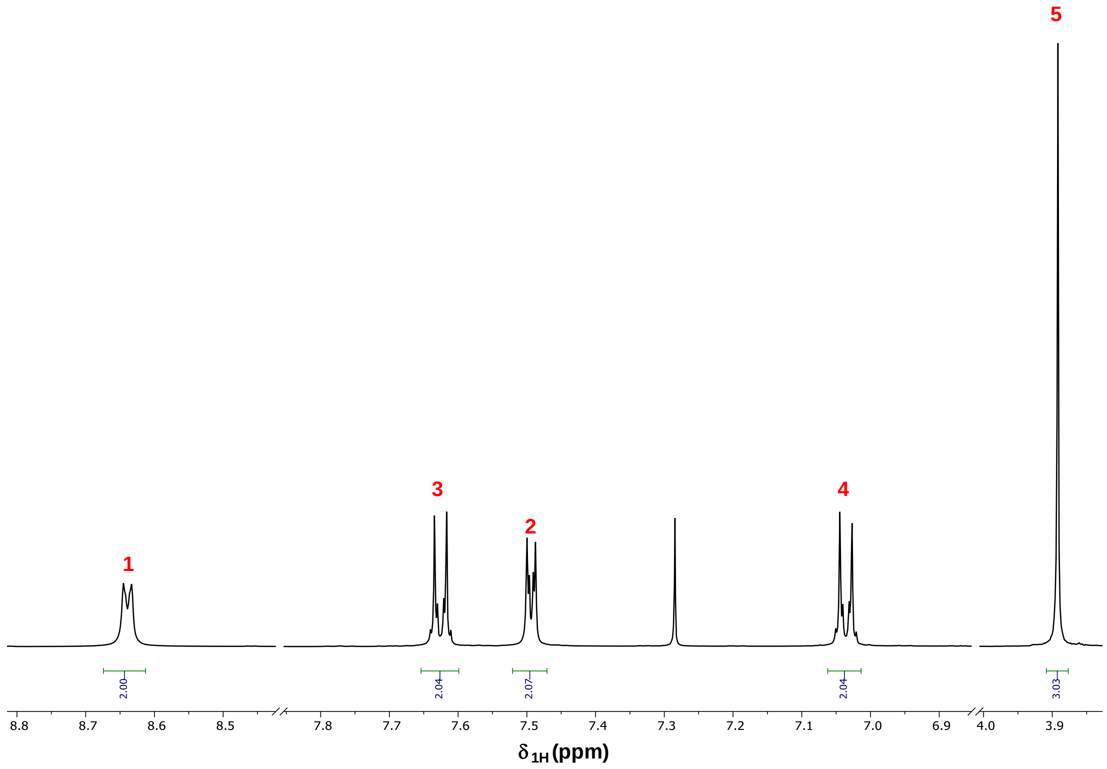

4-(3-nitrophenyl)pyridine (2–j)

Prepared using the general tetrakis(triphenylphospine)palladium (0) mediated coupling procedure

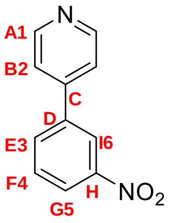

(microwave): 4-bromopyridine hydrochloride (150.2 mg, 0.77 mmol), $Pd(Ph_3P)_4$ (26.7 mg, 0.02 mmol), (2-nitrophenyl)boronic acid (180.7 mg, 1.08 mmol) DME (3.5 mL) and aq. Na_2CO_3 (2 M, 2 mL). Purified with flash chromatography (4:1 *n*-Hex: EtOAc) to yield **2–j** as a yellow solid (112.5 mg, 0.56 mmol, 72%).

υ_{max} (neat) /cm^{-1} 3089.24, 2922.37, 1595.96, 1524.65, 1500.40, 1347.80, 879.99; ^1H NMR (500 MHz, $CDCl_3$) δ 8.80 – 8.73 (1, m, 2H), 8.55 – 8.52 (6, m, 1H), 8.33 (5, ddd, J = 8.2, 2.2, 1.1 Hz, 1H), 7.99 (3, ddd, J = 7.7, 1.8, 1.0 Hz, 1H), 7.72 (4, td, J = 7.9, 0.5 Hz, 1H), 7.61 – 7.56 (2, m, 2H); ^{13}C NMR (126 MHz, $CDCl_3$) δ 150.76 (A), 148.94 (H), 145.87 (C), 139.98 (F), 132.90 (E), 130.26 (D), 123.76 (G), 122.03 (I), 121.58 (B); EI-HRMS: obtained *m/z* 200.05875 M^+ (expected *m/z* 200.05803 M^+).

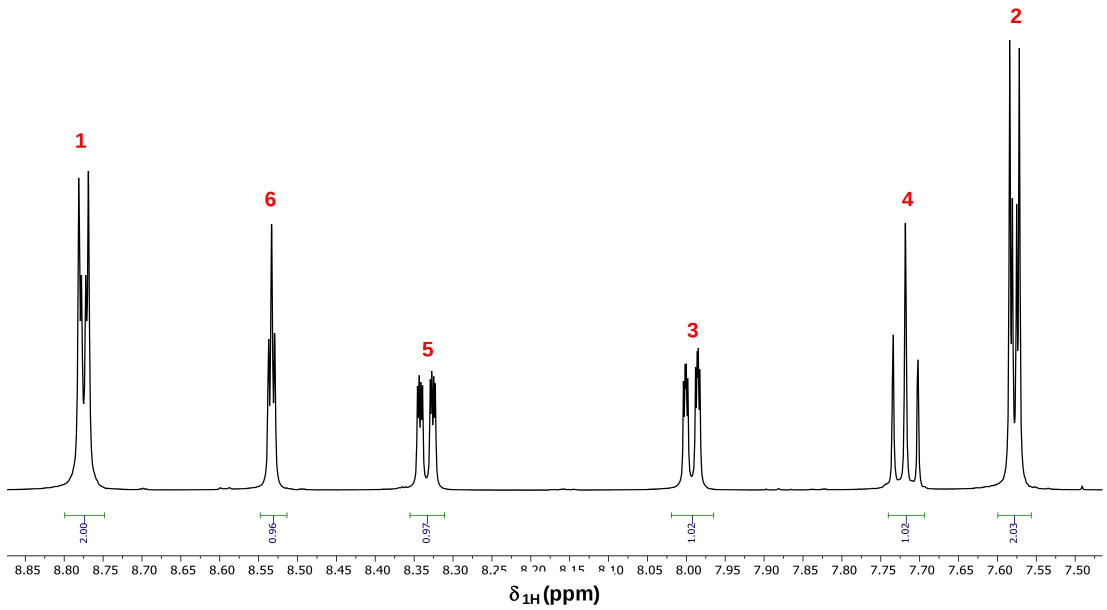

4-(3-methoxyphenyl)pyridine (2–k)

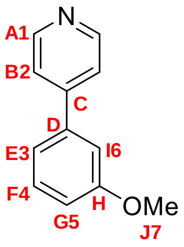

Prepared using the general tetrakis(triphenylphospine)palladium (0) mediated coupling procedure: 4-bromopyridine hydrochloride (388.0 mg, 2.0 mmol), $Pd(Ph_3P)_4$ (189.7 mg, 0.2 mmol), (3-methoxyphenyl)boronic acid (450.9 mg, 2.5 mmol) DME (40 mL) and aq. Na_2CO_3 (2 M, 7 mL). Purified with flash chromatography (1:1 petroleum ether (40/60): diethyl ether) to yield **2–k** as a pale yellow solid (274.7 mg, 1.83 mmol, 74%).

υ_{max} (neat) /cm^{-1} 3681.83, 2966.58, 2843.92, 1594.54, 1583.13, 1546.04, 1477.58, 1302.16, 1053.99, 1035.39, 792.99; ^{1}H NMR (500 MHz, $CDCl_3$) δ 8.72 – 8.64 (1, m, 2H), 7.55 – 7.50 (2, m, 2H), 7.43 (4, t, J = 8.0 Hz, 1H), 7.25 (5, ddd, J = 7.7, 1.7, 0.9 Hz, 1H), 7.18 (6, dd, J = 2.6, 1.7 Hz, 1H), 7.01 (3, ddd, J = 8.3, 2.6, 1.0 Hz, 1H), 3.90 (7, s, 3H); ^{13}C NMR (126 MHz, $CDCl_3$) δ 160.21 (H), 150.27 (A), 148.28 (C), 139.69 (D), 130.21 (F), 121.73 (B), 119.45 (G), 114.37 (E), 112.84 (I), 55.41 (J); EI-HRMS: obtained *m/z* 185.08368 M$^+$ (expected *m/z* 185.08352 M$^+$).

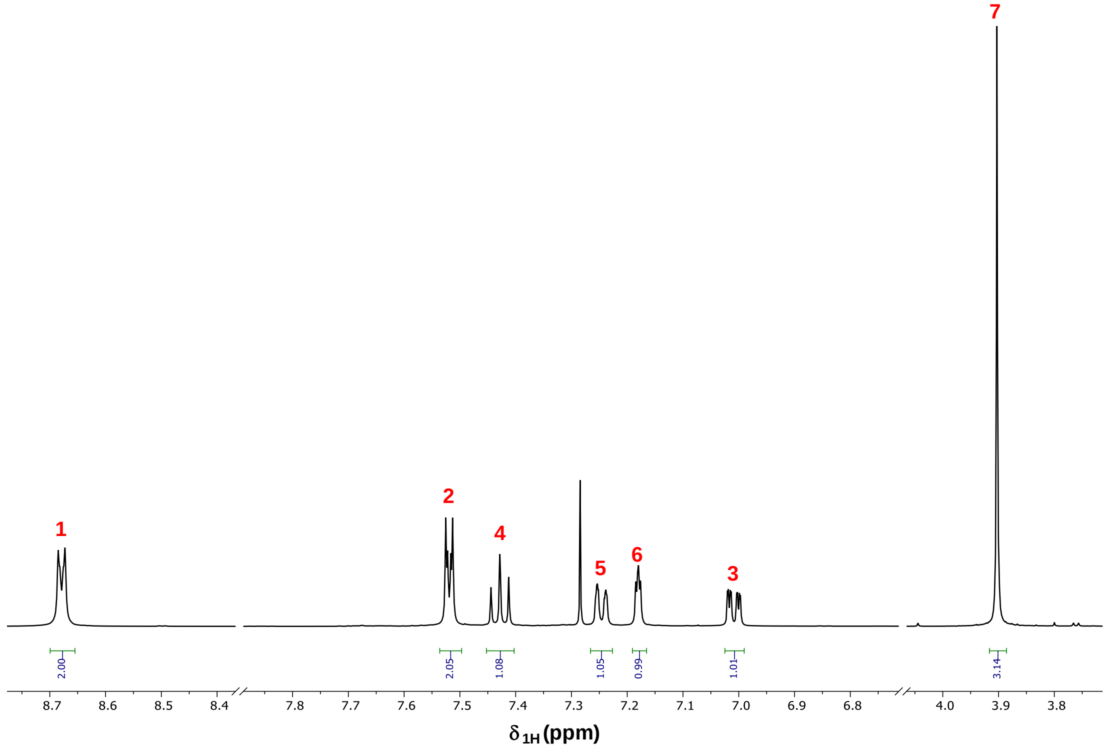

4-(2,6-dimethylphenyl)pyridine (2‒m)

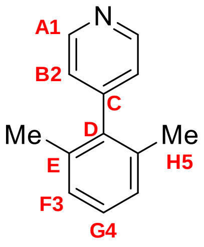

Prepared using the general tetrakis(triphenylphospine)palladium (0) mediated coupling procedure (microwave): 4-bromopyridine hydrochloride (200.2 mg, 1.0 mmol), $Pd(Ph_3P)_4$ (35.8 mg, 0.03 mmol), (2,6-dimethylphenyl)boronic acid (215.6 mg, 1.44 mmol) DME (4 mL) and aq. Na_2CO_3 (2 M, 1.5 mL). Purified with flash chromatography (2:1 petroleum ether (60/80): EtOAc) to yield **2‒m** as a white solid (149.3 mg, 1.83 mmol, 79%).

υ_{max} (neat) /cm^{-1} 3284.63, 2920.94, 1604.52, 1541.77, 1464.75, 1404.85, 1354.93, 1212.30, 825.79, 780.15; ^{1}H NMR (500 MHz, $CDCl_3$) δ 8.70 (d, J = 5.1 Hz, 2H), 7.23 (4, dd, J = 8.3, 6.8 Hz, 1H), 7.16 – 7.13 (2 and 3, m, 4H), 2.05 (5, s, 6H); ^{13}C NMR (126 MHz, $CDCl_3$) δ 150.07 (A), 149.51 (C), 139.04 (E), 135.20 (D), 127.90 (G), 127.60 (F), 124.54 (B), 20.64 (H); EI-HRMS: obtained *m/z* 183.10453 M^+ (expected *m/z* 183.10425 M^+).

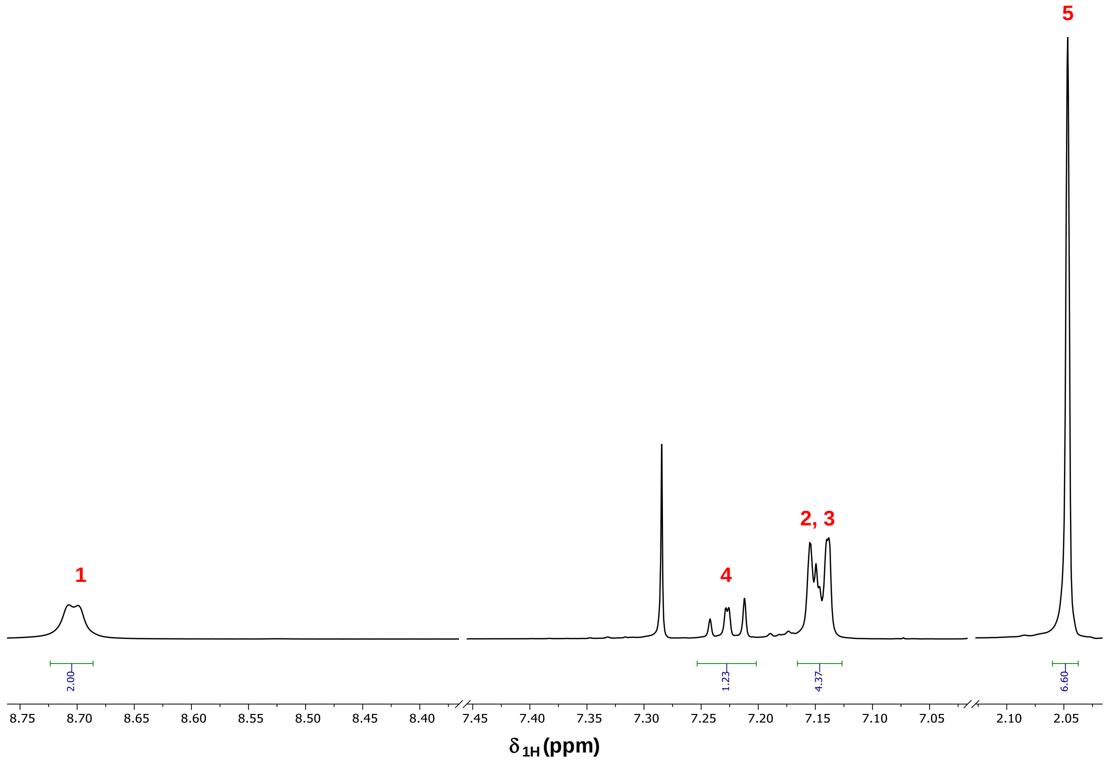

S12.2 Tri(dibenzylideneactone)dipalladium (0)-mediated coupling of 4-bromopyridine hydrochloride with (2-methoxy)phenylboronic acid

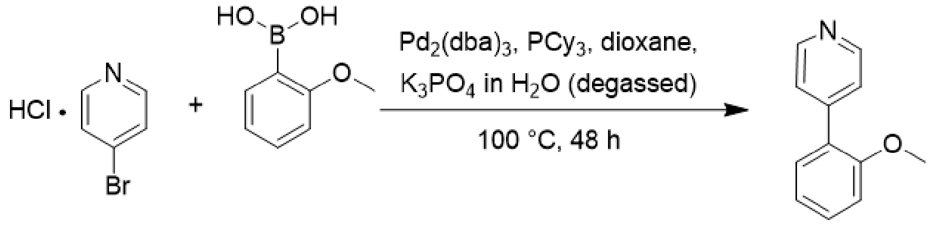

2–g
63%

Compound **2–g** was prepared according to the same general tri(dibenzylideneacetone)dipalladium (0) mediated cross-coupling conditions.[8] An oven dried flask was sealed before evacuating and back filling with nitrogen three times before 4-bromopyridine hydrochloride, the phenylboronic acid derivative, $Pd_2(dba)_3$ and PCy_3 were added. The flask was evacuated and back filled with nitrogen a further three times. Degassed dioxane and aq. K_3PO_4 (2M) were added *via* syringe and the reaction mixture was heated at 100 °C for 48 h under a nitrogen atmosphere. The reaction mixture was filtered through a silica pad, eluting with EtOAc then washed with $NaHCO_3$ and brine. The combined organic extracts were dried over $MgSO_4$ before concentration *in vacuo*. The resulting products were further purified by flash column chromatography.

4-(2-methoxyphenyl)pyridine (2–g)

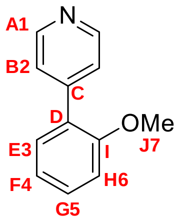

Prepared using the general tri(dibenzylideneacetone)dipalladium (0) mediated coupling procedure: 4-bromopyridine hydrochloride (389.1 mg, 1.99 mmol), $Pd_2(dba)_3$ (36.5 mg, 0.04 mmol), (2-methoxyphenyl)boronic acid (1344.4 mg, 2.19 mmol), dioxane (6 mL) and aq. K_3PO_4 (2 M, 3 mL). Purified with flash chromatography (1:1 petroleum ether (60/80): diethyl ether) to yield **2–g** as a pale yellow solid (252.6 mg, 1.36 mmol, 68 %).

υ_{max} (neat) /cm^{-1} 3623.65, 3016.50, 2966.58, 2843.92, 1605.95, 1590.26, 1483.29, 1456.19, 1232.27, 1056.84, 1032.60, 1016.91, 827.22, 758.64; ^{1}H NMR (500 MHz, $CDCl_3$) δ 8.67 – 8.58 (1, m, 2H), 7.52 – 7.47 (2, m, 2H), 7.42 (4, ddd, J = 8.3, 7.4, 1.7 Hz, 1H), 7.37 (6, dd, J = 7.6, 1.7 Hz, 1H), 7.09 (5, td, J = 7.5, 1.1 Hz, 1H), 7.04 (3, dd, J = 8.3, 1.0 Hz, 1H), 3.86 (7, s, 3H); ^{13}C NMR (126 MHz, $CDCl_3$) δ 156.58 (I), 149.51 (A), 146.35 (D), 130.50 (H), 130.13 (F), 127.74 (C), 124.33 (B), 121.09 (G), 111.45 (E), 55.57 (J); EI-HRMS: obtained *m/z* 185.08447 M$^+$ (expected *m/z* 185.08352 M$^+$).

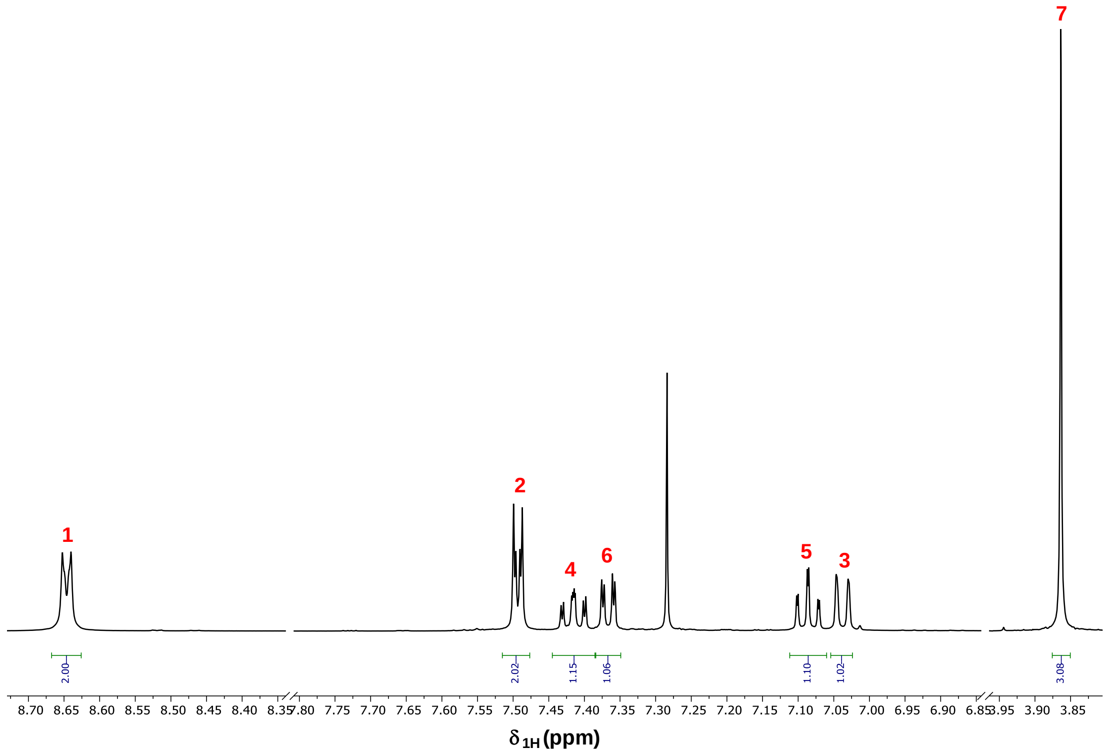

S12.3 General procedure for tri(dibenzylideneactone)dipalladium (0)-mediated coupling of 4-pyridylboronic acid with halobenzene derivatives

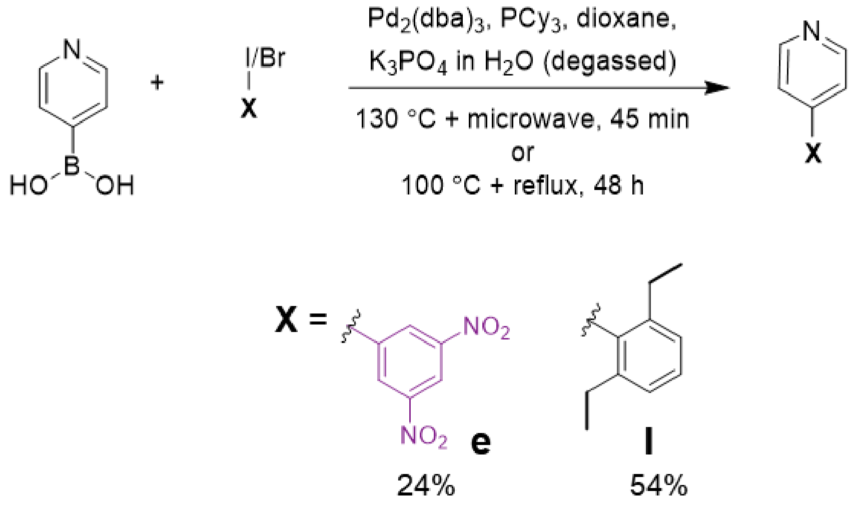

Compounds **2–e** and **2–l** were prepared according to the same general tri(dibenzylideneacetone)dipalladium (0) mediated cross-coupling conditions.[8] An oven dried microwave vial/flask was sealed before evacuating and back filling with nitrogen three times before 4-pyridylboronic acid, the halobenzene derivative, $Pd_2(dba)_3$ and PCy_3 were added. The vial/ flask was evacuated and back filled with nitrogen a further three times. Degassed dioxane and aq. K_3PO_4 (2M) were added *via* syringe and the reaction mixture was heated at 100 °C for 48 h or 130 °C for 45 min (microwave) under a nitrogen atmosphere. The reaction mixture was filtered through a silica pad, eluting with EtOAc then washed with $NaHCO_3$ and brine. The combined organic extracts were dried over $MgSO_4$ before concentration *in vacuo*. The resulting products were further purified by flash column chromatography.

4-(3,5-dinitrophenyl)pyridine (2–e)

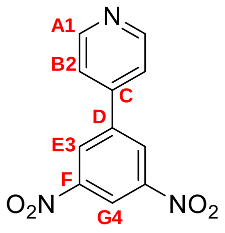

Prepared using the general tri(dibenzylideneacetone)dipalladium (0) mediated coupling procedure 4-pyridylboronic acid (137.9 mg, 1.12 mmol), $Pd_2(dba)_3$ (18.3 mg, 0.01 mmol), PCy_3 (13.6 mg, 0.03 mmol), 2-iodo-3,5-dinitrobenzene (299.8 mg, 1.02 mmol), dioxane (6 mL) and aq. K_3PO_4 (2 M, 2 mL). Purified with flash chromatography (4:1 *n*-Hex: EtOAc) to yield **2–e** as a yellow solid (67.7 mg, 0.28 mmol, 24%).

υ_{max} (neat) /cm^{-1} 3881.13, 2973.71, 2865.32, 1595.96, 1533.21, 1346.37, 1053.99, 1032.60, 824.37, 728.81; ^1H NMR (500 MHz, $CDCl_3$) δ 9.13 (4, t, J = 2.0 Hz, 0H), 8.85 (1, d, J = 5.6 Hz, 1H), 8.82 (3, d, J = 2.0 Hz, 1H), 7.62 (2, d, J = 6.1 Hz, 1H); ^{13}C NMR (126 MHz, $CDCl_3$) δ 151.20 (A), 149.22 (F), 143.55 (D), 141.95 (C), 127.01 (E), 121.39 (B), 118.80 (G); EI-HRMS: obtained *m/z* 245.04401 M^+ (expected *m/z* 245.04311 M^+).

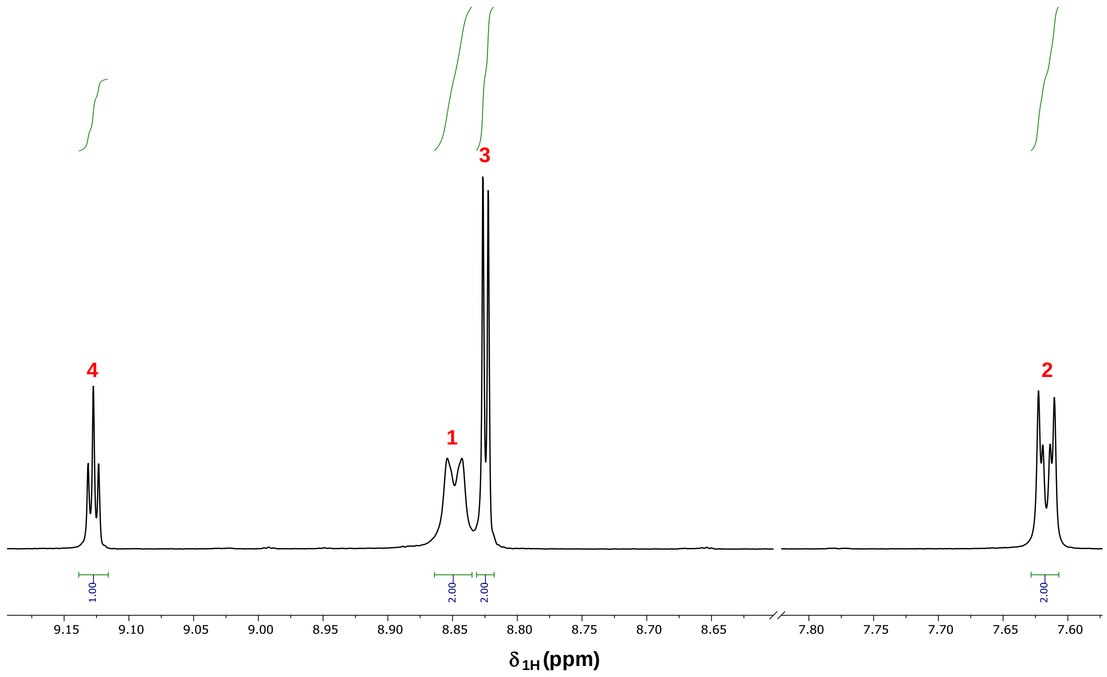

4-(2,6-diethylphenyl)pyridine (2–l)

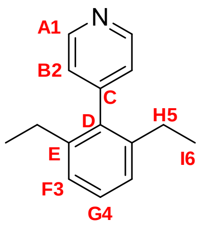

Prepared using the general tri(dibenzylideneacetone)dipalladium (0) mediated coupling procedure (microwave): 4-pyridylboronic acid (88.1 mg, 0.72 mmol), $Pd_2(dba)_3$ (12.6 mg, 0.02 mmol), PCy_3 (9.4 mg, 0.05 mmol), 2,6-diethylbromobenzene (0.11 mL, 0.66 mmol), dioxane (2.5 mL) and aq. K_3PO_4 (2 M, 1.33 mL). Purified with flash chromatography (4:1 *n*-Hex: EtOAc) to yield **2–l** as a white solid (82.4 mg, 0.39 mmol, 54%).

υ_{max} (neat) /cm^{-1} 270.86, 2963.73, 2932.35, 2869.59, 1598.82, 1453.34, 1403.42, 1213.73, 989.81, 822.94, 808.68, 764.46; ^{1}H NMR (500 MHz, $CDCl_3$) δ 8.67 (1, d, J = 4.5 Hz, 1H), 7.31 (4, t, J = 7.7 Hz, 1H), 7.15 (2 and 3, m, 4H), 2.30 (5, q, J = 7.6 Hz, 4H) 1.02 (6, t, J = 7.5 Hz, 6H); ^{13}C NMR (126 MHz, $CDCl_3$) δ 149.73 (A), 148.91 (D), 141.41 (G), 137.94 (E), 128.31 (F), 125.84 (B), 124.97 (C), 26.70 (H), 15.55 (I); EI-HRMS: obtained *m/z* 211.13612 M$^+$ (expected *m/z* 211.13555 M$^+$).

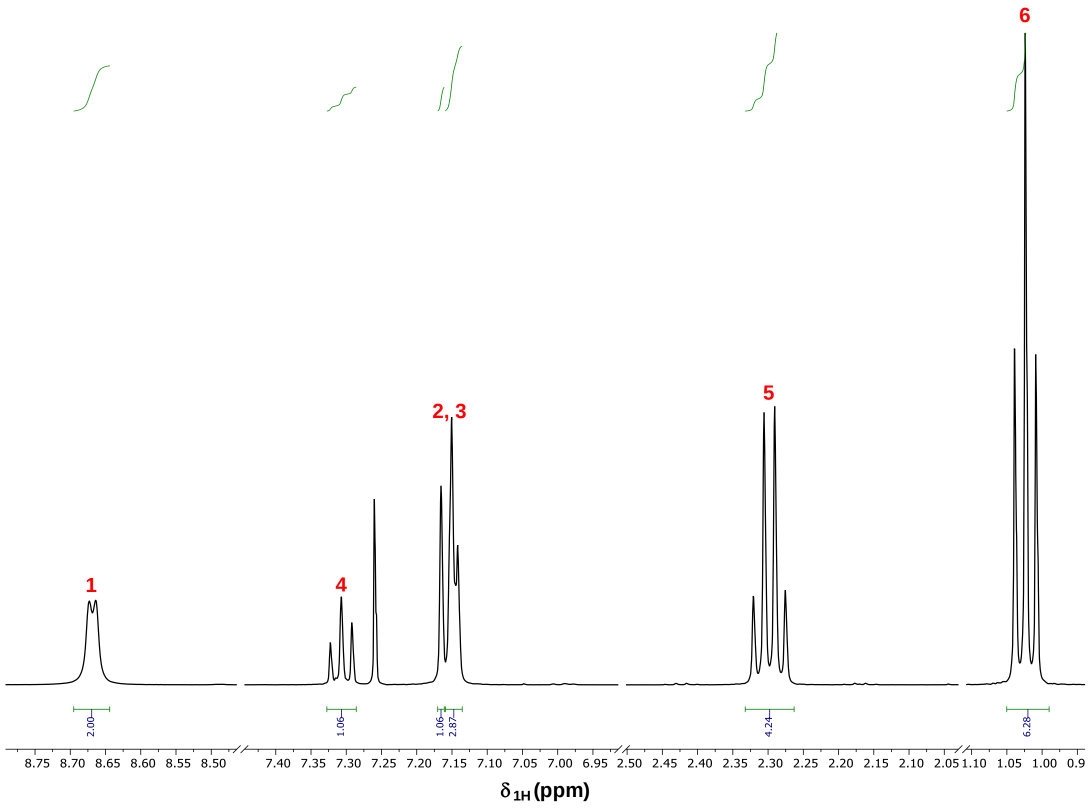

S13. References for Section B

1. Mati, I. K. Molecular torsion balances for quantifying non-covalent interactions. The University of Edinburgh, 2013.

2. Mati, I. K.; Adam, C.; Cockroft, S. L., Seeing through solvent effects using molecular balances. *Chem. Sci.* **2013,** *4*, 3965 - 3972.

3. Muchowska, K. B.; Adam, C.; Mati, I. K.; Cockroft, S. L., Electrostatic Modulation of Aromatic Rings via Explicit Solvation of Substituents. *J. Am. Chem. Soc.* **2013,** *135* (27), 9976 - 9979.

4. Fulmer, G. R.; Miller, A. J. M.; Sherden, N. H.; Gottlieb, H. E.; Nudelman, A.; Stoltz, B. M.; Bercaw, J. E.; Goldberg, K. I., NMR Chemical Shifts of Trace Impurities: Common Laboratory Solvents, Organics, and Gases in Deuterated Solvents Relevant to the Organometallic Chemist. *Organometallics* **2010,** *29* (9), 2176-2179.

5. Hansch, C.; Leo, A.; Taft, R. W., A survey of Hammett substituent constants and resonance and field parameters. *Chem. Rev.* **1991,** *91* (2), 165-95.

6. McDaniel, D. H.; Brown, H. C., An Extended Table of Hammett Substitutent Constants Based on the Ionization of Substituted Benzoic Acids. *J. Org. Chem.* **1958,** *23* (3), 420-427.

7. Leão Lana, E. J.; Carazza, F.; Aparacida de Oliveira, R., Synthesis of 2-Aryl- and 2-Heteroaryl-3,5-dimethoxy-1,4-benzoquinones Involving Pd-Catalyzed Cross-Coupling of (2,3,4,6-Tetramethoxyphenyl)boronic Acid. *Helv. Chim. Acta* **2004,** *87* (7), 1825-1831.

8. Kudo, N.; Perseghini, M.; Fu, G. C., A Versatile Method for Suzuki Cross-Coupling Reactions of Nitrogen Heterocycles. *Angew. Chem. Inter. Ed.* **2006,** *45* (8), 1282-1284.

9. Ashton, P. R.; Harris, K. D. M.; Kariuki, B. M.; Philp, D.; Robinson, J. M. A.; Spencer, N., A borazaaromatic analogue of isophthalic acid. *J. Chem. Soc., Perkin Transactions 2* **2001,** (11), 2166-2173.

10. Nishimura, N.; Yoza, K.; Kobayashi, K., Guest-Encapsulation Properties of a Self-Assembled Capsule by Dynamic Boronic Ester Bonds. *J. Am. Chem. Soc.* **2010,** *132* (2), 777-790.

11. Akagi, S.; Yasukawa, Y.; Kobayashi, K.; Konishi, H., Synthesis and solid state structure of oxacalix[4]arenes bearing four nitro groups and four tert-butyl groups at their extra-annular positions. *Tetrahedron* **2009,** *65* (48), 9983-9988.

12. Paduraru, P. M.; Popoff, R. T. W.; Nair, R.; Gries, R.; Gries, G.; Plettner, E., Synthesis of Substituted Alkoxy Benzene Minilibraries, for the Discovery of New Insect Olfaction or Gustation Inhibitors. *J. Combi. Chem.* **2008,** *10* (1), 123-134.
